# An Eco-friendly Approach to C–H Bond Activation through Microwave Irradiation Employing Synthesized Palladium-PEPPSI-NHC Complexes

**DOI:** 10.1021/acsomega.5c04713

**Published:** 2025-08-27

**Authors:** Ichraf Slimani, İsmail Özdemir, Nevin Gürbüz, Bülent Alıcı, Nahide Burcu Arslan, Namık Özdemir

**Affiliations:** 1 Catalysis Research and Application Center, 37520 İnönü University, Malatya 44280, Turkey; 2 Faculty of Science and Arts, Department of Chemistry, 37520 İnönü University, Malatya 44280, Turkey; 3 Department of Physics, Faculty of Science and Arts, 187438 Giresun University, Giresun 28100, Turkey; 4 Department of Physics, Faculty of Science, 162331 Ondokuz Mayıs University, Samsun 55139, Turkey

## Abstract

The formation of carbon–carbon bonds constitutes one of the most fundamental synthetic operations in organic chemistry. Arylation of heteroarenes through C–H bond activation using Pd-PEPPSI complexes as catalysts was widely performed using the classical heating method. However, the use of this heating method is associated with an unfavorable environmental profile, as they generally use a high reaction temperature, a high catalyst load, and a long reaction time. Herein, we disclose the synthesis of new Pd-PEPPSI-NHC complexes bearing NHC ligands, which were tested as a catalyst in the arylation of 2-acethylfuran and 2-acethylthiophene with different aryl bromides using microwave irradiation. This novel method provides access to the biaryl scaffolds in good yields using 0.5 mol % as catalyst loading and at 110 °C. The structure of the five palladium­(II) complexes has been elucidated through NMR ^1^H, ^13^C, and FT-IR spectroscopy. Furthermore, the square-planar geometry of the organometallic ion was confirmed by single-crystal X-ray diffraction carried out on complexes **3b** and **3e**.

## Graphical abstract

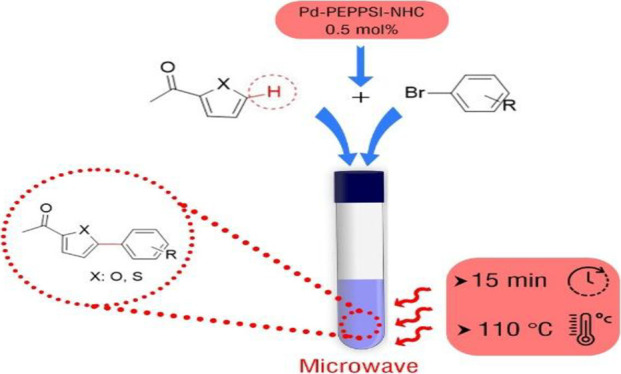

## 1 Introduction

The concept of green chemistry is gaining importance, as the concept of sustainability has become a key principle in many scientific and technical fields in recent years.^1^ Attention is therefore being given to developing resource-efficient synthetic chemicals to transform basic molecules into highly functionalized compounds with high biopotential.

One of the principles of green chemistry, catalysis, has proven to be an effective means of meeting sustainability requirements, including high yield, selectivity, atom economy, and reaction efficiency.^2^ The creation of C–C bonds is an important area of study in organic chemistry. These bonds were previously created by interconverting substrates containing heteroatoms or unsaturated groups.^3,4^ It was demonstrated that homogeneous transition metal-catalyzed cross-coupling reactions were the most successful of all the various strategies.^5–8^

The significance of this approach, which remains the preferred method for the formation of C–C bonds, was recognized by the 2010 Nobel Prize in Chemistry, which was given to Suzuki, Negishi, and Heck for ″palladium-catalyzed cross-couplings in organic synthesis″.^9^ Transition metal-catalyzed direct arylation via C–H bond activation, which entails coupling an arene (Ar–H) with an aryl halide (Ar–X), has been extensively studied in recent years as an alternative to conventional cross-coupling reactions.^10^ This new approach has become a more potent molecular synthesis platform, opening up possibilities in the pharmaceutical, material sciences, and natural product synthesis sectors.

The main advantage of the direct arylation coupling reaction is that it minimizes the need for prefunctionalization heteroarenes by directly employing the C–H bonds of heteroarenes derivatives as coupling partners to create aryl–aryl structures.^11–16^ This approach produces only nontoxic wastes, enhances atom economy, and optimizes preparation and purification.

Furthermore, simple^17^ and activated arene, including sulfonyl chloride, triflate, mesylates, aryl halides, and aryl organometallic compounds,^18–22^ were activated using the direct arylation approach. However, due to their low cost and affordability, aryl halides are utilized widely among them. Various industrially significant chemicals, as well as organic compounds that are biologically active and functional materials, contain the biaryl unit, which is created by directly arylating heteroarenes with aryl halides.^23^

Compounds containing furan, thiophene, or thiazole derivative units exhibit significant biological activity and are of interest in pharmaceutical chemistry. For example, dantrolene^24^ is a neuromuscular agent that helps relax specific body muscles; azimilide^25^ is a class IΙΙ antiarrhythmic drug that helps control abnormal heart rhythms; nifuroxazide^26,27^ is an antibiotic that is indicated in the treatment of susceptible gastrointestinal infections; articaine^28^ is a local anesthetic that is used to induce local or conductive anesthesia for dental procedures; canagliflozin^23^ is used to manage hyperglycemia in type two diabetes and reduce the risk of significant cardiovascular events; and prasugrel^29^ is an anticoagulant used to minimize the risk of heart attacks (Figure 1). Because of these properties, the discovery of simple and direct routes to access heteroarenes derivatives using a simple catalytic system remains an important challenge for organic chemists.

**Figure 1:**
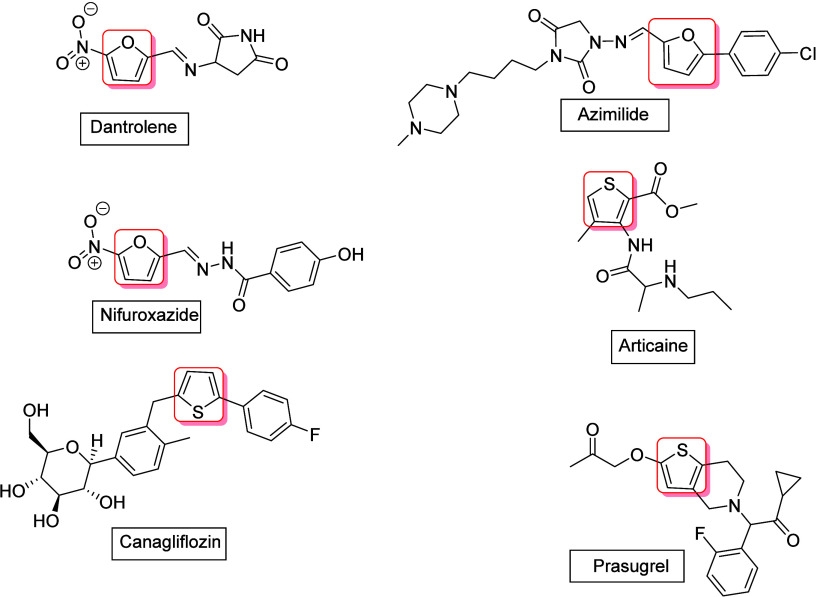
Examples of bioactive derivative products of furans and thiophenes.

Pd-NHC-catalyzed couplings have garnered the most attention, despite the fact that complexes of diverse metals using *N*-hetrocyclic carbene as ligands have been employed as catalysts in cross-coupling activities. Pd-NHCs have been used as a catalyst in a number of important reactions, including Heck, Suzuki–Miyaura, Negishi, Sonogashira, Kumada, Stille, and C–H activation.^30–34^ Palladium with *N*-heterocyclic carbene (NHC) was initially shown by Hermann and co-workers to have exceptional thermal stability and be suitable for catalysis in contemporary homogeneous catalysis.^35^ Later, Song et al.,^36,37^ Organ et al.,^38^ Wu et al.,^39^ Meiries et al.,^40,41^ Tu et al.,^42,43^ and others^44–49^ employ a different form of homogeneous catalysis. Thanks to their unique and easily tunable stereoelectronic properties, synthetic versatility, low toxicity, and stability to temperature, air, and light, *N*-heterocyclic carbenes (NHCs) have proved to be the best substitute for phosphine ligands.^50–53^

The PEPPSI (pyridine-enhanced precatalyst, preparation, stabilization, and initiation) type is one of the metal-NHC complexes that NHCs can produce with palladium. PEPPSI-type complexes, which were initially described in the literature by Organ et al., are composed of a metal center: palladium, two ligands: NHC ligand and pyridine ligand, and two halides.^54^ PEPPSI-type complexes are widely preferred catalysts in the creation of carbon–carbon and carbon–heteroatom bonds.^55–58^ This is due to the facile elimination of the pyridine ligand from PEPPSI compounds over the catalytic process. In addition, the significant catalytic activity of these compounds is attributed to the stability of the Pd carbene link.^59,60^

Generally, C–H bond activation proceeds using conventional heating, which means using an external thermal source.^61^ In fact, the energy is performed to satisfy the demand of C–H activation; however, during the process, significant energy is lost and its transmission into the molecule is primarily contingent upon the conductivity of the chemical reactor. As a result, the chemical process requires a longer time and becomes less effective.^62^

One alternative energy source that can be used for organic synthesis reactions is the use of microwaves. After seminal reports in 1986 by Rousell et al. and Majetic et al., the interest in microwave heating is increasing in both academic and industrial research contexts.^63,64^ In fact, the reaction mixture is locally heated by employing microwave irradiation, changing the method of transfer of heat process from conductivity to irradiation.^65^ The heating process occurs when electromagnetic waves from the microwave interact with the absorbing material, and dipole molecules and ions are forced together by an alternating electric field, causing friction, collisions, and motion.^66^

High temperatures are produced when polar or ionic molecules with permanent dipole moments couple with microwaves (frequency range 30 GHz–300 MHz) via the molecules’ relaxation time following ionic conduction and dipolar polarization.^67,68^ Organic synthesis places a high priority on this kind of heating since carbon compounds are excellent microwave absorbers.^69^ By converting electromagnetic energy into thermal energy rather than transferring it, microwave heating reduces energy loss and allows for temperature control by adjusting the microwave irradiation intensity.^70^ This method is significantly more efficient than traditional heating methods due to its uniform and consistent localization, which also shows greater yields in terms of shorter reaction times.^71,72^ In summary, microwave-heated chemistry offers several rewarding benefits including safety, reproducibility, and ease of scaling. These factors make it an appealing option for high-throughput processing and laboratory-scale medicinal chemistry. Since it can effectively reach high temperatures in a shorter time than is typically needed for the synthesis of biaryl compounds via C–H bond activation, microwave irradiation turned into an effective heating method. Therefore, complex compounds that would ordinarily require numerous stages and a significant reaction time can be synthesized easily by combining a very promising C–H activation approach with an effective heating method via microwave irradiation.

In the course of our research project synthesis of organometallics complexes, we have revealed different Pd-PEPPSI-NHC complexes, which have been tested as catalysts in C–H bond activation in order to synthesize biaryl (Scheme 1a). Generally, in Pd-PEPPSI-NHC-catalyzed arylation, heteroarenes react with different aryl bromide components.^73–75^ Nevertheless, the conditions under which this reaction was conducted were extremely aggressive. As an attractive and broadly applicable alternative to the conventional heating method, we were able to use microwave irradiation in a Pd-PEPPSI-NHC-catalyzed arylation reaction under less aggressive reaction conditions (Scheme 1b).

**Scheme 1:**
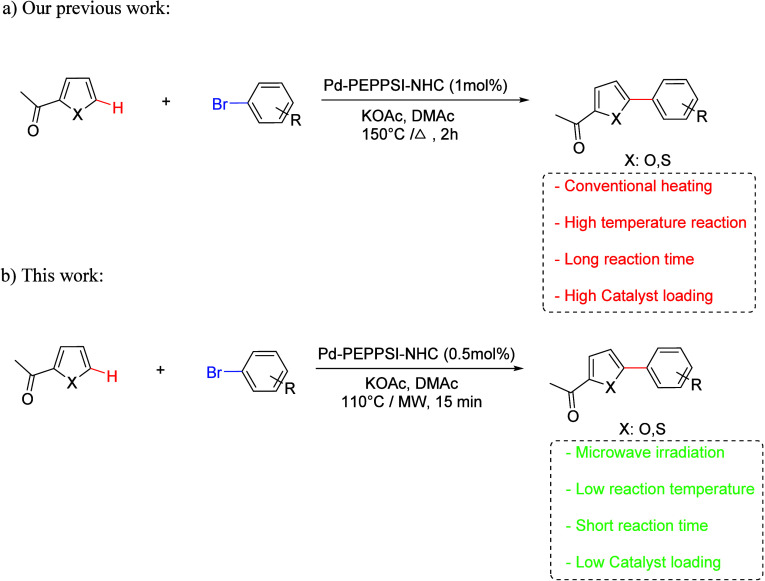
Recent Advancements for Biaryl Synthesis via C–H Bond Activation

This article details the formation of a novel palladium complex type PEPPSI (**3a**–**e**) obtained from NHC precursors. The characterization of new compounds was verified by NMR (^1^H, ^13^C) and FT-IR spectroscopy. Furthermore, the structures of complexes **3b** and **3e** were determined by X-ray diffraction. Next, the activity of palladium­(II) complexes was examined for the direct C5-arylation reaction 2-acethylfurane and 2-acethylthiophene with varied aryl bromides.

Both FT-IR and NMR (^1^H, ^13^C) spectroscopy were used to confirm the novel compounds’ characterization. Moreover, X-ray diffraction was used to determine the structures of complexes **3b** and **3e**. The direct C5-arylation reaction between acethylfuran and acethylthiophene with various aryl bromides was next investigated using palladium-PEPPSI complexes.

## 2 Results and Discussion

### 2.1 Synthesis and Characterization

Adequate benzimidazolium salts **2a**–**e** were required to be synthesized in order to synthesize the five indicated PEPPSI-Pd-NHC complexes **3a**–**e**. These salts were obtained by the alkylation of 1-((tetrahydrofuran-2-yl)­methyl)-1*H*-benzimidazole 1 with different benzyl halides (Scheme 2). FT-IR and ^1^H, ^13^C NMR spectroscopy were used to fully characterize salts **2a**–**e**, which were isolated in yields ranging from 76 to 83% (Supporting Information). PEPPSI-Pd-NHC complexes **3a**–**e** were synthesized by using N-heterocyclic carbene ligands (NHC) **2a**–**e**.

**Scheme 2:**
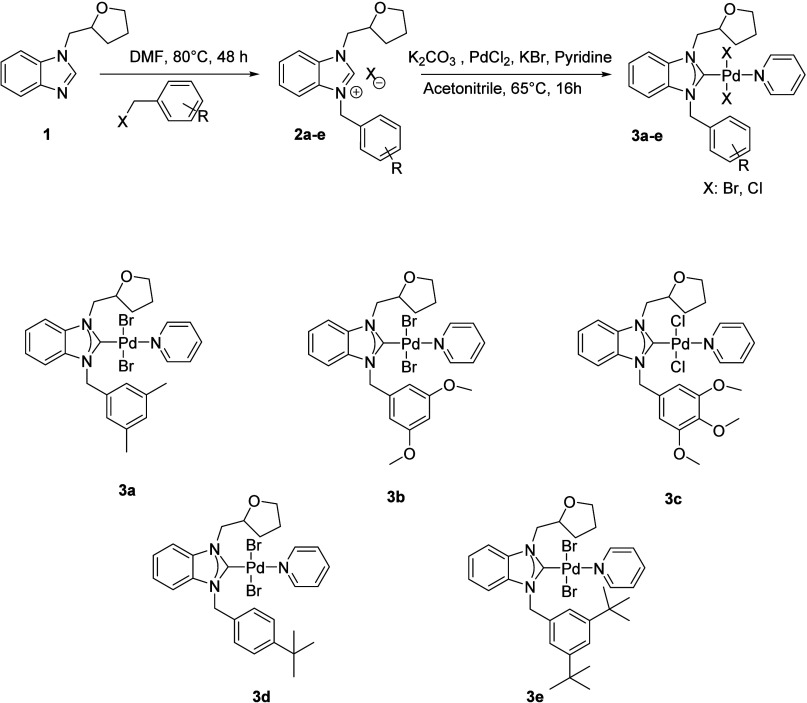
Synthesis of PEPPSI-Pd-NHC Complexes **3a**–**e**

Using a corresponding *N*-heterocyclic carbene ligand, PdCl_2_, K_2_CO_3_, and pyridine with acetonitrile over 65 °C and during 16 h, PEPPSI-Pd-NHC complexes **3a**–**e** were formed (Scheme 2). Moreover, potassium bromide (KBr) was utilized as an additive during the complexation reaction, involving a bromide salt. In either liquid- or solid-state form, ligands and complexes were stable in the presence of air, light, and moisture. Complexes had been recrystallized using dichloromethane/*n*-pentane and chloroform/*n*-pentane mixtures. For single-crystal analysis, complexes **3b** and **3e** produced adequate crystals. Each of the generated compounds’ structures has been fully characterized using FT-IR and NMR (^1^H, ^13^C) spectroscopy. At chemical shifts of 11.21, 10.01, 11.64, 11.34, and 11.32 ppm, the NHC imino proton of the NHC ligands appeared in the form of a sharp singlet in the ^1^H NMR (CDCl_3_) spectra for **2a**–**e**.

The lack of signals in the ^1^HNMR spectra that correspond to the NHC imino protons of the palladium complexes **3a**–**e** serves as evidence for complex formation. The NHC imino carbon was identified by the signals in the ^13^C­{^1^H} NMR (CDCl_3_) spectra of ligands **2a**–**e** at 143.2, 142.9, 144.0, 143.1, and 143.2 ppm. In accordance with the literature, the specific palladium complex **3a**–**e** carbene carbon peaks appeared at 163.2, 160.8, 163.8, 163.2, and 163.3 ppm, respectively. In comparison with the corresponding *N*-heterocyclic carbene precursor, which is another sign of complex formation, the carbon carbene resonance on the palladium complex shifted significantly downfield following complexation. The pyridine α-proton peak in the palladium complexes (**3a**–**e**) was observed between 8.99 and 8.91 ppm. These findings line up with the results from various complexes of the same type.^76^ FT-IR, ^1^H NMR, and ^13^C­{^1^H} NMR spectra are presented in detail in the Supporting Information (Figures S1–S30).

### 2.2 X-ray Diffraction Studies

Figures 2 and 3 show the molecular structures of **3b** and **3e** using the atom-numbering scheme, respectively, while Table 2 provides a listing of the significant bond distances and angles. The asymmetric unit of **3b** has two molecules, whereas the asymmetric unit of **3e** includes one molecule. Only one of the two molecules is depicted in Figure 3 regarding purposes of clarity, while parameters for the second molecule are stated in square brackets in the explanation that follows.

**Figure 2:**
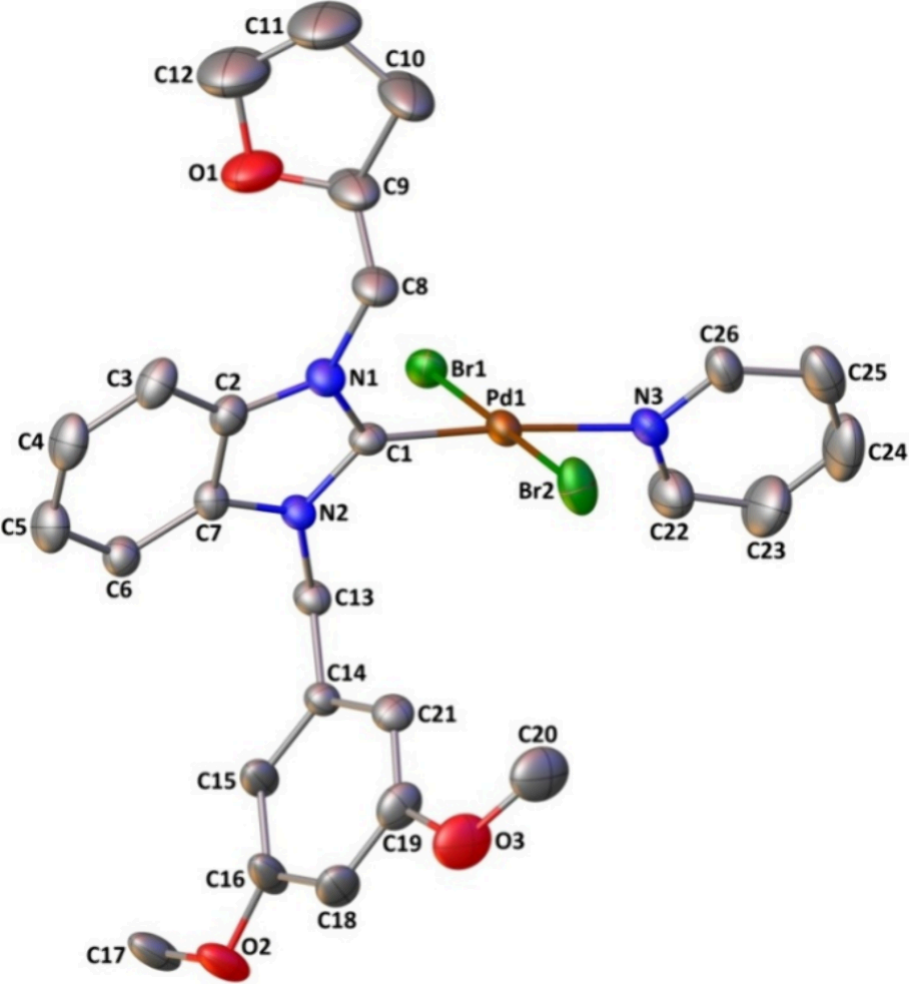
Molecular structure of **3b** shows the atom-labeling scheme. Displacement ellipsoids are drawn at the 20% probability level, and H atoms are omitted for simplicity.

**Figure 3:**
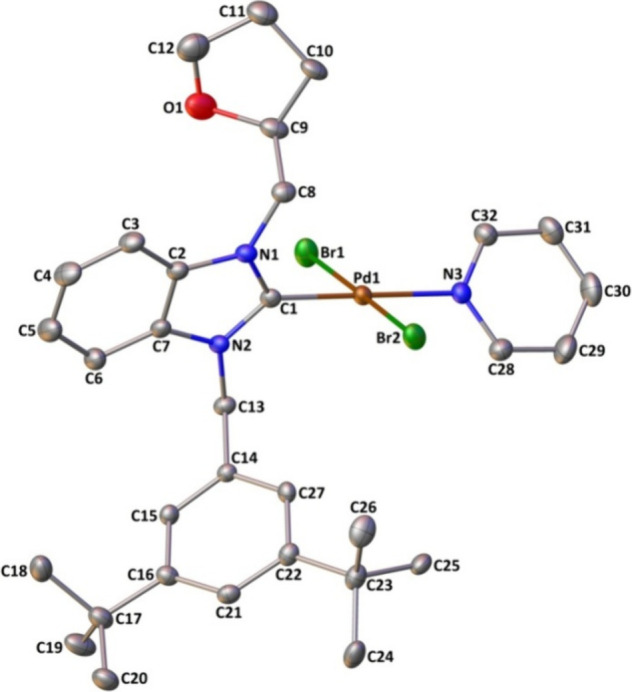
Molecular structure of **3e** shows the atom-labeling scheme. Displacement ellipsoids are drawn at the 20% probability level, and H atoms are omitted for simplicity.

The complexes exhibit a four-coordination arrangement in a square-planar arrangement, surrounded by the carbenic carbon atom of the NHC ligand, the nitrogen atom from the pyridine ring, and two bromide atoms. The four-coordination structural indexes τ_4_ ^77^ and τ_4_^′^ ^78^ are found to be 0.03 and 0.02 for 3e and 0.04 and 0.03 [both 0.03] for 3b, indicating slightly distorted square-planar geometry. In this geometry, the cis angles varying from 88.71(13) to 91.4(2)° and the trans angles changing from 177.66(2) to 179.52(17)° depart somewhat from their ideal values of 90 and 180°, respectively.

The Pd—N and Pd—C bond distances in **3e** are slightly longer than those of **3b** while no remarkable differences are observed in the remaining lengths and they present no unusual features. Eventually, coordination parameters are found to be comparable with what has been observed for complexes of Pd-NHC-pyridine-Br_2_.^79–85^ At the carbene centers, the total internal N—C—N ring angle is approximately ca. 106°. The carbene ring exhibits a nearly perpendicular orientation relative to the coordination plane, characterized by a dihedral angle of 71.94(14)° for **3e** and 72.8(3)°[69.0(3)°] for **3b** (Table 1).

**Table 1:** Selected Distances (Å) and Angles (°) for **3b** and **3e**

**parameters**	**3b**	**3e**	
**Bond distances**			
Pd1—Br1	2.4269(13)	2.4269(7)	2.4310(13)
Pd1—Br2	2.4212(14)	2.4316(7)	2.4265(12)
Pd1—N3	2.084(8)	2.118(4)	2.089(6)
Pd1—C1	1.945(8)	1.963 (4)	1.955(7)
N1—C1	1.360(10)	1.349 (5)	1.353(9)
N2—C1	1.344(9)	1.344 (6)	1.359(9)
**Bond angles**			
Br1—Pd1—Br2	178.70(5)	177.66(2)	178.13(4)
Br1—Pd1—N3	91.4(2)	91.07(11)	91.23(19)
Br2—Pd1—N3	89.9(2)	91.17(11)	90.59(19)
Br1—Pd1—C1	89.7(2)	88.71(13)	89.2(2)
Br2—Pd1—C1	89.1(2)	89.06 (13)	89.0(2)
N3—Pd1—C1	176.1(3)	179.52 (17)	178.0(3)
N1—C1—N2	106.3(7)	106.6 (4)	106.7(6)

### 2.3 C–H Bond Activation of Heteroarenes Using PEPPSI-Pd-NHC Complexes

Ohta et al. demonstrated in 1990 that heterocyclic compounds may be directly arylated using aryl halides by the activation of C–H bonds with good yields.^86^ After that, studies on palladium-catalyzed direct arylation of heteroaromatics have shown a remarkable improvement. Since then, research on the direct arylation of heteroaromatics via palladium catalysis has significantly improved. Then, we used the synthesized Pd-PEPPSI complexes **3a**–**e** to investigate the C–H bond activation of 2-acetylfuran as well as 2-acetythiophen substrates. The cross-coupling reaction between 2-acetylfuran (**4a**) and 4-bromo-acetophenone (**5a**) was selected as the test reaction to be examined using complex **3d** as a catalyst ([Table tbl2]).

**Table 2:**
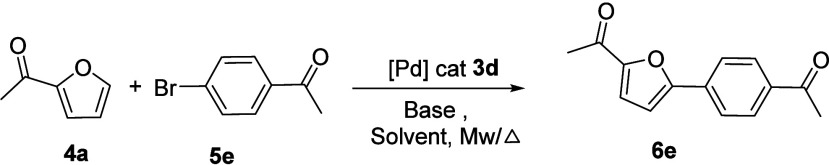
Direct Arylation Process between 2-Acetylfuran and 4-Bromoacetophenone: Optimization

entry^a^	[Pd] cat mol %	solvent	temp [°C]	mode of heating Mw/Δ	time (min)	conversion (proportion of the product^b^ [%]
1	1	DMAc	130	Δ^c^	120	100(52)
2	1	DMAc	130	Mw^d^	12	100(80)
3	1	EtOH	120	Δ	360	
4	1	EtOH	110	Mw	15	25(19)
5	1	isopropanol	100	Δ	360	7(20)
6	1	isopropanol	110	Mw	15	100(23)
7	1	Acetonitrile	120	Δ	360	63(86)
8	1	Acetonitrile	110	Mw	15	12(100)
9	1	water	100	Δ	360	
10	1	water	110	Mw	15	40(50)
11	1	MeOH	110	Mw	15	
12	1	EthylAcetate	110	Mw	15	
13	1	THF	110	Mw	15	
14	1	DMAc	130	Mw	10	99(80)
15	1	DMAc	120	Mw	15	100(84)
16	1	DMAc	120	Mw	12	100(84)
17	1	DMAc	120	Δ	120	100(70)
18	**1**	**DMAc**	**110**	**Mw**	**15**	**100(89)**
19	1	DMAc	100	Mw	30	100(84)
20	1	DMAc	100	Δ	360	52(25)
21	1	DMAc	100	Δ	1440	100(75)
22	1	DMAc	100	Mw	25	100(85)
23	1	DMAc	100	Mw	20	26(92)
24	**0.5**	**DMAc**	**110**	**Mw**	**15**	**100(89)**
25	0.2	DMAc	110	Mw	15	100(75)
26	0.1	DMAc	110	Mw	15	100(69)
27	0.01	DMAc	110	Mw	15	33(80)
28		DMAc	110	Mw	15	
29	1	DMAc	110	Mw	15	80 (7)

a Conditions: 2-acetylfuran (1.2 equiv), 4-bromoacetophenone (1.0 equiv), KOAc (2 equiv), solvent (2 mL).

b Product purity was checked by GC, and conversions were calculated according to aryl bromide with *n*-dodecane as the internal standard.

c Δ: conventional heating.

d Mw: microwave.

Different solvents and bases were used for optimization, and even so, the DMAc/KOAc combination is known to be widely used for direct arylation. To test the efficiency of microwaves, we conducted the reactions using both conventional and microwave heating methods. In the initial series, eight different solvents were examined. According to the results in Table 3, DMAc (Table 2, entry 2) was the best solvent; we achieved 100% conversion with the microwave in just 12 min at 130 °C. A complete conversion takes 120 min when the same solvent is heated conventionally. No product appeared in the case of conventional heating when we used EtOH as a solvent (Table 2, entry 3). Otherwise, the microwave approach provided a 25% rate of conversion (Table 2, entry 4). Since we observed a low conversion of 7% using the conventional heating method, isopropyl alcohol was not the optimal option for the cross-coupling process either (Table 2, entry 5). However, the use of a microwave oven achieved full conversion but with a 23% rate of our desirable product (Table 2, entry 6). Additionally, acetonitrile produced low conversion in both heating procedures (Table 2, entries 7 and 8). When water was utilized as a solvent and heated conventionally, no product was produced (Table 2, entry 9). However, the microwave showed a 40% conversion rate (Table 2, entry 10). No compounds were produced when MeOH, ethyl acetate, and THF were used (Table 2, entries 11, 12, and 13).

**Table 3:** Structure Refinement Parameters and Crystal Data for **3b** and **3e**

**parameters**	**3b**	**3e**
CCDC depository	2426932	2426931
color/shape	yellow/block	yellow/block
chemical formula	[PdBr_2_(C_21_H_24_N_2_O_3_)(C_5_H_5_N)]	[PdBr_2_(C_27_H_36_N_2_O)(C_5_H_5_N)]
formula weight	697.74	749.90
temperature (K)	296(2)	296(2)
wavelength (Å)	0.71073 Mo Kα	0.71073 Mo Kα
crystal system	monoclinic	monoclinic
space group	*P*2_1_/*c* (No. 14)	*C*2/*c* (No. 15)
unit cell parameters		
*a*, *b*, *c* (Å)	2.308(3), 29.261(8), 16.671(5)	14.067(2), 25.257(4), 18.323(3)
α, β, γ (°)	90, 104.109(9), 90	90, 90.786(6), 90
volume (Å^3^)	5823(3)	6509(2)
*Z*	8	8
*D*_calc._(g/cm^3^)	1.592	1.530
μ (mm^–1^)	3.412	3.053
absorption correction	multiscan	multiscan
*T*_min._, *T*_max._	0.3298, 0.7454	0.5461, 0.7456
*F*_000_	2768	3024
crystal size (mm^3^)	0.09 × 0.08 × 0.04	0.12 × 0.11 × 0.09
diffractometer/measurement method	Bruker D8 QUEST/φ and ω scan	Bruker D8 QUEST/φ and ω scan
index ranges	–14 ≤ *h* ≤ 15, –36 ≤ *k* ≤ 36, –20 ≤ *l* ≤ 20	–18 ≤ *h* ≤ 18, –32≤ *k* ≤ 32, –23 ≤ *l* ≤ 23
θ range for data collection (°)	1.392 ≤ θ ≤ 26.574	1.613 ≤ θ ≤ 27.573
reflections collected	112,422	65,991
independent/observed reflections	12,093/6362	7501/5013
*R*_int._	0.0977	0.0644
refinement method	full-matrix least-squares on *F* ^2^	full-matrix least-squares on *F* ^2^
data/restraints/parameters	12,093/131/688	7501/183/407
goodness of fit on *F* ^2^	1.070	1.101
final *R* indices [*I* > 2σ(*I*)]	*R*_1_ = 0.0708, *wR*_2_ = 0.1531	*R*_1_ = 0.0480, *wR*_2_ = 0.1026
*R* indices (all data)	*R*_1_ = 0.1594, *wR*_2_ = 0.2022	*R*_1_ = 0.0921, *wR*_2_ = 0.1270
Δρ_max._, Δρ_min._ (e/Å^3^)	0.80, −0.83	0.61, −0.63

A second set of runs was conducted at varying temperatures and durations. In 15 and 12 min, respectively, a complete conversion was achieved when the temperature dropped from 130 to 120 °C (Table 2, entries 15 and 16). On the other hand, an entire conversion using conventional heating was achieved in 2 h at 120 °C (Table 2, entry 17). Furthermore, we tried to decrease the temperature even further to 100 °C; in the case of conventional heating, a complete conversion was achieved after 24 h (Table 2, entry 21). However, using a microwave set to 100 °C, a full conversion was achieved in just 25 min (Table 2, entry 22). For this reason, we decided to test the reaction at 110 °C, and within 15 min, it generated a 100% conversion rate (Table 2, entry 18). Since our results demonstrated the effectiveness of using a microwave instead of conventional heating, we decided to conduct our investigation using a microwave as a heating source (The principles of green chemistry: energy-efficient design). Although we performed the reaction with an alternative base (KOH), the arylation product was traced only (Table 2, entry 29).

In the last series, we investigated the impact of catalyst loading on the cross-coupling reaction. The arylation of 2-acetylfuran and 4-bromoacetophenone was carried out at 110 °C for 15 min without the inclusion of any Pd catalyst in order to examine the effect of the catalyst on the reaction. However, under these conditions, no product was formed (Table 2, entry 28). To determine the impact of the catalyst on the reaction, 2-acetylfuran was arylated using 4-bromoacetophenone over 15 min and at 110 °C without the inclusion of catalyst. However, no product was created in these circumstances. While decreasing the catalyst’s amount from 1 to 0.5 mol % for 15 min at 110 °C, there was no noticeable improvement on the conversion rate; we obtained good results with total conversion and 89% proportion of the desired product (Table 2, entry 24). Additionally, when we reduced the catalyst loading from 0.5 to 0.2 mol %, we observed a slight decrease in the product proportion, which dropped from 89 to 69%, respectively (Table 2, entries 25 and 26). However, a low conversion of 33% was noted when we utilized 0.001 mol % of catalyst under similar conditions (Table 2, entry 27). Thus, we can conclude that 0.5 mol % is the ideal catalyst loading. We decided to use 0.5 mol % of catalyst loading as it produced the same outcome as 1 mol %, verifying the second green chemistry concept (atom economy).

To determine whether the reaction system operates via a homogeneous or heterogeneous pathway, we conducted a mercury poisoning experiment was conducted. Elemental mercury Hg(0) has long been recognized, over the past nine decades, for its capacity poison heterogeneous metal catalysts through amalgamation or surface adsorption.^87^ As such, this method remains a widely employed diagnostic tool to differentiate between homogeneous and heterogeneous catalysis.

The mercury poisoning experiment was conducted by introducing elemental mercury Hg(0) into the reaction mixture. The inhibition of catalytic activity in the presence of Hg(0) is generally indicative of a heterogeneous catalyst, whereas the absence of such an inhibition suggests a homogeneous catalytic pathway. In this study, Hg(0)-poisoning tests were carried out using complex 3d as the catalyst in the presence of an excess amount of elemental mercury. The results revealed no significant suppression of product formation, thereby indicating that the catalytic process mediated by complex 3d is most likely homogeneous.

Furthermore, the results showed that the microwave heating reduces reaction times from 2 h to 15 min, accelerating C–H activation processes significantly. In fact, microwaves cause instantaneous and uniform temperature increases throughout the reaction medium, avoiding temperature gradients and hot spots typical of conventional heating. This uniformity enhances reaction rates and reduces the energy required to reach the desired temperature and as a result reducing overall energy consumption by up to 60–80% compared to conventional heating.^88^ This makes microwave-assisted C–H functionalization more sustainable and environmentally friendly.^89,90^ On the other hand, the efficient heating can improve catalyst initiation and turnover rates, enabling reactions to proceed at lower temperatures and with less catalyst loading, further saving energy.^91^ Thus, microwave heating enhances energy efficiency in C–H functionalization by rapidly and directly heating reactants volumetrically, reducing reaction times, minimizing heat loss, reducing catalyst loading, and enabling more controlled reaction conditions compared to conventional heating methods.

Following the preliminary research summarized in Table 2, and using the optimal conditions that we have defined for the arylation reaction: base used (AcOK); reaction temperature (110 °C); reaction time (15 min); solvent (DMAc) and Pd-PEPPSI as catalyst (0.5 mol %), we tried to evaluate the scope and limitation of all our palladium complexes Pd-PEPPSI–NHC **3a**–**e** as a catalyst for the direct arylation of 2-acetylfuran and 2-acetylthiophene with different aryl bromides. In fact, a variety of functional groups containing electron-withdrawing groups (EWG) and electron-donating groups (EDG) in the para position of aryl bromide have been used, including aldehyde, acetyl, methyl, methoxy, naphthalene, nitrile, and flour. Under the optimal condition, we tried the arylation reaction of 2-acethylfuran (**4a**) and 2-acethylthiophene (**4b**) with of aryl bromides, including bromobenzene (**5a**), 4-bromotoluene (**5b**), 4-bromoanisole (**5c**), 4-bromobenzaldehyde (**5d**), 4-bromoacetophenone (**5e**), 4-fluorobromobenzene (**5f**), 1-bromonaphtalene (**5g**), and 4-bromobenzonitrile (**5h**). The homocoupling product is the principal byproduct of the reaction. The literature served as inspiration for the gas chromatography conditions.^92^ Figures 4 and 5 present a summary of the results of the direct arylation of 2-acethylfuran and 2-acethylthiophen with aryl bromides, which was catalyzed by complexes **3a**–**e**.

**Figure 4:**
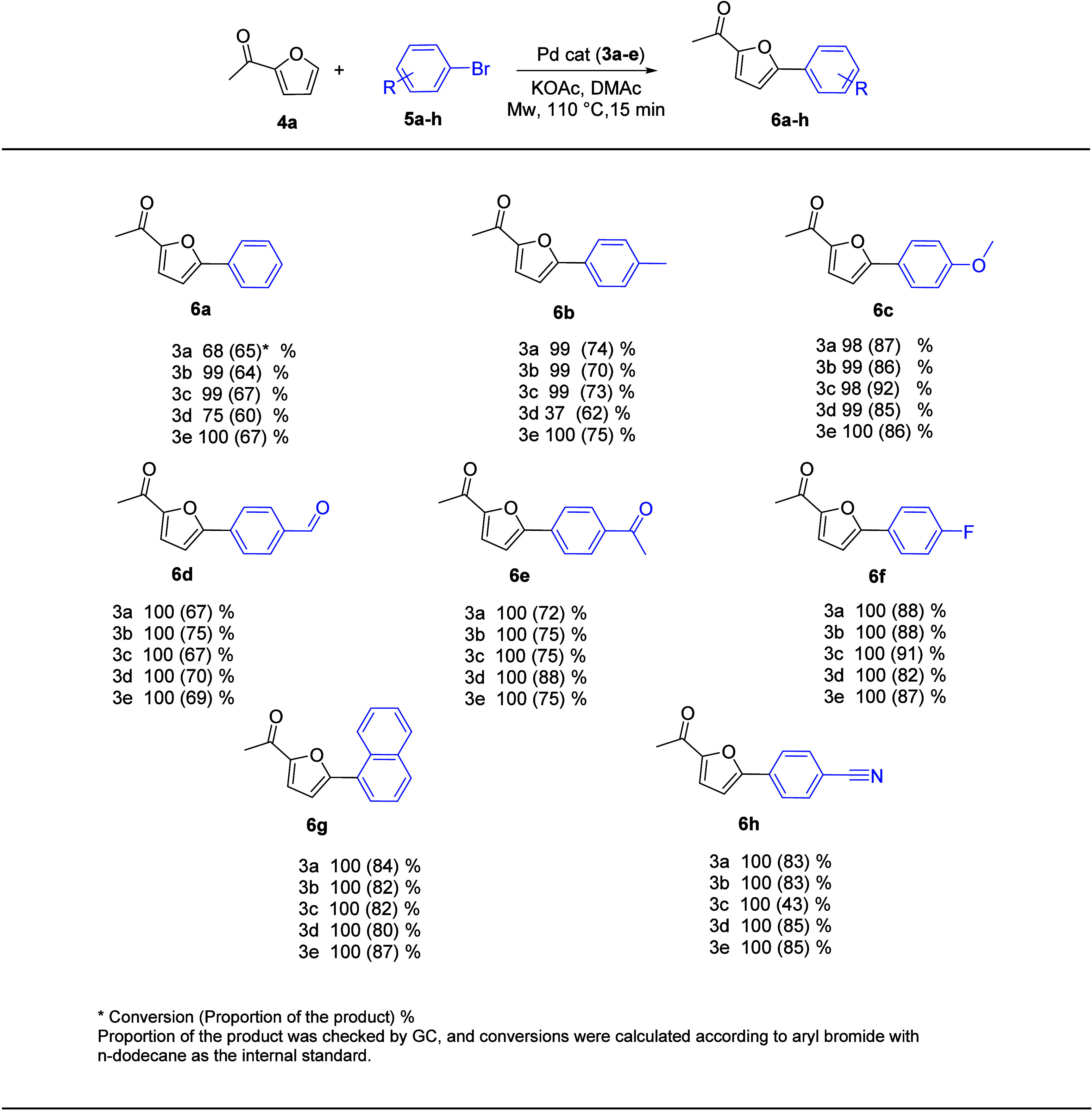
Cross-coupling reactions between the 2-acetylfuran substrate and various aryl bromides.

**Figure 5:**
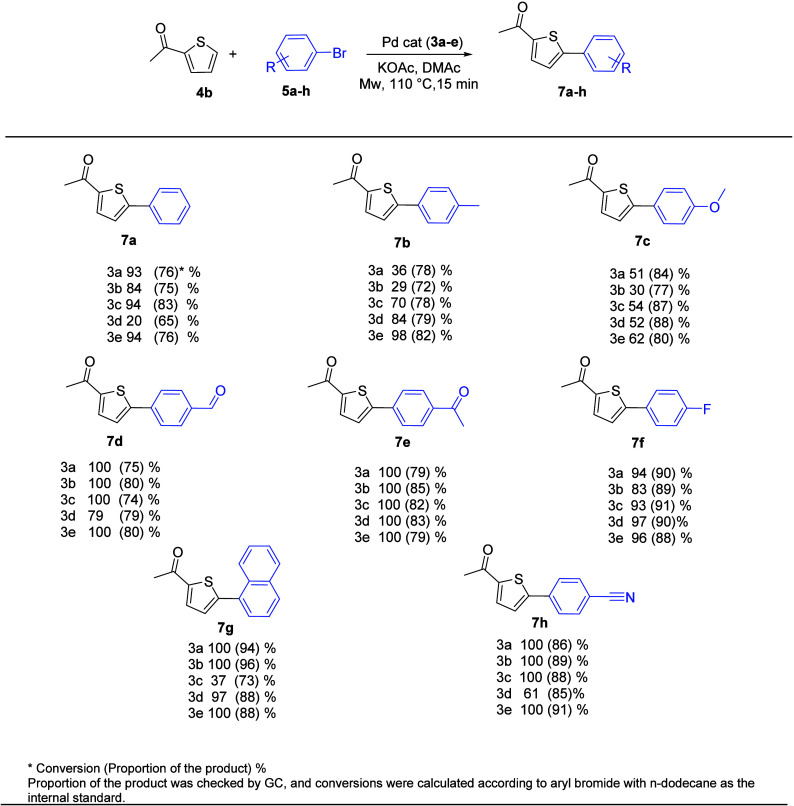
Cross-coupling reactions between the 2-acetylthiophene substrate and various aryl bromides.

Numerous aryl bromides, such as bromobenzene (**5a**), 4-bromotoluene (**5b**), 4-bromoanisole (**5c**), 4-bromobenzaldehyde (**5d**), 4-bromoacetophenone (**5e**), 4-fluorobromobenzene (**5f**), 1-bromonaphtalene (**5g**), and 4-bromobenzonitrile (**5h**), were tested in the cross-coupling reaction of 2-acethyl furan (**4a**) and 2-acethylthiophene (**4b**) under optimum conditions. It should be noted that aryl bromides are more reactive and easier to use in catalytic arylation reactions, while aryl chlorides, despite being less reactive, are attractive due to cost and availability but require advanced ligand design and optimized conditions to achieve comparable catalytic activation and yields. In fact, aryl bromides are generally more reactive than aryl chlorides because the C–Br bond is weaker and more easily undergoes oxidative addition to the metal catalyst (Pd catalyst).^93^ This higher reactivity allows aryl bromides to participate efficiently in direct arylation and cross-coupling reactions under milder conditions and with a broader range of catalysts. However, aryl chlorides are less reactive due to the stronger C–Cl bond and higher barrier for oxidative addition. They typically require specially designed ligands, such as electron-rich and sterically demanding *N*-heterocyclic carbene (NHC) ligands, to facilitate oxidative addition and improve catalytic efficiency.^94^ In addition, we only noticed regioselective monoarylation on the C5-position of 2-acethylfuran (**4a**) and 2-acethylthiophene in our preliminary studies, due to the fact that both heteroarenes’ C5 position hydrogen is more reactive than their C3 and C4 positions. Previous studies showed that the C5 position is more regioselective than C3 and C4 positions.^95,96^ The homocoupling product is the primary consequence of the process. Conversion in the reaction was determined in accordance with the aryl bromide by using GC and dodecane as an internal standard. The ratio of the target product to the homocoupling products was used to calculate selectivity (proportional of the product). We first tested the cross-coupling reaction with 2-acetylfuran using various aryl bromides. The outcomes of **3a**–**e** complex-catalyzed direct C5 arylation of 2-acethylfuran utilizing aryl bromides are summarized in Figure 4.

The most active catalyst was identified by applying the optimal reaction conditions using complexes **3a**–**e**. The measured conversion rates extended from 75 to 97%. Generally, the conversions are quite close to each other. Pd-PEPPSI-NHC complexes exhibit distinct activities due to a minor modification in the NHC ligand’s structure, which is clearly demonstrated by the reaction with the two less activated aryl bromides 4-bromotoluene (**5b**) and 4-bromoanisole (**5c**).

The production of active catalytic intermediates at varying rates is linked to variations in the catalytic activity. Furthermore, the ligand’s steric and electrical characteristics influence its catalytic activity.^97,98^ Complex **3e** showed the highest activity (maximum conversion) compared to those of the other complexes. This is due probably to the bulkier ligand that contains complex **3e**, which could stabilize the active species during the catalytic process.^99^ Moreover, unsymmetrical NHC ligands, as illustrated in the present study, provide a powerful tool to modulate catalytic activation by influencing both the steric and electronic environment of the metal center, leading to improved and sometimes unique catalytic performance in various transformations.^100^ In fact, the different substituents in unsymmetrical NHC ligands generate uneven steric hindrance around the metal, which can direct the substrate approach and binding in a controlled manner. This can accelerate catalytic initiation and improve turnover rates by reducing unfavorable steric clashes in specific orientations. In addition, unsymmetrical ligands can alter the electron-donating ability of the carbene to the metal. Variations in σ-donation and π-acceptance influence the metal’s electronic environment, affecting key steps such as oxidative addition, reductive elimination, or substrate activation.^101^

When 4-bromobenzaldehyde (**5d**), 4-bromoacetophenone (**5e**), 4-fluorobenzene (**5f**), and 4-bromobenzonitrile (**5h**) were used as coupling agents with 2-acethylfuran (**4a**), full conversion and selectivity of products (**6d**), (**6e**), (**6f**), and (**6h**) ranging from 67 to 91% were observed. However, the conversion was slightly decreased with a selectivity that varied from 62 to 92% when 4-bromotoluene (**5b**) and 4-bromoanisol (**5c**) were introduced to the cross-coupling process. Using the synthesized Pd-PEPPSI-NHC complex (**3d**), the cross-coupling process between 2-acethylfuran (**4a**) and 4-bromotoluen (**5b**) exhibited the lowest arylation, with a conversion of 37%. Furthermore, when we employed 4-bromobenzene (**4a**) and 1-bromonaphtalene (**4h**) as aryl bromides in a cross-coupling procedure, good to excellent conversion was measured (68–100%). The products (**6a**) and (**6h**) have selectivity ranging from 60 to 87%.

According to the studies mentioned above, substrates with electron-withdrawing groups often had conversion rates higher than those of substituents with electron-donating groups. This may be explained by the fact that the acceptor substituent activates the halogen mobility during the oxidative addition stage, while the donor substituent decreases it.^102,103^

The arylation process’s scope was further expanded with 2-acethylthiophene using a similar aryl bromide. Figure 5 summarizes the direct arylation of 2-acethylthiophene with aryl bromides, which is catalyzed via PEPPSI-Pd-NHC **3a**–**e**. The results proved that 2-acetylthiophene was also successfully coupled with aryl bromides. Actually, when the 2-acethylthiophene (**4b**) substrate was reacted with aryl bromides (**5a**–**h**) using Pd-PEPPSI-NHC complexes **3a**–**e** as catalysts, moderate to good conversion was achieved (20–100%).

Moderate to good reactivity was obtained when the 2-acetylthiophen (**4b**) substrate was reacted with bromobenzene (**5a**) and 1-bromonaphtalene (**5g**), with conversions in the range of 20 to 100% measured. While 4-bromobenzaldehyde (**5d**), 4-bromoacetophenone (**5e**), 4-bromofluorobenzene (**5f**), and 4-bromobenzonitrile (**5h**) were used as partners with the heteroarene (**4b**), high conversion with good selectivity was reported in the range of 74 to 90%. Both conversion and the selectivity of products (**7b**) and (**7e**) were moderately decreased in the case of coupling with 4-bromotoluen (**5b**) and 4-bromoanisol (**5c**). The conversion rate ranged from 26 to 98%. According to the results, 2-acethylfuran appears to yield the most product as compared to 2-acethyfurane.

Several researchers have previously reported the direct arylation of heteroarenes through C–H activation, which is catalyzed by Pd-PEPPSI-NHC. Şahin and colleagues^104^ previously reported utilizing Pd-PEPPSI complexes as a catalyst to arylate 2-acethylfuran and 2-acethylthiophene with various aryl bromides. The results demonstrated that conversions between 10 and 21% were obtained via the cross-coupling reaction of electron-rich halides, such as 4-bromoanisol and 4-bromotoluene, with both heteroarenes. Nevertheless, our results showed that a moderate to good conversion varied from 30 to 100% using both heteroarenes, even with the electro-rich aryl bromides (4-bromoanisol and 4-bromotoluene). Touj and colleagues^105^ did, however, achieve a satisfactory conversion using 4-bromoanisol and 4-bromotoluene in 2024. Under a temperature of 150 °C, over 2 h and with a catalyst loading of 1 mol %, a maximum of 98% conversion was achieved. Özdemir's group^106^ achieved similar results by employing a reaction temperature of 120 °C, a reaction period of 2 to 4 h, and catalyst loading up to 1 mol %. These conditions appeared to be harsh. Similar heteroarenes have often been used in these earlier studies using higher catalyst loadings (1–5 mol %). In addition, for the direct arylation, a longer reaction time (1–4 h) and a higher reaction temperature (120 – 150 °C) have been selected. Furthermore, similar procedures have been used in the majority of published investigations, including microwave irradiation, commercial catalysts, long-term reactions, and high temperatures.^107–109^

To the best of our knowledge, this work is the first example of direct arylation of heteroarenes using synthesized Pd-PEEPSI-NHC complexes as catalysts operating under microwave irradiation. The current study permits lowering the catalyst loading to 0.5 mol %, the temperature over 110 °C, and the reaction for 15 min. Furthermore, heteroarenes were arylated effectively, and the results were sufficient when compared to similar research that has already been published.

## 3 Conclusions

Biaryl scaffolds are found in several medicinal products and herbal medicines. Five PEPPSI-Pd-NHC complexes were synthesized and reported in this study. Utilizing microwave irradiation, the novel complexes were employed as a catalyst to create C–C bonds through C–H bond activation of 2-acethylfuran and 2-acethylthiophene with a wide variety of aryl bromides. In 15 min at 110 °C and with 0.5 mol % catalyst loading, the arylation reaction successfully progressed under the fixed conditions according to this study leading to moderate to high yields. Additionally, this catalytic system performed well with all generated compounds and produced satisfactory outcomes. Furthermore, it was demonstrated that the catalytic performance was influenced by NHC ligands having sterically bulky backbones. However, certain issues, such as the use of more benign solvent systems, still have to be addressed in the future. Our studies also aim to extend the scope of this transformation to include other heteroarene derivatives.

## 4 Experimental Section

### 4.1 Materials and Methods

Standard Schlenk line procedures were used for all manipulations under an argon environment. The Sigma-Aldrich Co. (Poole, Dorset, UK) was the supplier of the chemicals and solvents. An Electrothermal 9200 melting point equipment was used to measure the melting points in open capillary tubes. The PerkinElmer Spectrum 100 Spectrophotometer was used to obtain infrared spectra on an ATR unit within the 400–4000 cm^–1^ range.

^1^H NMR and ^13^C NMR spectra were recorded using Bruker Avance AMX and Bruker Avance III spectrometers operating at 400 MHz (^1^H NMR) and at 100 MHz (^13^C NMR) in CDCl_3_ (deuterated chloroform) and DMSO-*d*_6_ (dimethyl sulfoxide-d6) with the addition of tetramethylsilane (TMS). NMR multiplicities are abbreviated as follows: s, singlet; d, doublet; t, triplet; hept, heptet; m, multiplet signal. The chemical shifts (d) are reported in ppm. Coupling constants (*J* values) are given in hertz (Hz). High-resolution mass spectrometric (HRMS) analyses were performed on an Agilent 6530B LC Q-TOF instrument employing an electrospray ionization (ESI) technique. The GC (Varian 3900, fitted with a WCOT fused-silica column (25 m × 0.25 mm) was utilized to analyze the catalytic processes, which were conducted in a CEM microwave Discover system.

### 4.2 General Procedure for Preparation of Benzimidazolium Salts **2a**–**e**

The benzimidaolium salts **2a**–**e** were synthesized by reacting the resultant N-substituted benzimidazole (1 mmol), and various aryl halides were dissolved (1.1 mmol) in *N*,*N*-dimethylformamide (DMF). During 48 h, the resultant mixture was heated at 80 °C. Thin layer chromatography (TLC) was used to assess the reaction. To precipitate the product, 30 mL of diethyl ether was added to the reaction mixture once the reaction was complete. Following the filtration process, the resulting white solid was vacuum-dried before being recrystallized from a 1:3 solution of DCM and diethyl ether solvents for enhanced purification.

#### 4.2.1 3-(3,5-Dimethylbenzyl)-1-((tetrahydrofuran-2-yl)­methyl)-1*H*-benzimidazolium Bromide (**2a**)

Yield: 80%; M.p = 185.6 °C; FT-IR, ν­(CN) (cm^–1^) = 1585 ; ^1^ H NMR (CDCl_3_,400 MHz) δ (ppm): 1.72–1.81 (m, 1H, H5′); 1.88–1.92 (m, 2H, H4′); 2.25 (s, 6H, Ha,b CH3 × 2); 2.57–2.59 (m, 1H, H5′); 3.69–3.75 (m, 1 H, H3′); 3.90–3.95 (m, 1 H, H3′); 4.37–4.39 (m, 1 H, H2′); 4.60–4.66 (m, 1H, H1′); 4.86–4.90 (m, 1H, H1′); 5.69 (s, 2H, H1″); 7.02 (s, 2H, H3″, 7″, arom,); 6.93 (s, 1H, H5″, arom); 7.48–7.58 (m, 3H, H5, 6, 7, arom); 7.84 (d, 1H, H4, arom, *J* = 8.1 Hz); 11. Twenty-one (s, 1H, H2, NCHN). ^13^C NMR (CDCl_3_, 100 MHz) (δ (ppm)): 21.2 (Ca,b, CH3 × 2), 25.7 (C4′); 29.1 (C5′); 51.6 (C1′); 51.7 (C1″); 68.7 (C3′); 77.3 (C2′); 113.5 (C4, arom); 114.2 (C7, arom); 125.9 (C5, 6, arom); 127.1 (C3″, 7″, arom); 130.9 (C5″, arom); 131.0 (C9, arom); 132.1 (C8, arom); 132.3 (C2″, arom); 139.1 (C4″, 6″, arom); 143.2 (C2, NCHN).

#### 4.2.2 3-(3,5-Dimethoxybenzyl)-1-((tetrahydrofuran-2-yl)­methyl)-1*H*-benzimidazolium Bromide (**2b**)

Yield: 76%; M.p = 165.3 °C; FT-IR, ν­(CN) (cm^–1^) = 1576 ; ^1^H NMR (DMSO-*d*_6_,400 MHz) δ (ppm): 1.63–1.69 (m, 1H, H5′); 1.879–1.986 (m, 2H, H4′); 2.05–2.13 (m, 1H, H5′); 3.63–3.68 (m, 1 H, H3′); 3.72 (s, 6H, Ha,b OCH3 × 2); 3.78–3.83 (m, 1 H, H3′); 4.25–4.31 (m, 1 H, H2′); 4.25–4. 31 (m, 1H, H1′); 4.50–4.56 (m, 1H, H1′); 5.73 (s, 2H, H1″); 6.70 (s, 2H, H3″, 7″, arom,); 6.49 (s, 1H, H5″, arom); 7.68 (t, 2H, H5, 6, arom); 8.01 (d, 1H, H4, arom, *J* = 8.9 Hz); 8.14 (d, 1H, H4, arom, *J* = 8.5 Hz); 10. 01 (s, 1H, H2, NCHN). ^13^C NMR (DMSO-*d*_6_, 100 MHz) (δ (ppm)): 25.7 (C4′); 28.2 (C5′); 49.8 (C1′); 50.2 (C1″); 55.3 (Ca,b, OCH3 × 2), 67.6 (C3′); 75.8 (C2′);99.9 (C5″, arom); 106.4 (C4, 7, arom); 113.8 (C5, arom); 114.2 (C6, arom); 126.7 (C3″, 7″, arom); 130.6 (C9, arom); 131.6 (C8, arom); 136.1 (C2″, arom); 142.9 (C2, NCHN); 160.8 (C4″, 6″, arom).

#### 4.2.3 1-((Tetrahydrofuran-2-yl)­methyl)-3-(3,4,5-trimethoxybenzyl)-1*H*-benzimidazolium Chloride (**2c**)

Yield: 78%; M.p = 205.6 °C; FT-IR, ν­(CN) (cm^–1^) = 1575 ; ^1^ H NMR (CDCl_3_,400 MHz) δ (ppm): 1.73–1.77 (m, 1H, H5′); 1.87–1.90 (m, 2H, H4′); 2.19–2.23 (m, 1H, H5′); 3.67–3.72 (m, 1 H, H3′); 3.77 (s, 3H, Hc, OCH3); 3.83 (s, 6H, Ha,b, (OCH3)­2); 3.87–3.93 (m, 1 H, H3′); 4.30–4.41 (m, 1 H, H2′); 4.55–4. 60 (m, 1H, H1′); 4.78–4.82 (m, 1H, H1′); 5.72 (s, 2H, H1″); 6.81 (s, 2H, H3″, 7″, arom,); 7.52 (t, 2H, H5, 6, arom, *J* = 6.8); 7.64 (d, 1H, H7, arom, *J* = 7.3 Hz); 7.79 (d, 1H, H4, arom, *J* = 7.5 Hz); 11.64 (s, 1H, H2, NCHN). ^13^C NMR (CDCl_3_, 100 MHz) (δ (ppm)): 25.7 (C4′); 29.1 (C5′); 51.6 (C1′); 51.7 (C1″); 56.7 (Ca,b, OCH3 × 2); 60.8 (*Cc*,OCH3); 68.6 (C3′); 76.9 (C2′); 106.0 (C4,7, arom); 113.4 (C5, arom); 114.1 (C6, arom); 127.0 (C3″, arom); 127.0 (C7′′, arom); 128.3 (C5″, arom); 131.0 (C9, arom); 132.1 (C8, arom); 138.6 (C2″, arom); 144.0 (C2, NCHN); 153.8 (C4″, 6″, arom).

#### 4.2.4 3-(4-(*tert*-Butyl)­benzyl)-1-((tetrahydrofuran-2-yl)­methyl)-1*H*-benzimidazolium Bromide (**2d**)

Yield: 80%; M.p = 215.1 °C; FT-IR, ν­(CN)­(cm^–1^) = 1584 ; ^1^H NMR (CDCl_3_,400 MHz) δ (ppm): 1.23 (s, 9H, Ha, CH3 × 3); 1.73–1.80 (m, 1H, H5′); 1.88–1.91 (m, 2H, H4′); 2.19–2.26 (m, 1H, H5′); 3.68–3.74 (m, 1 H, H3′); 3.89–3.94 (m, 1 H, H3′); 4.33–4.39 (m, 1 H, H2′); 4.58–4.64 (m, 1H, H1′); 4.84–4.88 (m, 1H, H1′); 5.75 (s, 2H, H1″); 7.133 (d, 2H, H3″, 4″, arom, *J* = 8.2 Hz); 7.40 (d, 2H, H6″,7″, arom, *J* = 8.3 Hz); 7.50–7.61 (m, 3H, H5, 6, 7, arom); 7.83 (d, 1H, H4, arom, *J* = 8.0 Hz); 11.34 (s, 1H, H2, NCHN). ^13^C NMR (CDCl_3_, 100 MHz) (δ (ppm)): 25.6 (C4′); 29.0 (C5′); 31.2 (Ca, CH_3_×3); 34.7 (C *tert*-Butyl); 51.1 (C1′); 51.5 (C1″); 68.7 (C3′); 76.9 (C2′); 113.5 (C4, arom); 114.1 (C7, arom); 126.9 (C5, 6, arom); 127.0 (C3″, arom); 127.1 (C4″, arom); 128.1 (C6″, C7″, arom); 129.6 (C9, arom); 131.0 (C8, arom); 132.1 (C2″, arom); 143.1 (C2, NCHN); 152.4 (C5″, arom).

#### 4.2.5 3-(3,5-Di-*tert*-butylbenzyl)-1-((tetrahydrofuran-2-yl)­methyl)-1*H*-benzimidazolium Bromide (**2e**)

Yield: 83%; M.p = 219.2 °C; FT-IR, ν­(CN) (cm^–1^) = 1577 ; ^1^H NMR (CDCl_3_,400 MHz) δ (ppm): 1.24 (s, 18H, Ha,b CH3 × 6); 1.71–1.78 (m, 1H, H5′); 1.86–1.90 (m, 2H, H4′); 2.19–2.25 (m, 1H, H5′); 3.66–3.71 (m, 1 H, H3′); 3.86–3.92 (m, 1 H, H3′); 4.34–4.36 (m, 1 H, H2′); 4.64–4.69 (m, 1H, H1′); 4.84–4.87 (m, 1H, H1′); 5.72 (dd, 2H, H1″, *J* = 34.2, 15.1); 7.23 (s, 2H, H3″, 7″, arom,); 7.36 (s, 1H, H5″, arom); 7.49–7.58 (m, 3H, H5, 6, 7, arom); 7.85 (d, 1H, H4, arom, *J* = 8.9 Hz); 11.321 (s, 1H, H2, NCHN). ^13^C NMR (CDCl_3_, 100 MHz) (δ (ppm)): 25.7 (C4′); 28.9 (C5′); 31.2 (Ca,b, CH_3_×6); 34.9 (C *tert*-Butyl × 2); 51.4 (C1′); 51.0 (C1″); 68.6 (C3′); 76.6 (C2′); 113.4 (C4, arom); 114.1 (C7, arom); 122.4 (C5, 6, arom); 123.24 (C3″, arom); 126.99 (C5″, arom); 127.06 (C7″, arom); 131.07 (C9, arom); 131.77 (C8, arom); 132.0 (C2″, arom); 143.2 (C2, NCHN); 152.1 (C4″, 6″, arom).

### 4.3 General Procedure for Preparation of PEPPSI-Pd-NHC Complexes **3a**–**e**

A solution of benzimidalium salts (**2a**–**e**), K_2_CO_3_ (5.0 mmol), pyridine (1.01 mmol), KBr (10.0 mmol), and PdCl_2_ (1.05 mmol) in acetonitrile was heated at 65 °C and stirred for 16 h. After that, a Celite was used to filter the reaction mixture, and the solvent was removed under reduced pressure. The yellow Pd-PEPPSI-NHC complex was obtained via the chromatographic purification of the crude solid. Bright-yellow crystals of Pd-PEPPSI-NHC complexes (**3a**–**e**) were produced by crystallizing the crude product from 1:3 solution of DCM/hexane.

#### 4.3.1 Dibromo­[3-(3,5-dimethylbenzyl)-1-((tetrahydrofuran-2-yl)­methyl)-1*H*-benzimidazole2-ylidene]­pyridine Palladium­(II) (**3a**)

Yield: 58%; m.p = 192.3 °C; FT-IR, ν­(CN) (cm^–1^) = 1542 ; ^1^H NMR (CDCl_3_,400 MHz) δ (ppm): 1.94–2.04 (m, 3H, H5′, H4′); 2.28 (s, 6H, Ha,b CH3 × 2); 2.31–2.34 (m, 1H, H5′); 3.79–3.84 (m, 1 H, H3′); 3.97–4.02 (m, 1 H, H3′); 4.60–4.65 (m, 1 H, H2′); 4.88–4.92 (m, 1H, H1′); 5.34–5.38 (m, 1H, H1′); 6.05 (dd, 2H, H1″, *J* = 15.7 Hz, *J* = 49.2 Hz); 6.94 (s, 1H, H5″, arom); 7.07–7.13 (m, 2H, H5, 6, arom); 7.22–7.24 (m, 3H, H3″, 7″, 7, arom); 7.32 (t, 2H, H3‴,5‴, pyri, *J* = 5.8); 7.62 (d, 1H, H4, arom, *J* = 8.2 Hz); 7.76 (t, 1H, H4‴, pyri, *J* = 7.6); 9.03 (d, 2H, H 2‴, 6‴, pyri, *J* = 4.9). ^13^C NMR (CDCl_3_, 100 MHz) (δ (ppm)): 21.3 (Ca,b, CH3 × 2), 25.8 (C4′); 30.0 (C5′); 53.4 (C1′); 53.6 (C1″); 68.5 (C3′); 78.5 (C2′); 111.3 (C4, arom); 112.0 (C7, arom); 123.1 (C5, arom); 123.1 (C6, arom); 124.6 (C3″, 7″, arom); 125.9 (C3‴, 5‴, pyri); 129.8 (C5″, arom); 134.5 (C9, arom); 134.9 (C8, arom); 135.9 (C2″, arom); 138.0 (C4‴, pyri); 138.9 (C4″, 6″, arom); 163.2 (C2, NCHN). MS (ESI) *m*/*z*: calcd for C_26_H_30_N_3_OPdBr_2_ [M + H]^+^: 665.97645, found: 665.98407; calcd for C_26_H_31_N_2_OPdBr [M+2H–Br]^+^: 586.06744, found: 586.0506; calcd for C_21_H_24_N_3_OPd [M-Py-2Br–H]^+^: 425.08452, found: 425.08714.

#### 4.3.2 Dibromo­[3-(3,5-dimethoxybenzyl)-1-((tetrahydrofuran-2-yl)­methyl)-1*H*-benzimidazole2-ylidene]­pyridine Palladium­(II) (**3b**)

Yield: 61%; m.p = 219.3 °C; FT-IR, ν­(CN) (cm^–1^) = 1511; ^1^ H NMR (DMSO-*d*_6_,400 MHz) δ (ppm): 1.91–2.03 (m, 3H, H4′,5′); 2.26–2.31 (m, 1H, H5′); 3.78–3.83 (m, 1 H, H3′); 3.74 (s, 6H, Ha,b OCH3 × 2); 3.93–4.01 (m, 1 H, H3′); 4.60–4.66 (m, 1 H, H2′); 4.85–4. 92 (m, 1H, H1′); 5.31–5.36 (m, 1H, H1′); 6.00 (dd, 2H, H1″, *J* = 53.3 Hz, *J* = 15.7 Hz); 6.40 (s, 1H, H5″, arom); 6.79 (s, 2H, H3″, 7″, arom); 7.09–7.15 (m, 2H, H5, 6, arom); 7.21–7.25 (m, 1H, H4, arom); 7.35 (t, 2H, H3‴, 5‴, *J* = 6.5 Hz, pyri); 7.61 (d, 1H, H4, *J* = 8.2 Hz arom); 7.75 (t, 1H, H4‴, *J* = 7.6 Hz, pyri); 9.04 (d, 2H, H_2,6_, *J* = 4.9). ^13^C NMR (DMSO-*d*_6_, 100 MHz) (δ (ppm)): 25.8 (C4′); 30.0 (C5′); 53.4 (C1′); 54.0 (C1″); 55.8 (Ca,b, OCH_3_×2), 68.4 (C3′); 78.4 (C2′); 100.6 (C5″, arom); 106.1 (C3″, 7″, arom); 111.3 (C4, arom); 112.0 (C7, arom); 123.2 (C5, arom); 123.2 (C6, arom); 124.7 (C3‴, 5‴, pyri); 134.4 (C9, arom); 135.9 (C8, arom); 137.5 (C2″, arom); 138.1 (C4‴, pyri); 152.77 (C2‴, 6‴, pyri); 161.3 (C4″, 6″, arom); 163.9 (C2, NCHN); 160.8 (C4″, 6″, arom). MS (ESI) *m*/*z*: calcd for C_26_H_30_N_3_O_3_PdBr_2_ [M + H]^+^: 697.97286, found: calcd for C_26_H_31_N_3_O_3_PdBr_2_ [M+2H–Br]^+^: 618.04397, found: 618.05727; calcd for C_26_H_32_N_3_O_3_Pd [M+2H-2Br]^+^: 539.13948, found: 539.00239.

#### 4.3.3 Dichloro­[1-((tetrahydrofuran-2-yl)­methyl)-3-(3,4,5-trimethoxybenzyl)-1*H*-benzimidazole-2-ylidene]­pyridine Palladium­(II) (**3c**)

Yield: 64%; m.p = 222.3 °C; FT-IR, ν­(CN) (cm^–1^) = 1528 ; ^1^ H NMR (CDCl_3_,400 MHz) δ (ppm): 1.94–2.05 (m, 1H, H4′, H5′); 2.25–2.32 (m, 1H, H5′); 3.676–3.80 (m, 1 H, H3′); 3.80 (s, 3H, Hc, OCH3); 3.82 (s, 6H, Ha,b, (OCH3)­2); 3.93–3.99 (m, 1 H, H3′); 4.69–4.75 (m, 1 H, H2′); 4.79–4.84 (m, 1H, H1′); 5.31–5.36 (m, 1H, H1′); 6.04 (dd, 2H, H1″, *J* = 54.4 Hz, *J* = 15.6 Hz); 6.87 (s, 2H, H3″, 7″, arom,); 7.13–7.19 (m, 2H, H5, 6, arom); 7.64 (m, 1H, H7, arom); 7.35 (t, 2H, H3‴, 5‴, *J* = 7.6 Hz, pyri); 7.62 (d, 1H, H4, arom, *J* = 8.2 Hz); 8.99 (d, 2H, H2‴, 6‴, *J* = 5.0, pyri). ^13^C NMR (CDC_l3_, 100 MHz) (δ (ppm)): 25.8 (C4′); 29.7 (C5′); 52.9 (C1′); 53.4 (C1″); 56.6 (Ca,b, OCH_3_×2); 60.9 (*Cc*,OCH_3_); 68.4 (C3′); 78.6 (C2′); 105.2 (C3″, C7″, arom); 111.2 (C4,7, arom); 111.3 (C4,7, arom); 123.4 (C5, arom); 123.4 (C6, arom); 124.7 (C3‴, 5‴, pyri); 130.9 (C9, arom); 134.1 (C8, arom); 135.7 (C2″, arom); 138.3 (C4‴, pyri); 151.3 (C4″, 5″, 6″, arom); 153.7 (C2‴, 6‴, pyri), 163.8 (C2, NCHN). MS (ESI) *m*/*z*: calcd for C_27_H_31_N_3_O_4_O_4_PdCl [M-Cl]^+^: 302.10379, found: 602.10808; calcd for C_22_H_25_N_2_O_4_ [M-Pd-Py-2Cl-2H]^+^: 381.18143, found: 381.18360.

#### 4.3.4 Dibromo­[3-(4-(*tert*-butyl)­benzyl)-1-((tetrahydrofuran-2-yl)­methyl)-1*H*-benzimidazole2-ylidene]­pyridine Palladium­(II) (**3d**)

Yield: 67%; m.p = 204.2 °C; FT-IR, ν­(CN) (cm^–1^) = 1536 ; ^1^H NMR (CDCl_3_,400 MHz) δ (ppm): 1.29 (s, 9H, Ha, CH3 × 3); 1.95–2.02 (m, 3H, H4′, 5′); 2.127–2.32 (m, 1H, H5′); 3.78–3.88 (m, 1 H, H3′); 3.96–4.02 (m, 1 H, H3′); 4.61–4.66 (m, 1 H, H2′); 4.86–4.92 (m, 1H, H1′); 5.32–4.37 (m, 1H, H1′); 6.12 (dd, 2H, H1″, *J* = 37.6 Hz, *J* = 15.7 Hz); 7.04 – 7.12 (m, 2H, H3″, 4″, arom); 7.22 (t, 1H, H3‴, pyri, *J* = 8.3); 7.33–7.38 (m, 4H, H5‴, 5, 6, 7, pyri, arom); 7.52 (d, 2H, H 6″, 7″, arom, *J* = 8.3 Hz); 7.61 (d, 1H, H4, arom, *J* = 8.2 Hz); 7.74 (t, 1H, H4‴, pyri, *J* = 7.6 Hz); 9.03 (d, 2H, H2‴, 6‴, pyri, *J* = 4.9 Hz). ^13^C NMR (CDCl_3_, 100 MHz) (δ (ppm)): 25.8 (C4′); 30.8 (C5′); 31.2 (Ca, CH3 × 3); 34.7 (C *tert*-Butyl); 53.4 (C1′); 53.5 (C1″); 68.4 (C3′); 78.4 (C2′); 111.4 (C4, arom); 112.0 (C7, arom); 123.1 (C5, 6, arom); 124.7 (C3‴, 5‴, pyri); 125.8 (C4″, 6″, arom); 127.9 (C3″, C7″, arom); 132.0 (C4‴, pyri); 134.4 (C9, arom); 136.0 (C8, arom); 138.0 (C2″, arom); 151.1 (C5″, arom); 152.7 (C2‴, 6‴, pyri); 163.2 (C2, NCHN). MS (ESI) *m*/*z*: calcd for C_28_H_31_N_3_OPdBr_2_ [M + H]^+^: 694.00775, found: 694.01299; calcd for C_28_H_32_N_3_OPdBr [M+2H–Br]^+^: 614.09928, found: 614.08684; calcd for C_23_H_27_N_2_OPd [M-Py-2Br–H]^+^: 353.11582, found: 353.11917.

#### 4.3.5 Dibromo­[3-(3,5-di-*tert*-butylbenzyl)-1-((tetrahydrofuran-2-yl)­methyl)-1*H*-benzimidazole2-ylidene]­pyridine Palladium­(II) (**3e**)

Yield: 64%; m.p = 246.5 °C; FT-IR, ν­(CN)­(cm^–1^) = 1498 ; ^1^H NMR (CDCl_3_,400 MHz) δ (ppm): 1.29 (s, 18H, Ha,b CH3 × 6); 1.95–2.03 (m, 3H, H5′, H4′); 2.27–2.33 (m, 1H, H5′); 3.79–3.84 (m, 1 H, H3′); 3.96–4.02 (m, 1 H, H3′); 4.63–4.69 (m, 1 H, H2′); 4.89–4.93 (m, 1H, H1′); 5.32–5.37 (m, 1H, H1′); 6.07 (dd, 2H, H1″, *J* = 96.9, 15.7); 7.01 (d, 1H, H7, *J* = 8.0, arom); 7.08 (t, 2H, H5, arom, *J* = 7.7 Hz); 7.19 (t, 1H, H6, arom, *J* = 7.7 Hz); 7.32–7.39 (m, 3H, H3‴, 5‴, 5″, pyri, arom); 7.39 (s, 2H, H3″, 7″, arom); 7.61 (d, 1H, H4, arom, *J* = 8.2 Hz); 7.74 (t, 1H, H4‴, pyri, *J* = 7.7 Hz); 9.05 (d, 2H, H2‴, 6‴, pyri, *J* = 4.9 Hz). ^13^C NMR (CDCl_3_, 100 MHz) (δ (ppm)): 25.8 (C4′); 30.0 (C5′); 31.6 (Ca,b, CH_3_×6); 35.1 (C *tert*-Butyl × 2); 53.4 (C1′); 54.6 (C1″); 68.4 (C3′); 78.5 (C2′); 121.9 (C3‴, 5‴, pyri); 122.5 (C4, C7, arom); 123.0 (C5, 6, arom); 124.6 (C3″, 7″, 5″, arom); 134.0 (C9, arom); 134.5 (C8, arom); 135.9 (C2″, arom); 138.0 (C4‴, pyri); 151.4 (C2‴, 6‴, pyri); 152.8 (C4″, 6″, arom); 163.3 (C2, NCHN). MS (ESI) *m*/*z*: calcd for C_32_H_42_N_3_OPdBr_2_ [M + H]^+^: 750.07035, found: 750.07458; calcd for C_28_H_33_N_3_OPdBr [M+2H–Br]^+^: 670.16189, found: 670.14982; calcd for C_28_H_33_N_3_OPd [M+2H-2Br]^+^: 591.24355, found: 591.10723.

### 4.4 General Procedure for the Arylation of 2-Acethylfuran and 2-Acethylthiophene

Under open-air conditions, heteroarenes (2-acethylfuran and 2-acethylthiophene) (1.2 mmol), aryl bromides (1.0 mmol), KOAc (2 mmol), Pd-PEPPSI-NHC complexes **3a**–**e**, and *N*,*N*-dimethylacetamide (DMAc) (2 mL) as solvents were added to a small tube. Next, the tube was inserted into the microwave reactor and heated under the required conditions. After the desired time, 2 mL of dichloromethane (DCM) was added into the tube, and the final solution was passed through a layer of Celite. The conversion and yield of pure biaryl compounds were calculated by GC relative to the aryl bromide using dodecane as the internal standard.

### 4.5 Mercury Poisoning Experiment

2-Acetylfuran (1.2 mmol), 4-bromobenazldeyde (1.0 mmol), KOAc (2.0 mmol), and DMAc (2 mL) were added to a Schlenk tube under an argon atmosphere. Subsequently, the Pd-PEPPSI-NHC catalyst **3d** (0.5 mol %) was added to the stirred solution in the Schlenk tube, and one drop of Hg was added with a syringe to the reaction mixture. The closed Schlenk tube was stirred at 110 °C for 15 min (microwave heating). At the end of the reaction, dichloromethane (2 mL) was added to the crude mixture. The solution was filtered through a pad of Celite to remove the solid particles and then used for GC analysis. The yields were calculated according to aryl bromide by GC analysis.

### 4.6 X-ray Crystal Structure Analysis

Using graphite-monochromated Mo Kα radiation and the φ and ω scan methods, intensity measurements were obtained at 296(2) K on a Bruker D8 QUEST diffractometer. APEX2 ^110^ was used for data collecting, and SAINT^110^ was used for cell refinement and data reduction. SHELXT-2018 ^111^ was used to solve the structures using a dual-space algorithm, and SHELXL-2019 ^112^ was used to refine them via full-matrix least-squares calculations on *F* ^2^. A riding model was used to treat all hydrogen atoms once they were placed in idealized positions. The bond lengths for aromatic CH, methine CH, CH_2_, and CH_3_ atoms were fixed at 0.93, 0.98, 0.97, and 0.96 Å, respectively. *U*_iso_(*H*) = 1.2*U*eq (1.5*U*eq for CH3) was the fixed displacement parameter for the H atoms. The atoms of methyl in **3e** and methoxy groups in **3b** exhibit positional disorder and refined using SIMU, DELU, SADI, and DFIX restraints.^78^ Information about crystal data, data collection, and structure refining is compiled in Table 3. Molecular graphics were generated utilizing OLEX2.^113^

## Supplementary Material







## Notes

Crystallographic data **3b** (CIF)
Crystallographic data **3e** (CIF)
Spectra and data (^1^H, ^13^C, FT-IR, and Mass) of compound http://www.ccdc.cam.ac.uk/structures­(PDF)

## Notes

CCDC 2426931 and 2426932 contain the supplementary crystallographic data for the compounds reported in this article. These data can be obtained free of charge from The Cambridge Crystallographic Data Centre via www.ccdc.cam.ac.uk/structures
