# Supplementary material for: An Eco-friendly Approach to C–H Bond Activation through Microwave Irradiation Employing Synthesized Palladium-PEPPSI-NHC Complexes
Source: ACS Omega. 2025 Aug 27;10(35):39994–40008. doi: 10.1021/acsomega.5c04713 (PMC12423799; doi:10.1021/acsomega.5c04713)
Supplement: Supplementary file 3 [file ao5c04713_si_003.pdf]

Supporting Information

An eco-friendly approach to C-H bond activation through microwave irradiation employing synthesized palladium-PEPPSI-NHC complexes

Ichraf Slimani [1], İsmail Özdemir [1,2], Nevin Gürbüz [1,2], Bülent Alıcı [1,2], Nahide Burcu Arslan[3], Namık Özdemir[4]

[1] Catalysis Research and Application Center, Inönü University, 44280 Malatya, Turkey I [2]Faculty of Science and Arts, Department of Chemistry, Inönü University, 44280 Malatya, Turkey I[3]Department of Physics, Faculty of Science and Arts, Giresun University, 28100 Giresun, Turkey, Türkiye I [4] Department of Physics, Faculty of Science, Ondokuz Mayıs University, 55139 Samsun, Türkiye

Correspondence: Prof. İsmail Özdemir (ismail.ozdemir@inonu.edu.tr)

Table of figures

Figure S. 1 ^{1}H NMR spectrum of salt 2a ($CDCl_3$, 400 MHz)....3
Figure S. 2 ^{13}C NMR spectrum of salt 2a ($CDCl_3$, 100 MHz)....3
Figure S. 3 ^{1}H NMR spectrum of salt 2b (CDMSOd_6, 400 MHz)....4
Figure S. 4 ^{13}C NMR spectrum of salt 2b (DMSOd_6, 100 MHz)....4
Figure S. 5 ^{1}H NMR spectrum of salt 2c ($CDCl_3$, 400 MHz)....5
Figure S. 6 ^{13}C NMR spectrum of salt 2c ($CDCl_3$, 100 MHz)....5
Figure S. 7 ^{1}H NMR spectrum of salt 2d ($CDCl_3$, 400 MHz)....6
Figure S. 8 ^{13}C NMR spectrum of salt 2d ($CDCl_3$, 100 MHz)....6
Figure S. 9 ^{1}H NMR spectrum of salt 2e ($CDCl_3$, 400 MHz)....7
Figure S. 10 ^{13}C NMR spectrum of salt 2e ($CDCl_3$, 100 MHz)....7
Figure S. 11 ^{1}H NMR spectrum of complex 3a ($CDCl_3$, 400 MHz)....8
Figure S. 12 ^{13}C NMR spectrum of complex 3a ($CDCl_3$, 100 MHz)....8
Figure S. 13 ^{1}H NMR spectrum of complex 3b ($CDCl_3$, 400 MHz)....9
Figure S. 14 ^{13}C NMR spectrum of complex 3b ($CDCl_3$, 100 MHz)....9
Figure S. 15 ^{1}H NMR spectrum of complex 3c ($CDCl_3$, 400 MHz)....10
Figure S. 16 ^{13}C NMR spectrum of complex 3c ($CDCl_3$, 100 MHz)....10
Figure S. 17 ^{1}H NMR spectrum of complex 3d ($CDCl_3$, 400 MHz)....11
Figure S. 18 ^{13}C NMR spectrum of complex 3d ($CDCl_3$, 100 MHz)....11
Figure S. 19 ^{1}H NMR spectrum of complex 3e ($CDCl_3$, 400 MHz)....12
Figure S. 20 ^{13}C NMR spectrum of complex 3e ($CDCl_3$, 100 MHz)....12
Figure S. 21 FT-IR spectrum of salt 2a....13
Figure S. 22 FT-IR spectrum of salt 2b....13
Figure S. 23 FT-IR spectrum of salt 2c....14
Figure S. 24 FT-IR spectrum of salt 2d....14
Figure S. 25 FT-IR spectrum of salt 2e....15
Figure S. 26 FT-IR spectrum of complex 3a....15
Figure S. 27 FT-IR spectrum of complex 3b....16
Figure S. 28 FT-IR spectrum of complex 3c....16
Figure S. 29 FT-IR spectrum of complex 3d....17
Figure S. 30 FT-IR spectrum of complex 3e....17
Figure S. 31 HRMS spectrum of complex 3a....189
Figure S. 33 HRMS spectrum of complex 3c....20
Figure S. 34 HRMS spectrum of complex 3d....21
Figure S. 35 HRMS spectrum of complex 3e....23
Figure S. 37 ^{13}C NMR spectrum of product 6c ($CDCl_3$, 100 MHz)....23
Figure S. 38 ^{1}H NMR spectrum of product 6e ($CDCl_3$, 400 MHz)....24
Figure S. 39 ^{13}C NMR spectrum of product 6e ($CDCl_3$, 100 MHz)....24

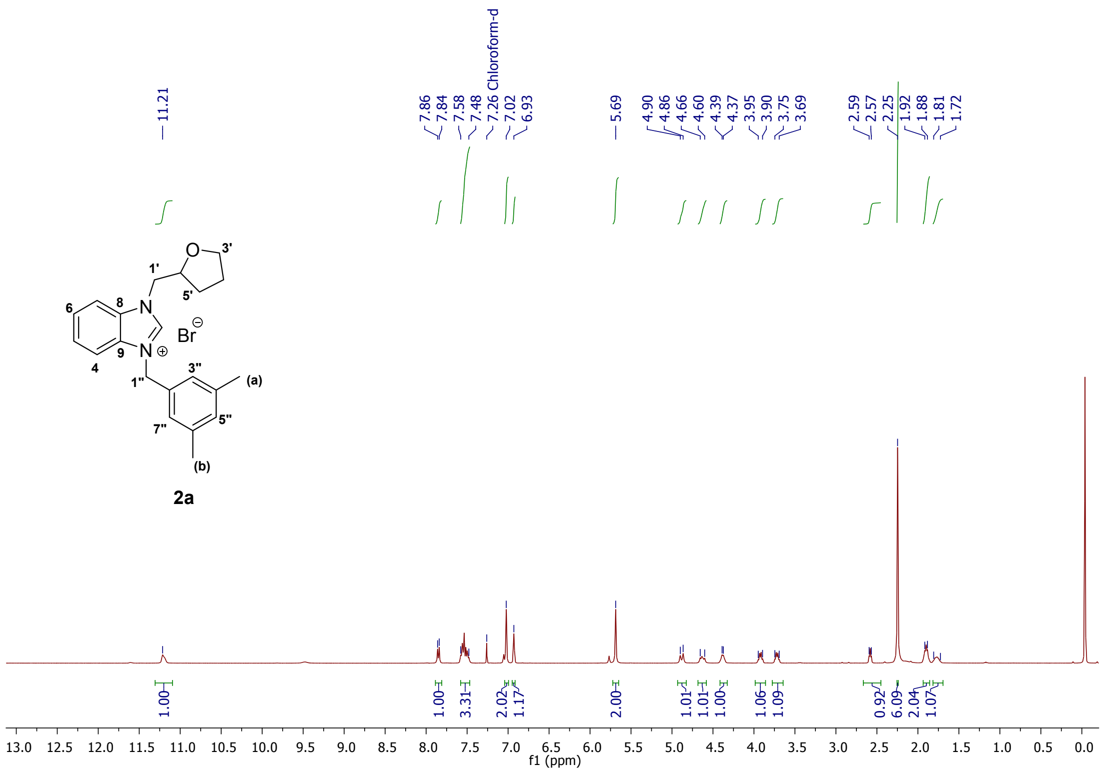

Figure S. 1 ^{1}H NMR spectrum of salt 2a ($CDCl_3$, 400 MHz)

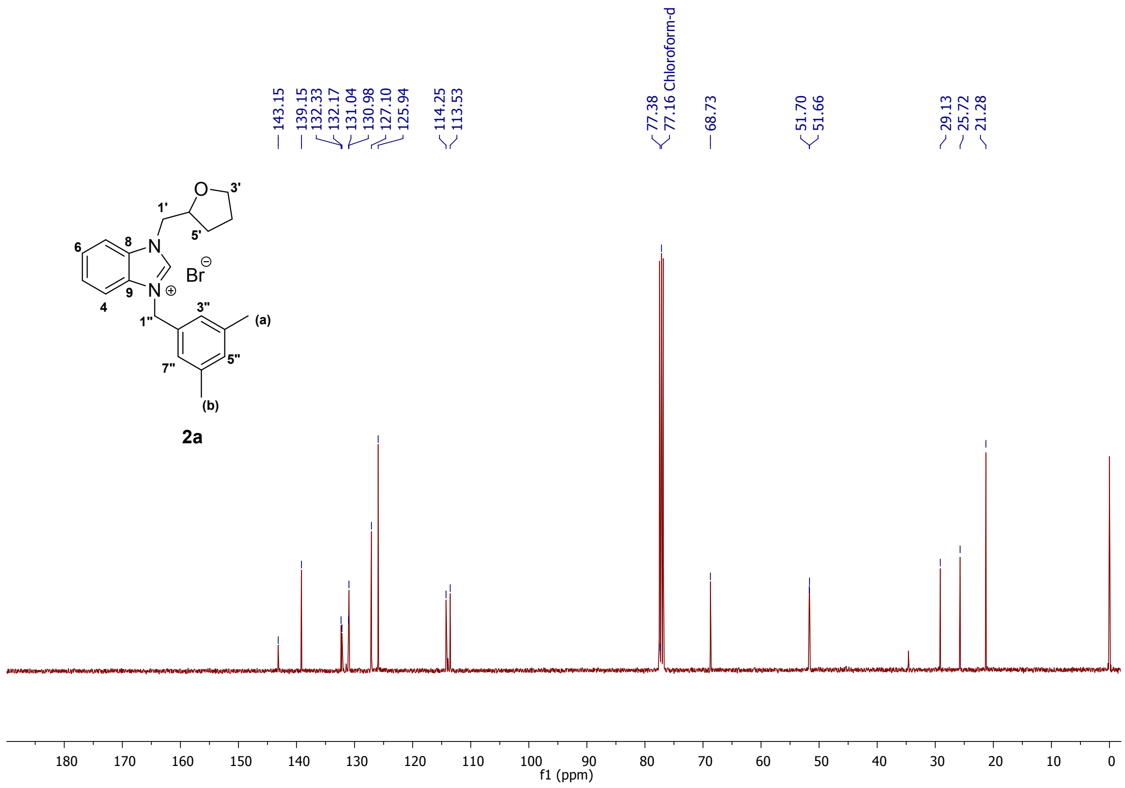

Figure S. 2 ^{13}C NMR spectrum of salt 2a ($CDCl_3$, 100 MHz)

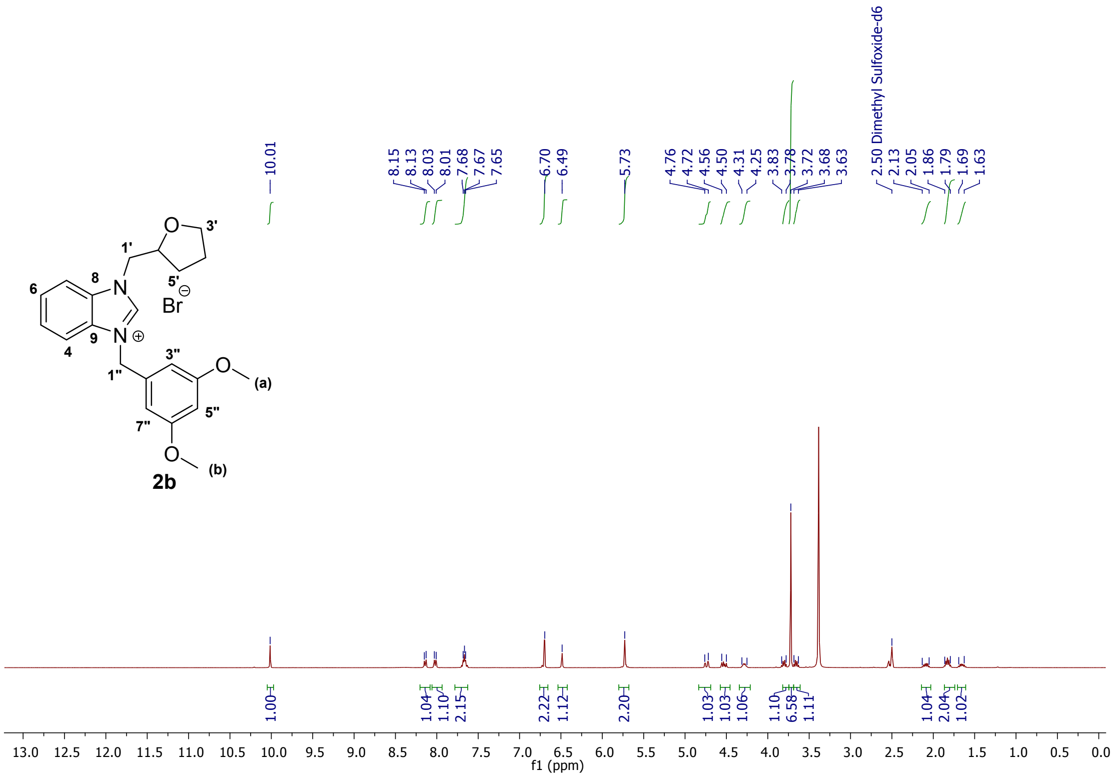

Figure S. 3 ^{1}H NMR spectrum of salt 2b (CDMSOd_6, 400 MHz)

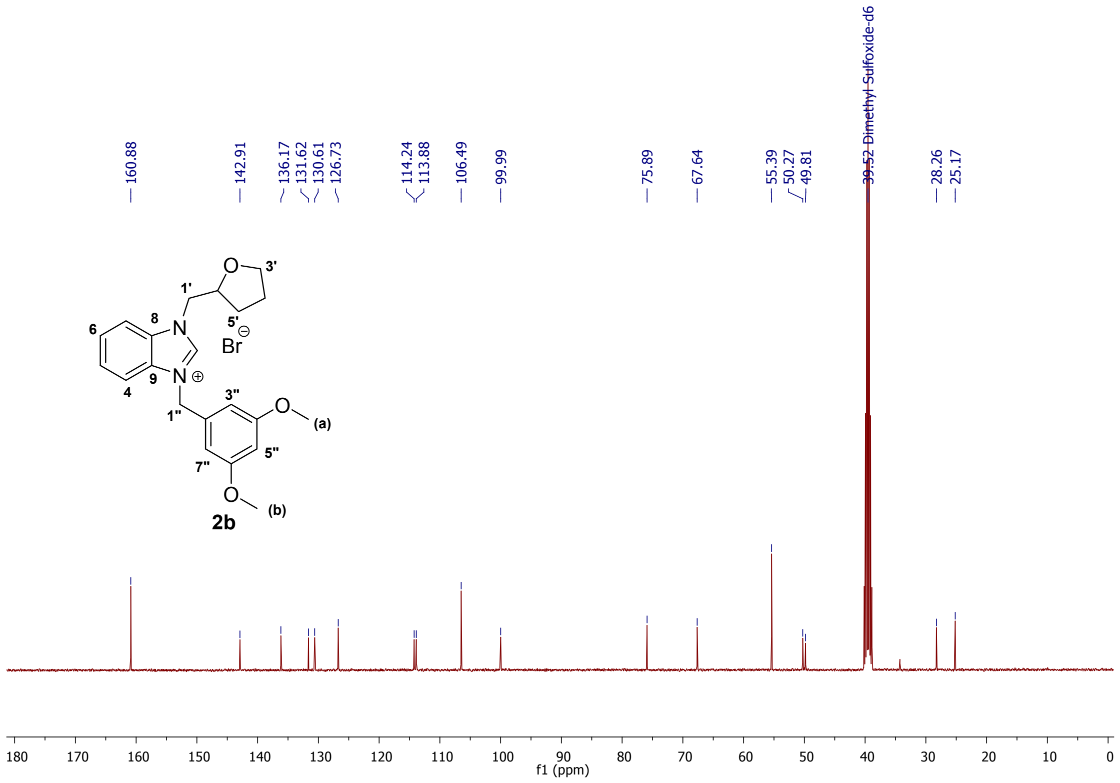

Figure S. 4 ^{13}C NMR spectrum of salt 2b (DMSOd_6, 100 MHz)

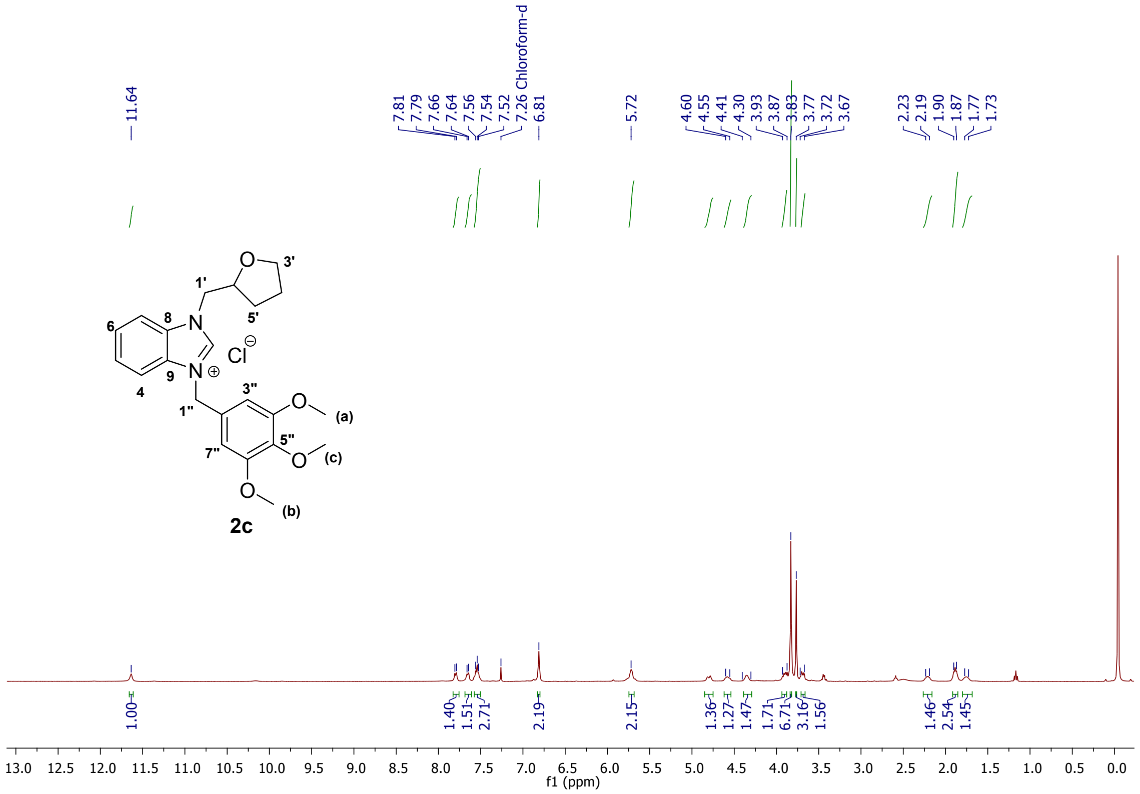

Figure S. 5 ^{1}H NMR spectrum of salt 2c ($CDCl_3$, 400 MHz)

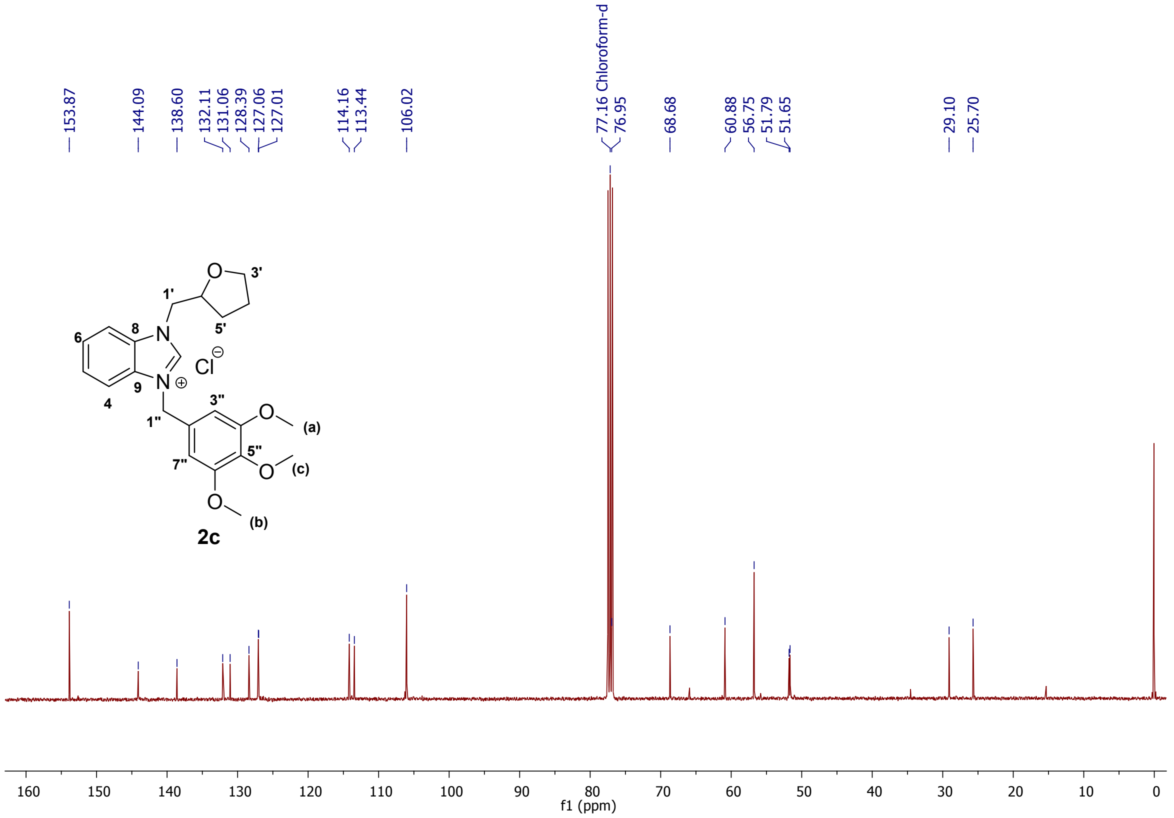

Figure S. 6 ^{13}C NMR spectrum of salt 2c ($CDCl_3$, 100 MHz)

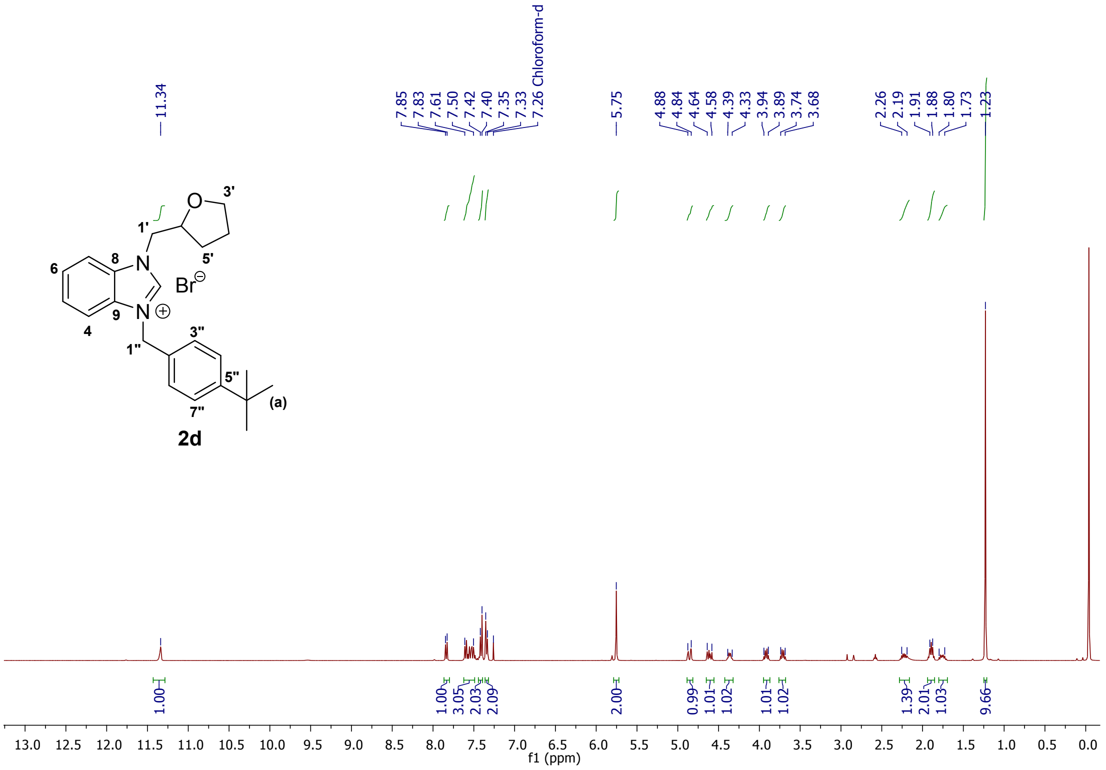

Figure S. 7 ^{1}H NMR spectrum of salt 2d ($CDCl_3$, 400 MHz)

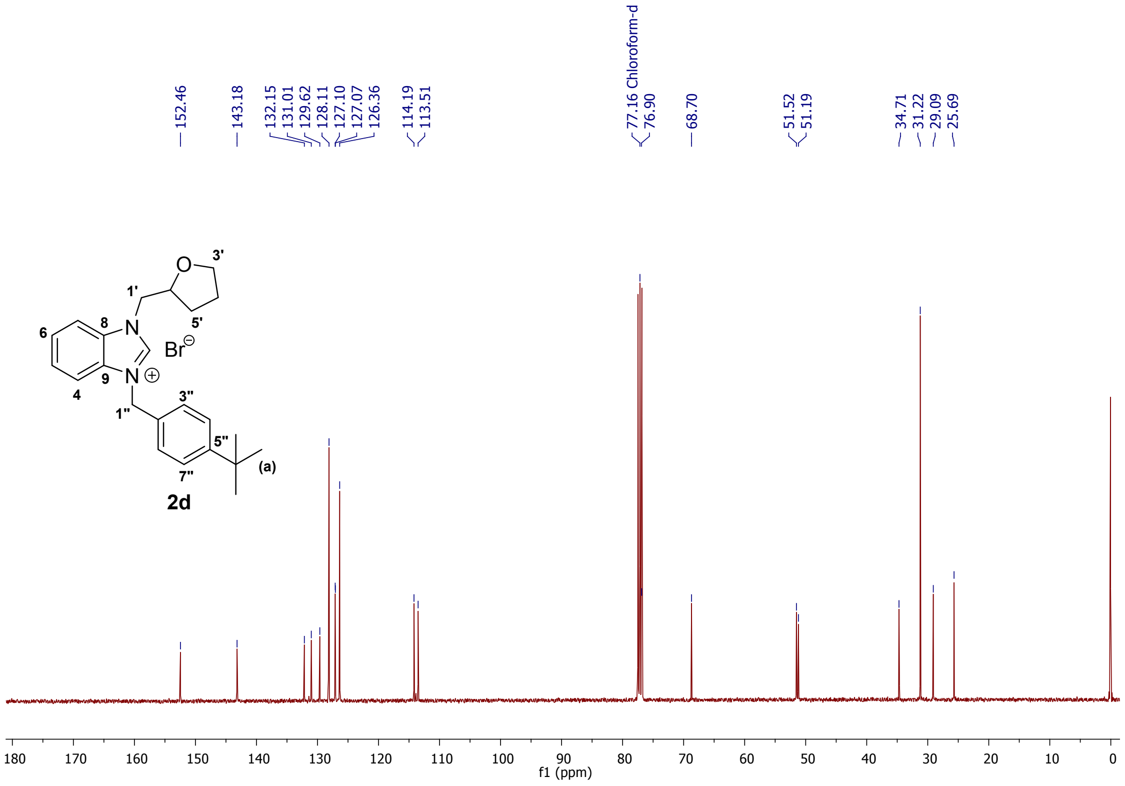

Figure S. 8 ^{13}C NMR spectrum of salt 2d ($CDCl_3$, 100 MHz)

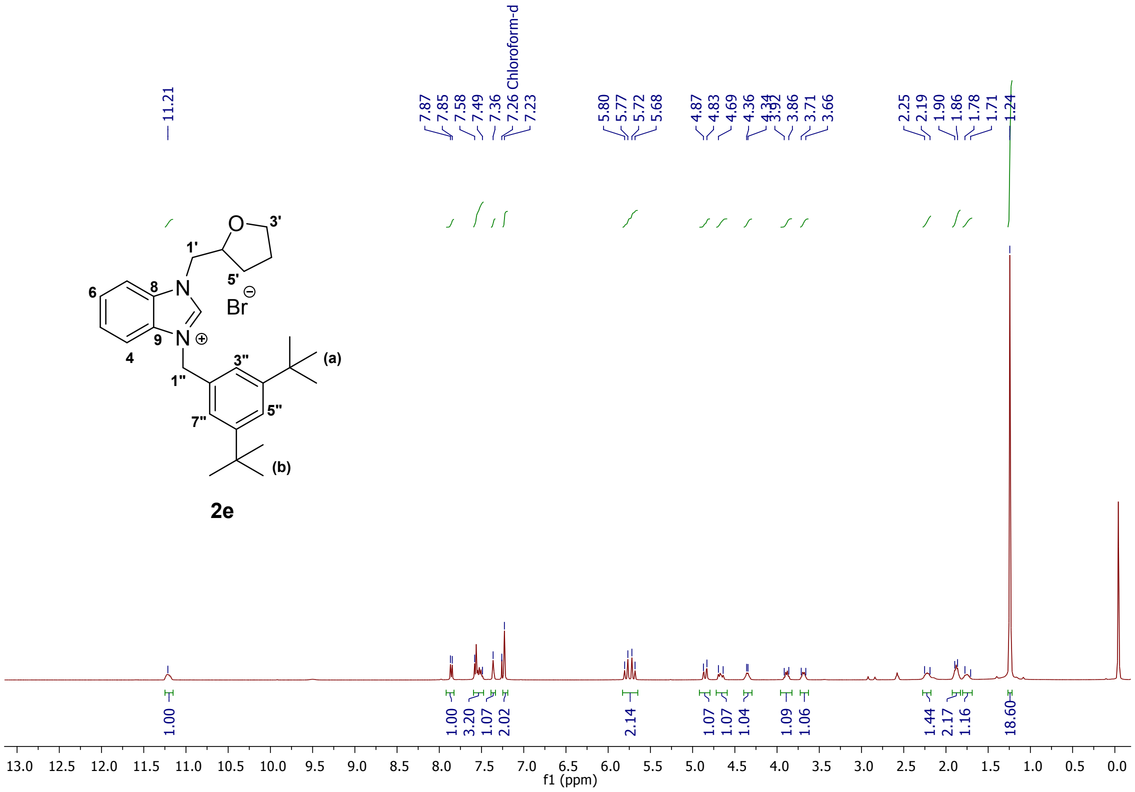

Figure S. 9 ^{1}H NMR spectrum of salt 2e ($CDCl_3$, 400 MHz)

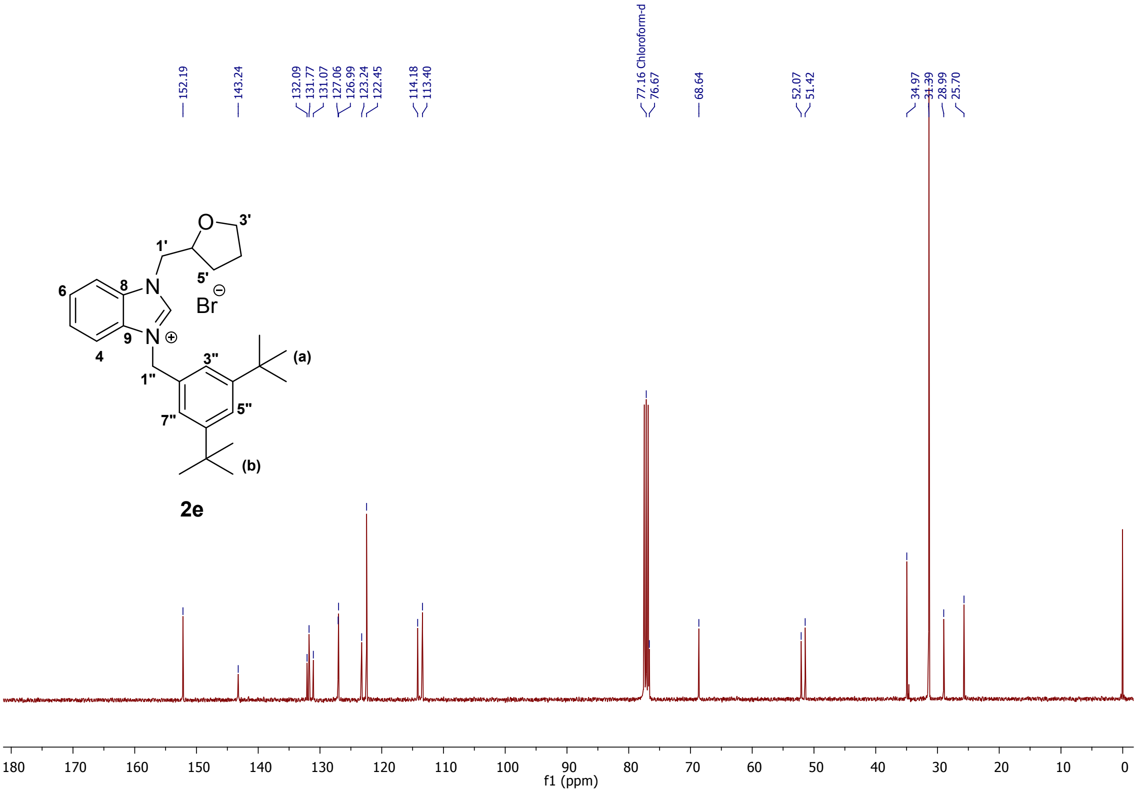

Figure S. 10 ^{13}C NMR spectrum of salt 2e ($CDCl_3$, 100 MHz)

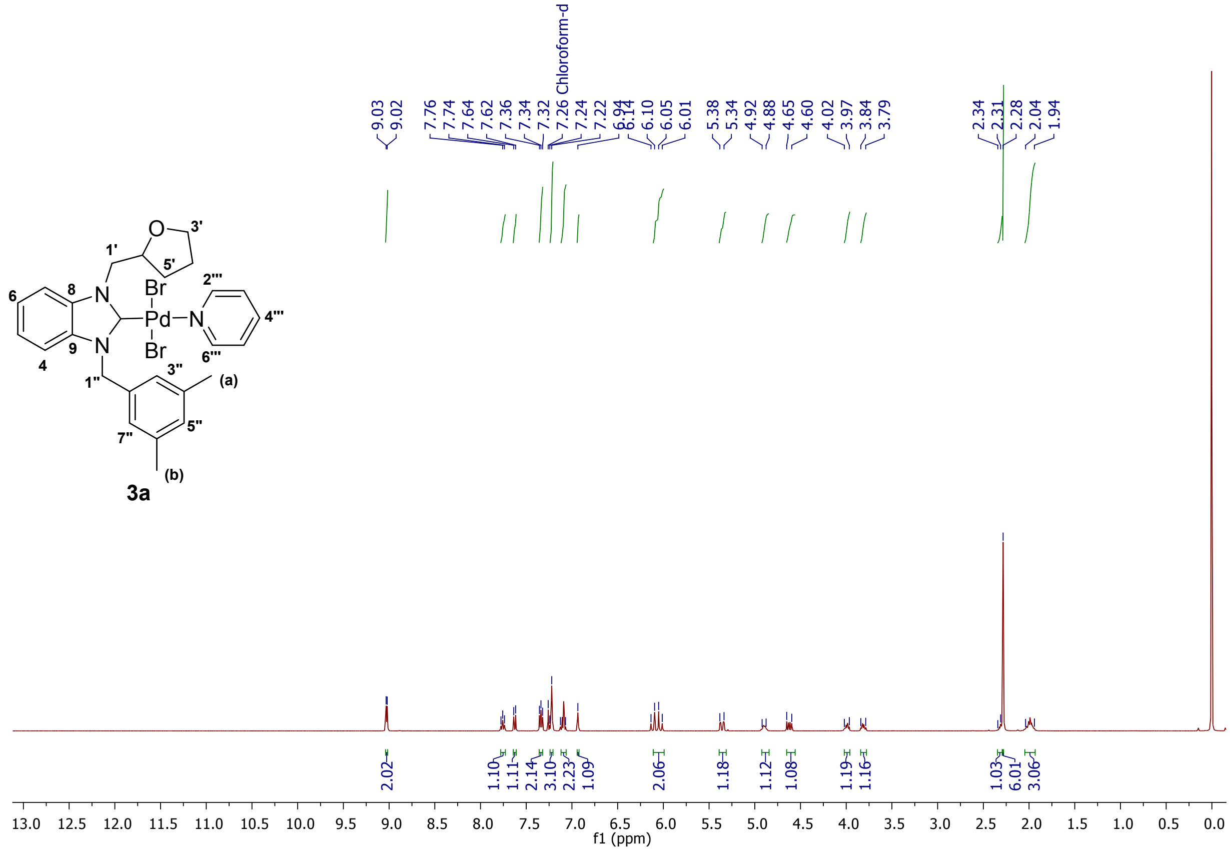

Figure S. 11 ^{1}H NMR spectrum of complex 3a ($CDCl_3$, 400 MHz)

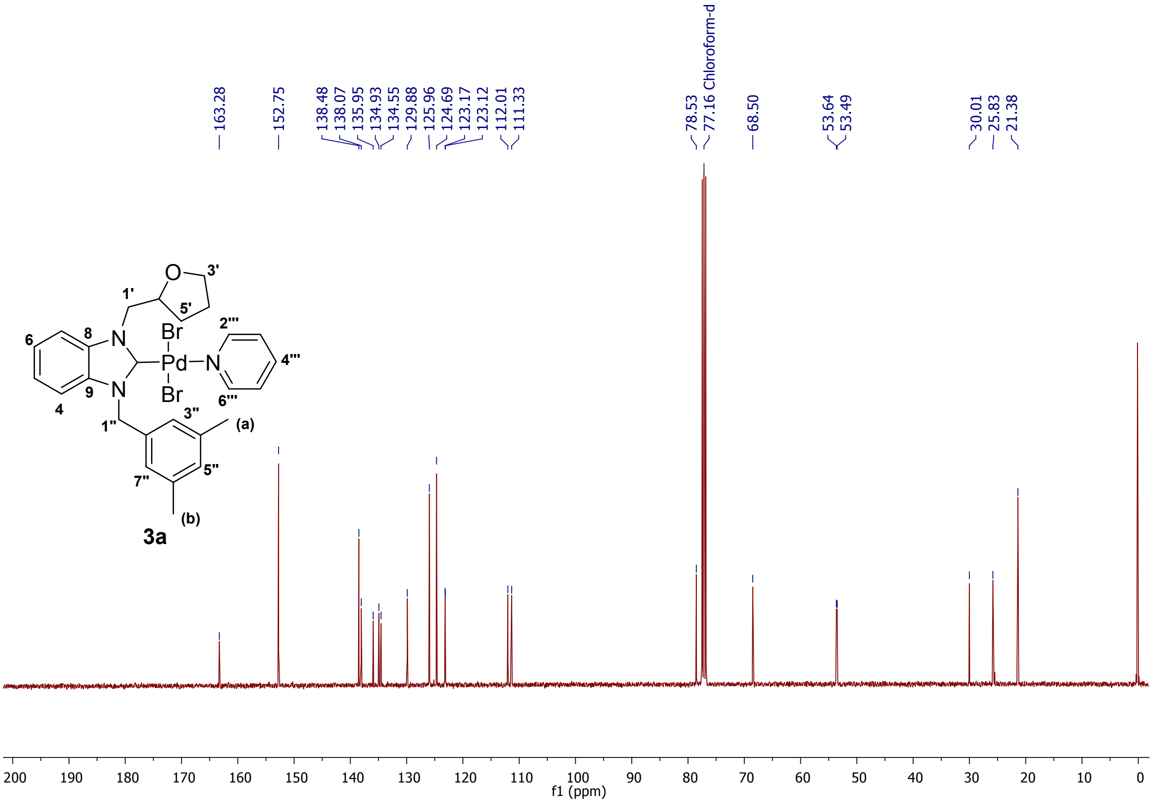

Figure S. 12 ^{13}C NMR spectrum of complex 3a ($CDCl_3$, 100 MHz)

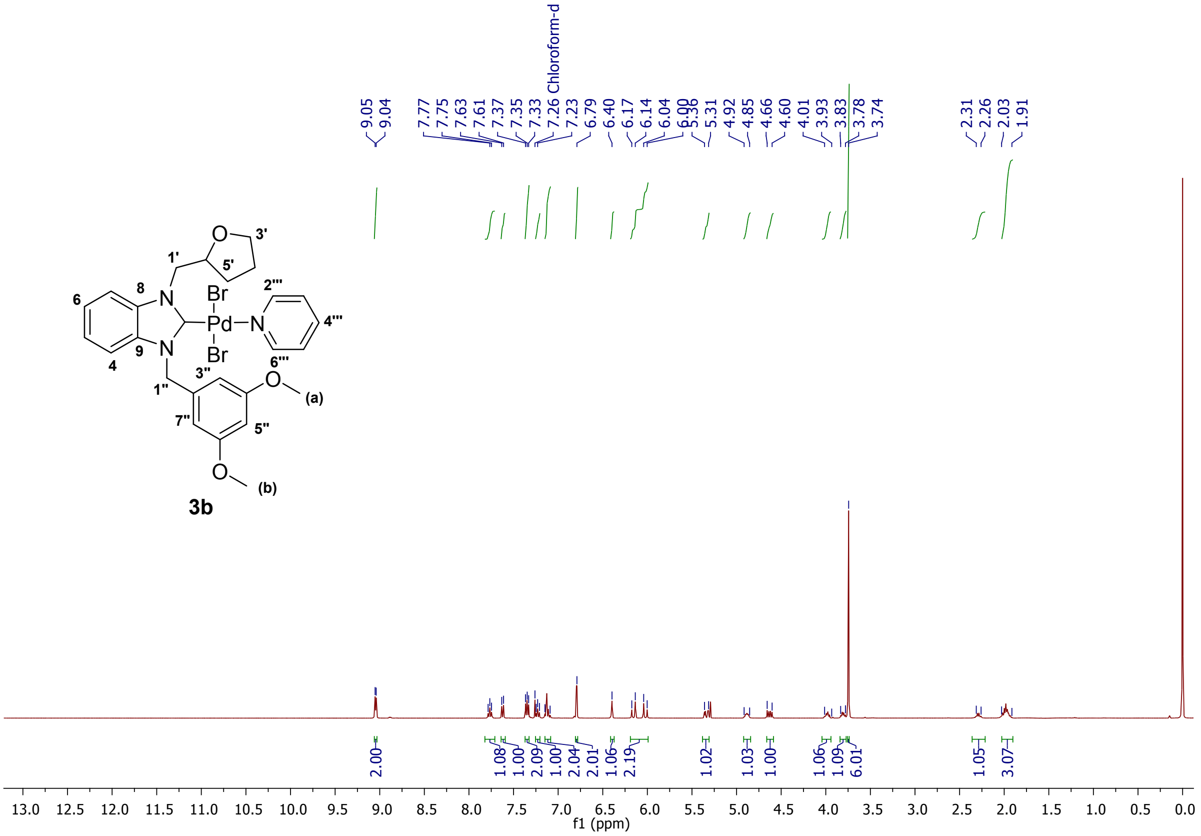

Figure S. 13 ^{1}H NMR spectrum of complex 3b ($CDCl_3$, 400 MHz)

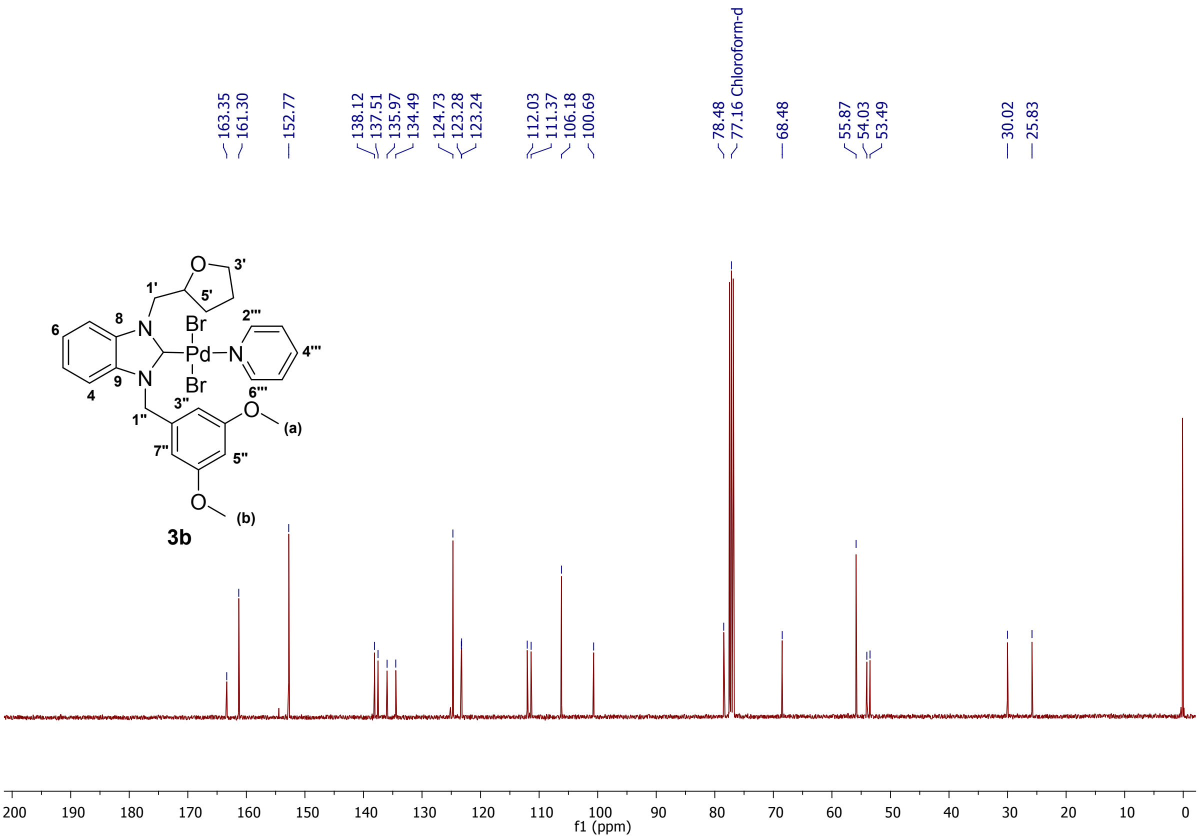

Figure S. 14 ^{13}C NMR spectrum of complex 3b ($CDCl_3$, 100 MHz)

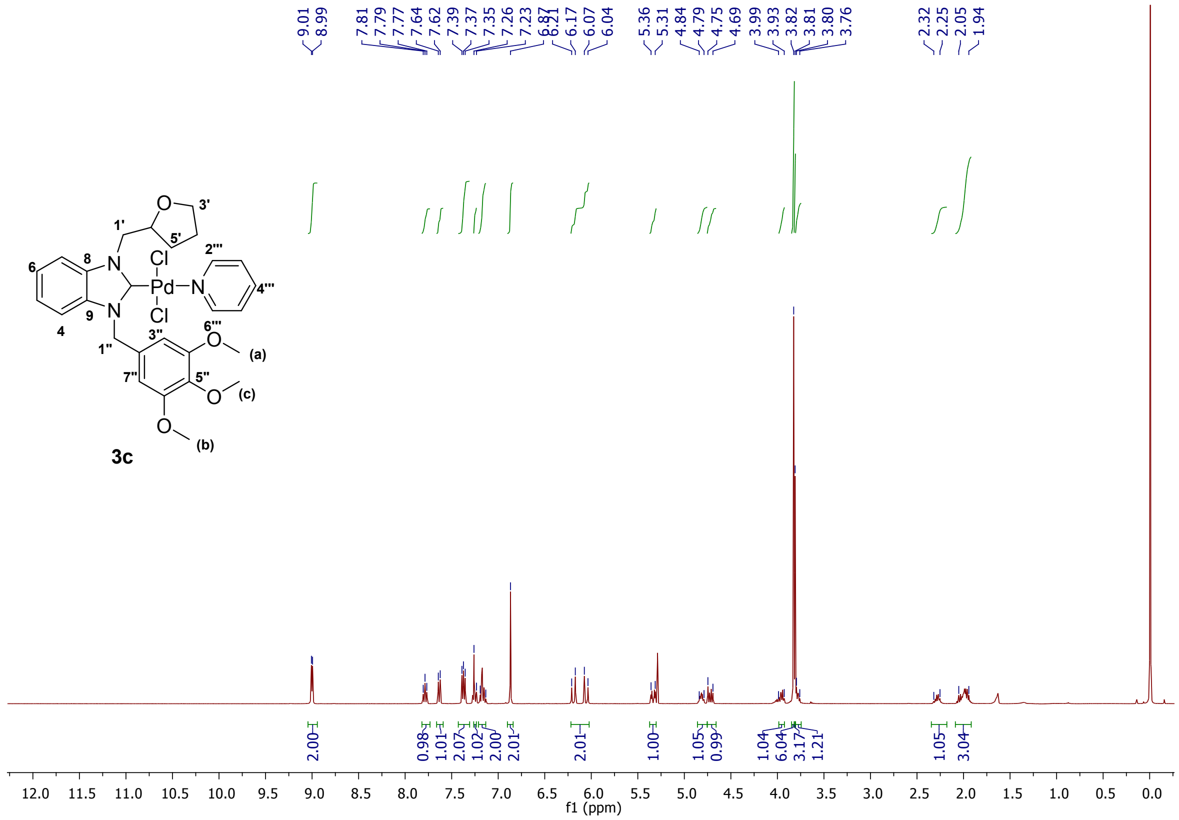

Figure S. 15 ^{1}H NMR spectrum of complex 3c ($CDCl_3$, 400 MHz)

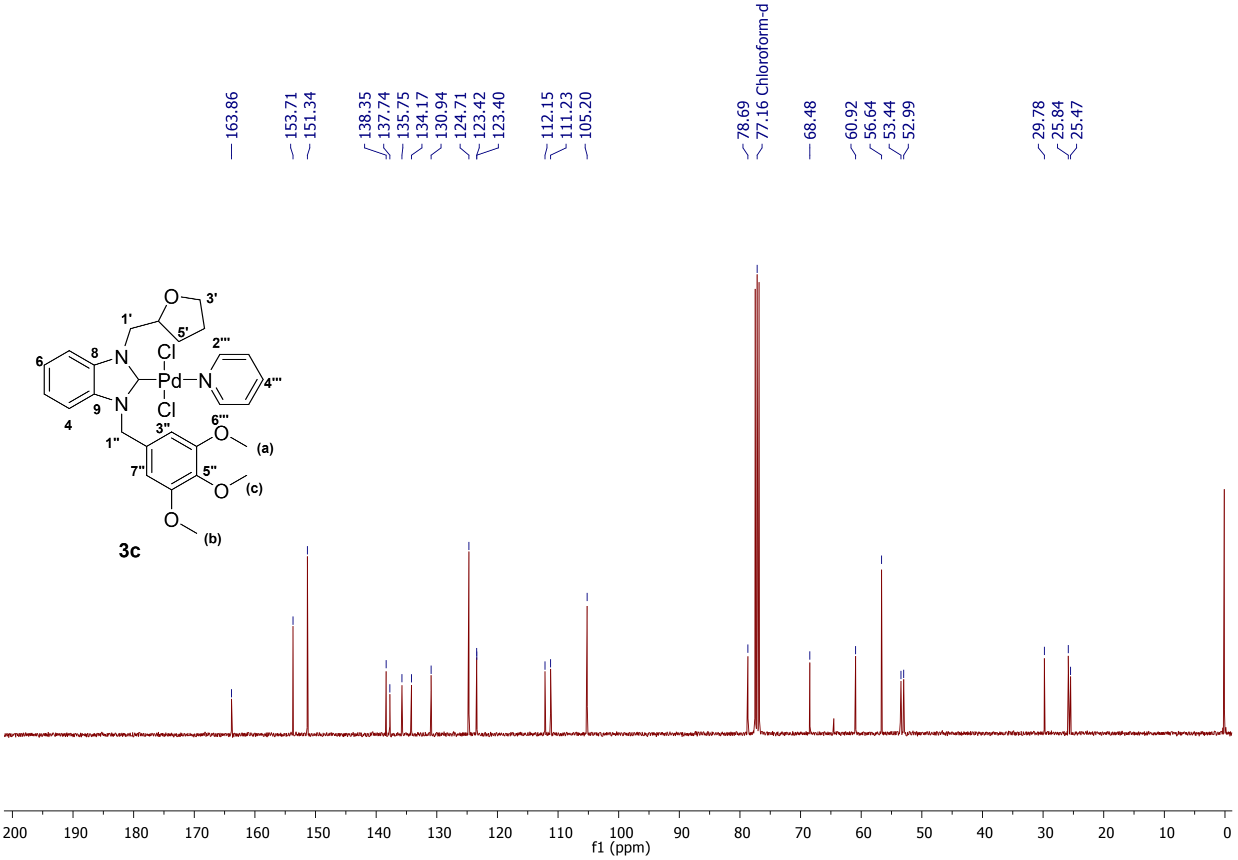

Figure S. 16 ^{13}C NMR spectrum of complex 3c ($CDCl_3$, 100 MHz)

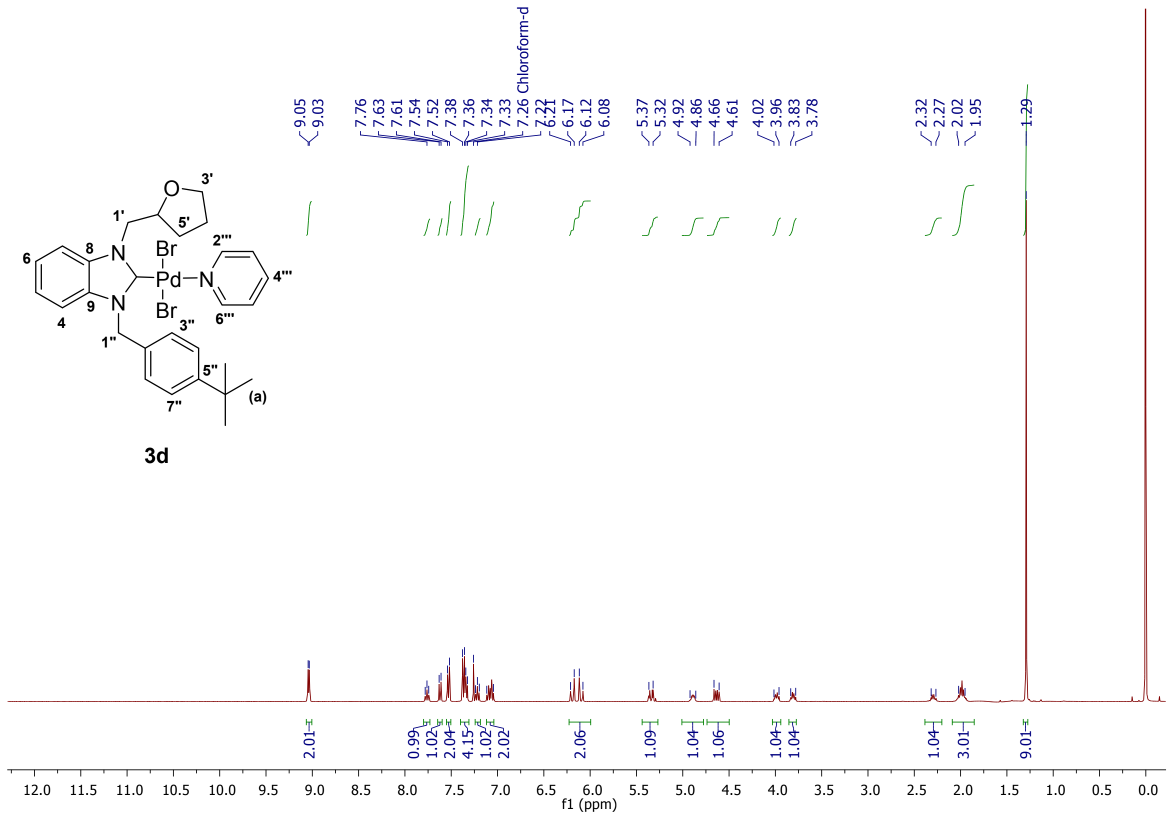

Figure S. 17 ^{1}H NMR spectrum of complex 3d ($CDCl_3$, 400 MHz)

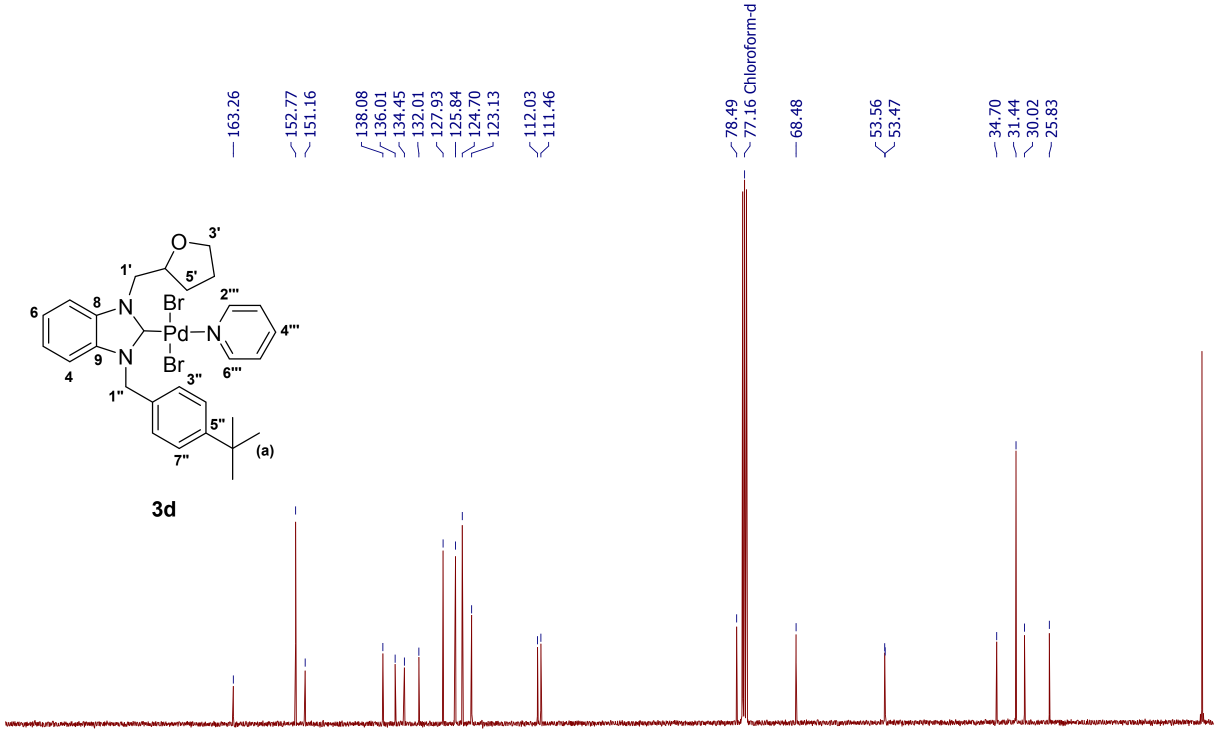

Figure S. 18 ^{13}C NMR spectrum of complex 3d ($CDCl_3$, 100 MHz)

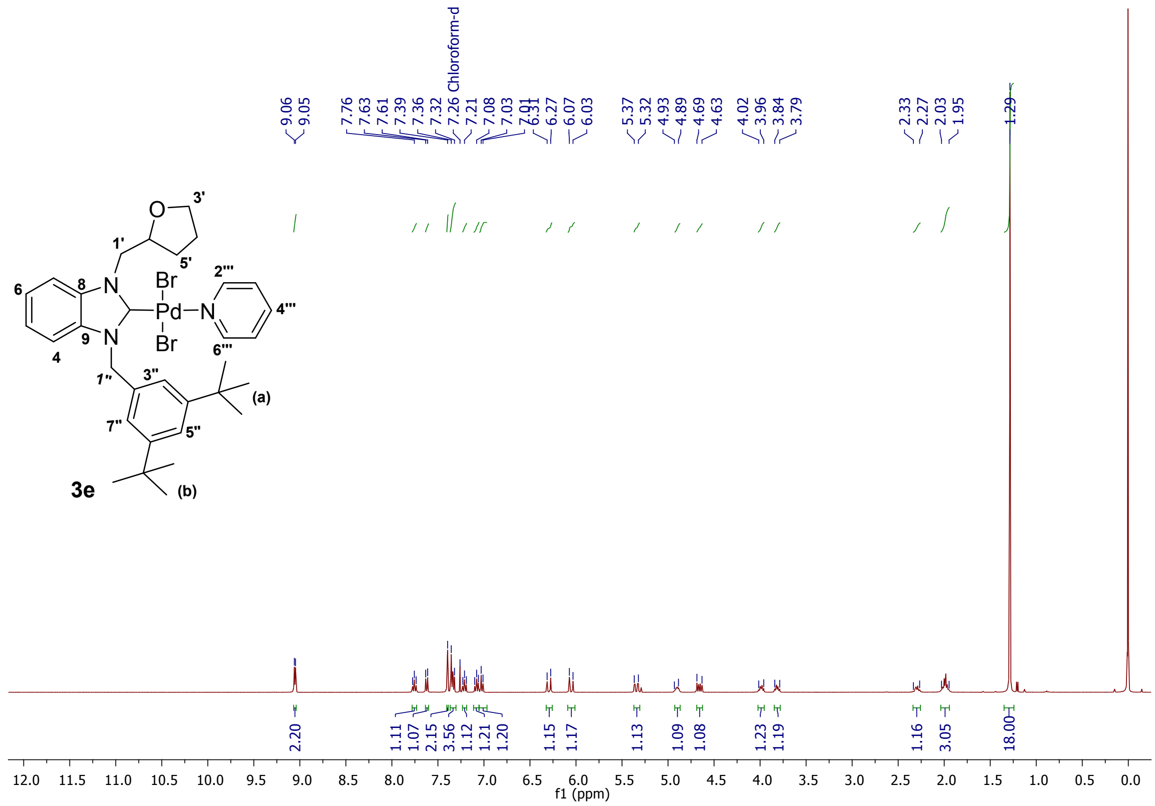

Figure S. 19 ^{1}H NMR spectrum of complex 3e ($CDCl_3$, 400 MHz)

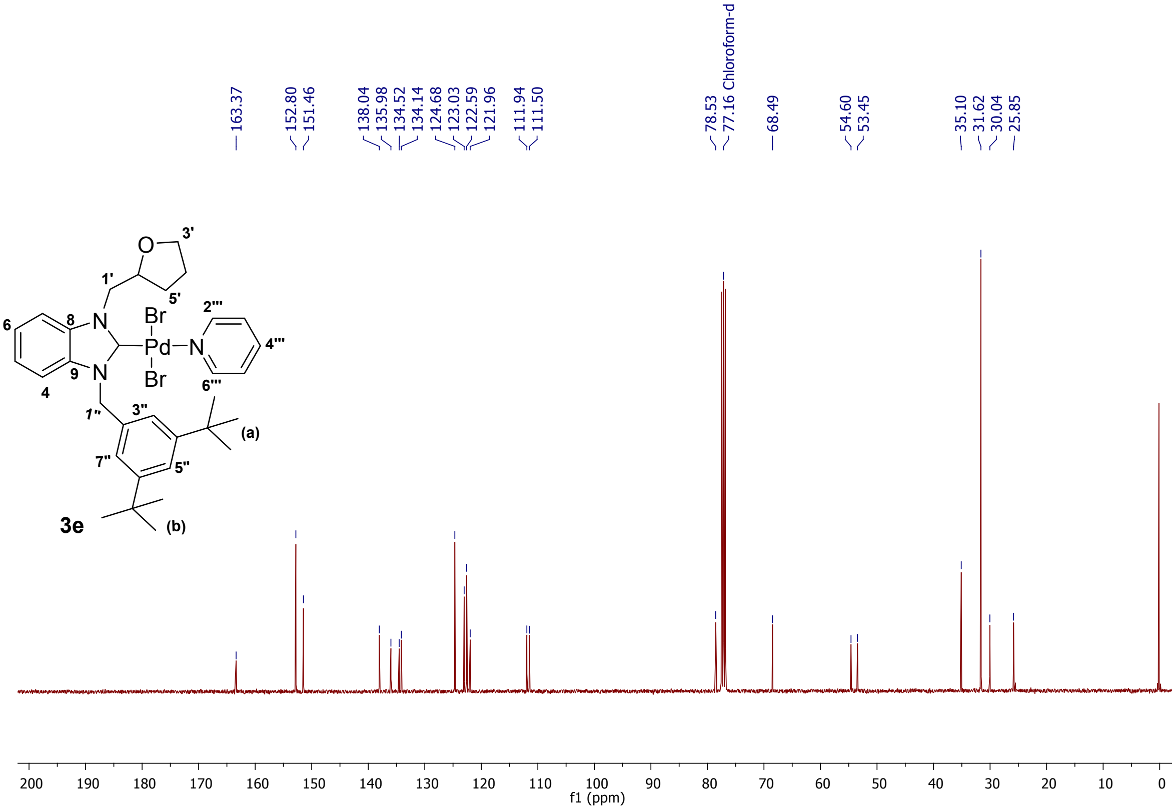

Figure S. 20 ^{13}C NMR spectrum of complex 3e ($CDCl_3$, 100 MHz)

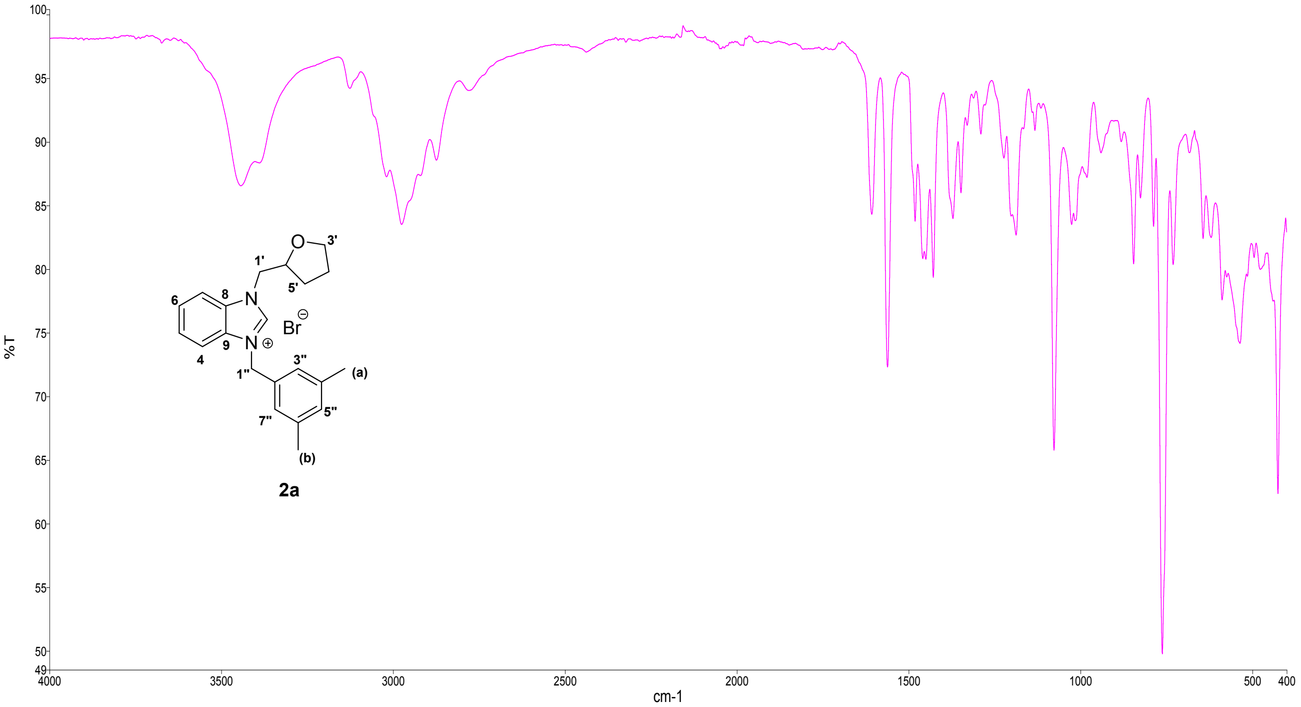

Figure S. 21 FT-IR spectrum of salt 2a

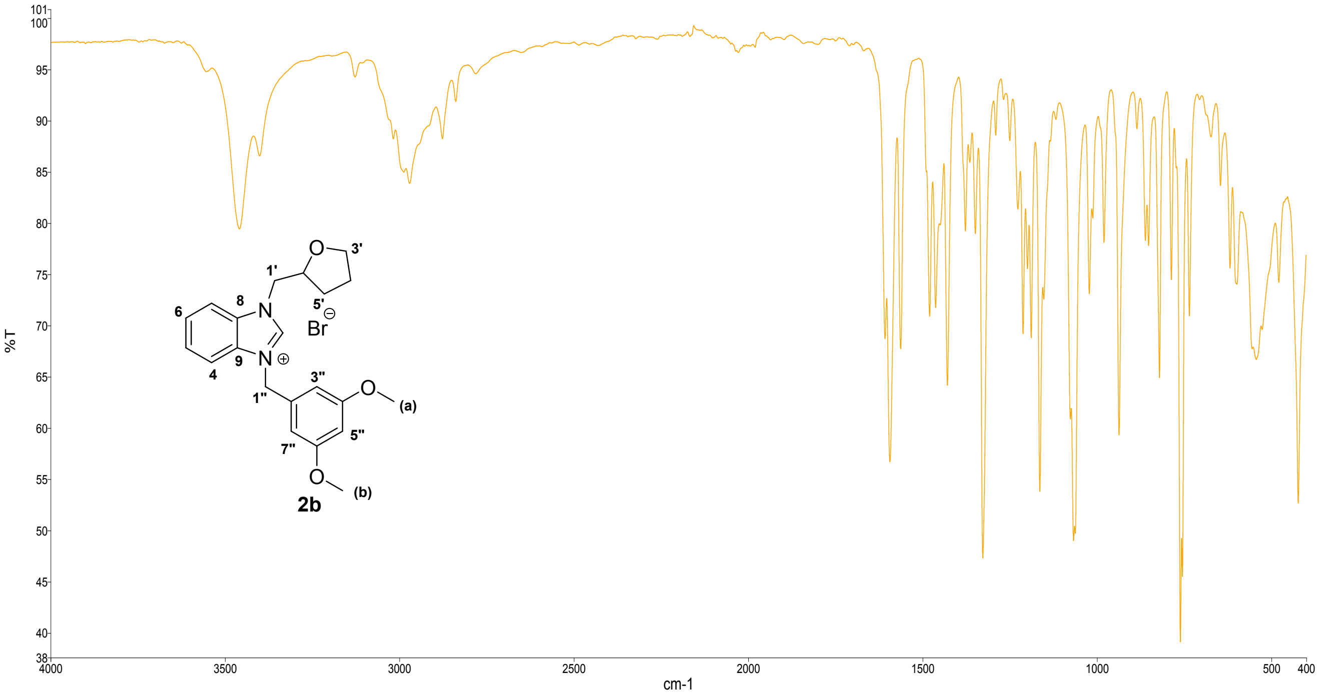

Figure S. 22 FT-IR spectrum of salt 2b

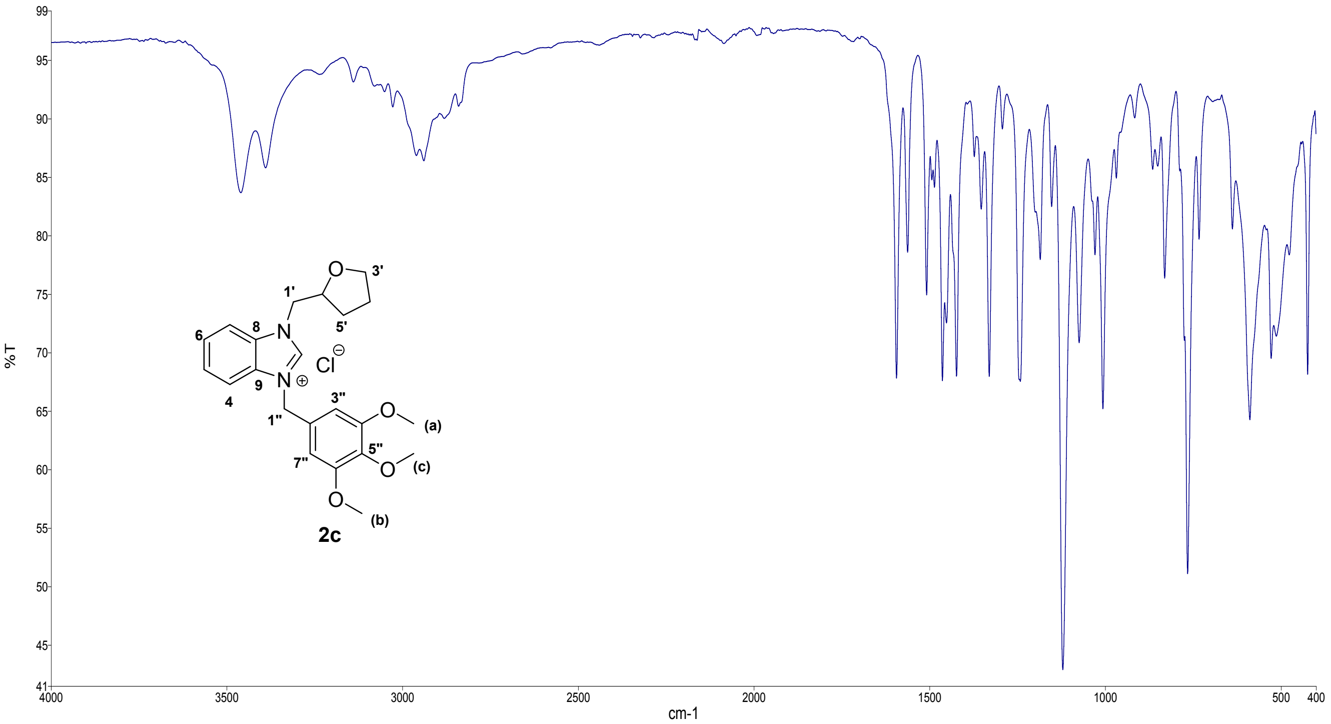

Figure S. 23 FT-IR spectrum of salt 2c

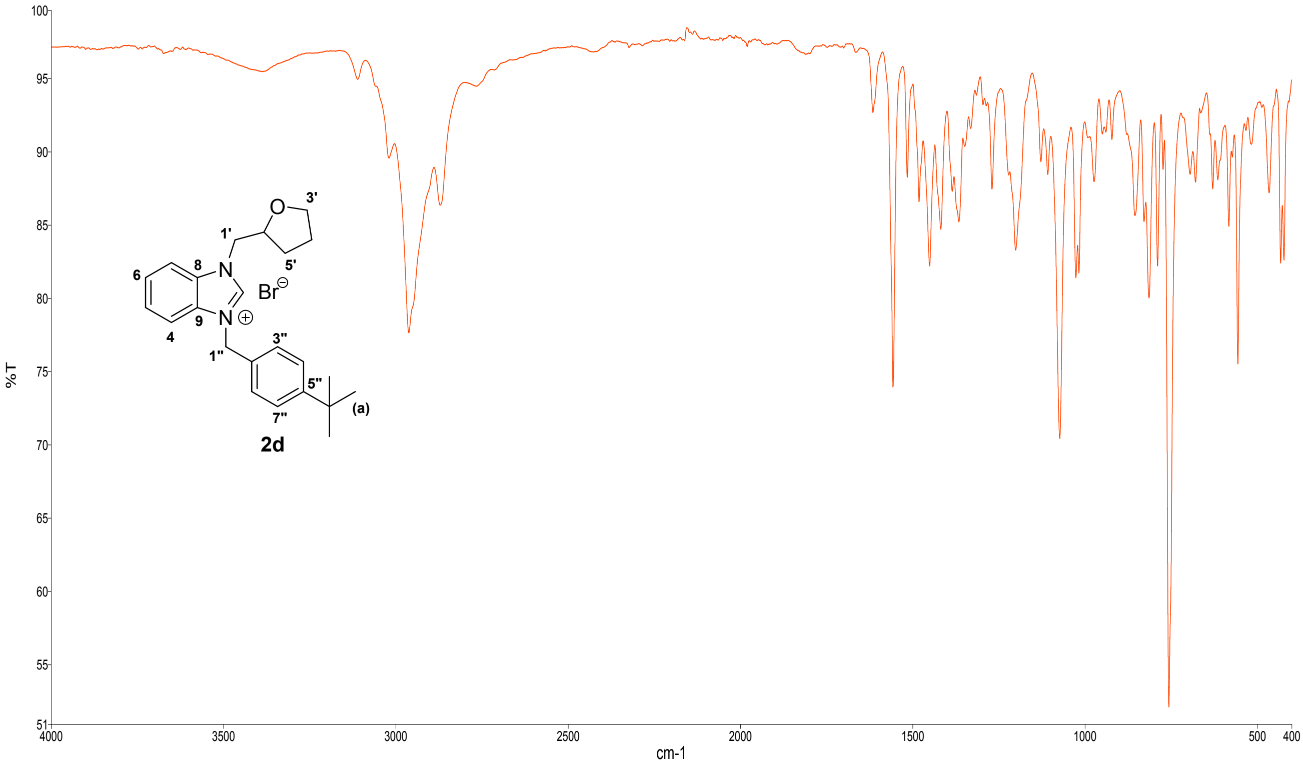

Figure S. 24 FT-IR spectrum of salt 2d

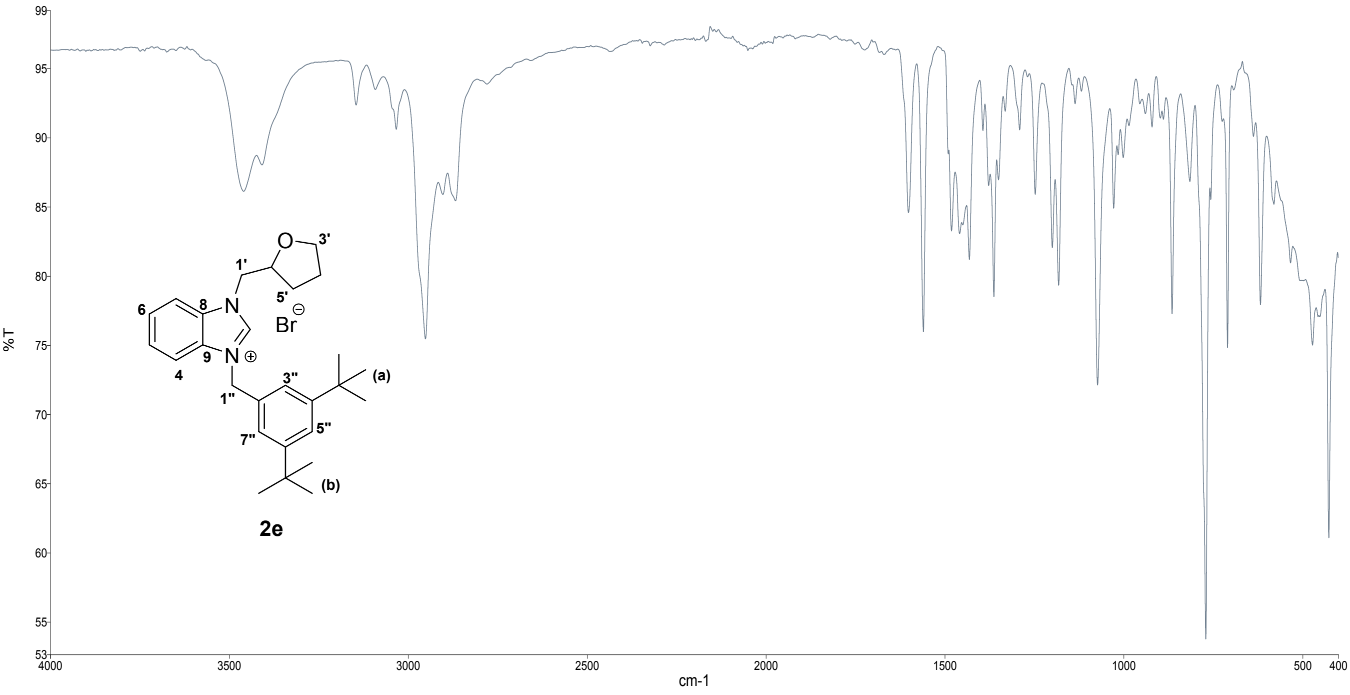

Figure S. 25 FT-IR spectrum of salt 2e

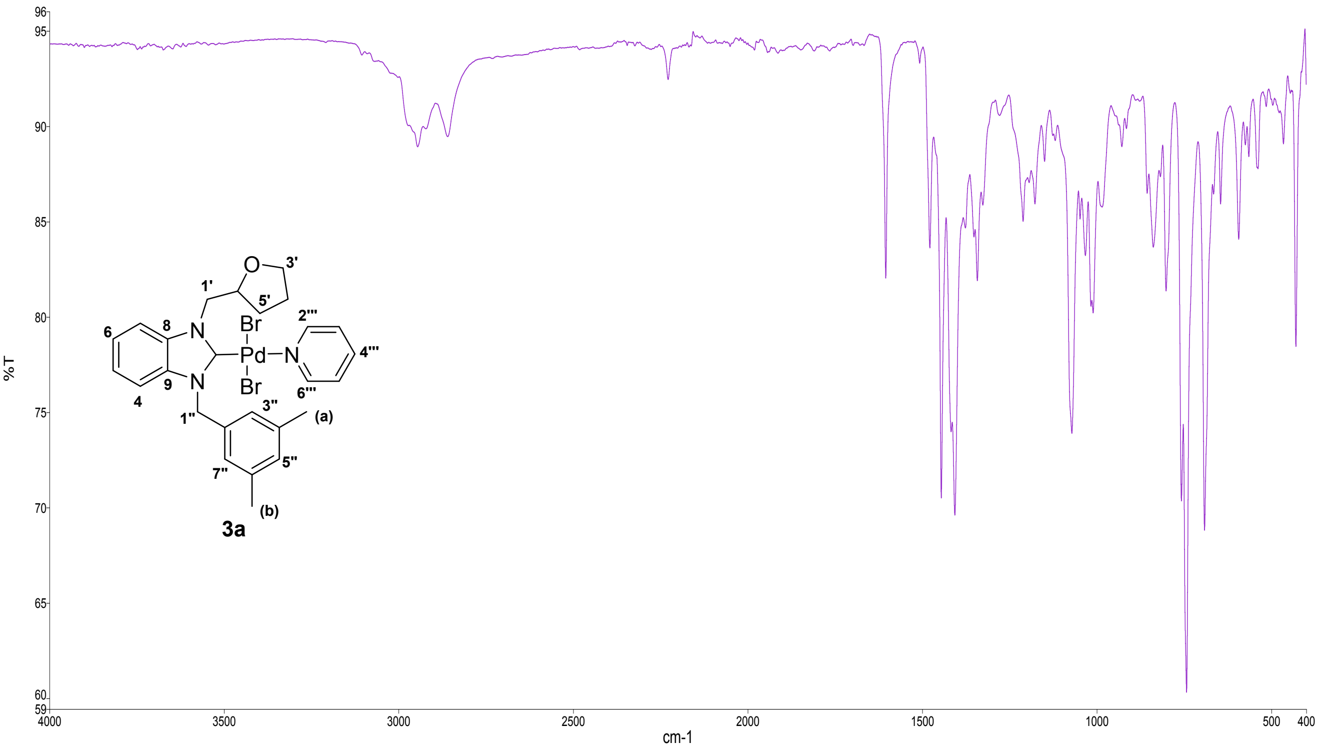

Figure S. 26 FT-IR spectrum of complex 3a

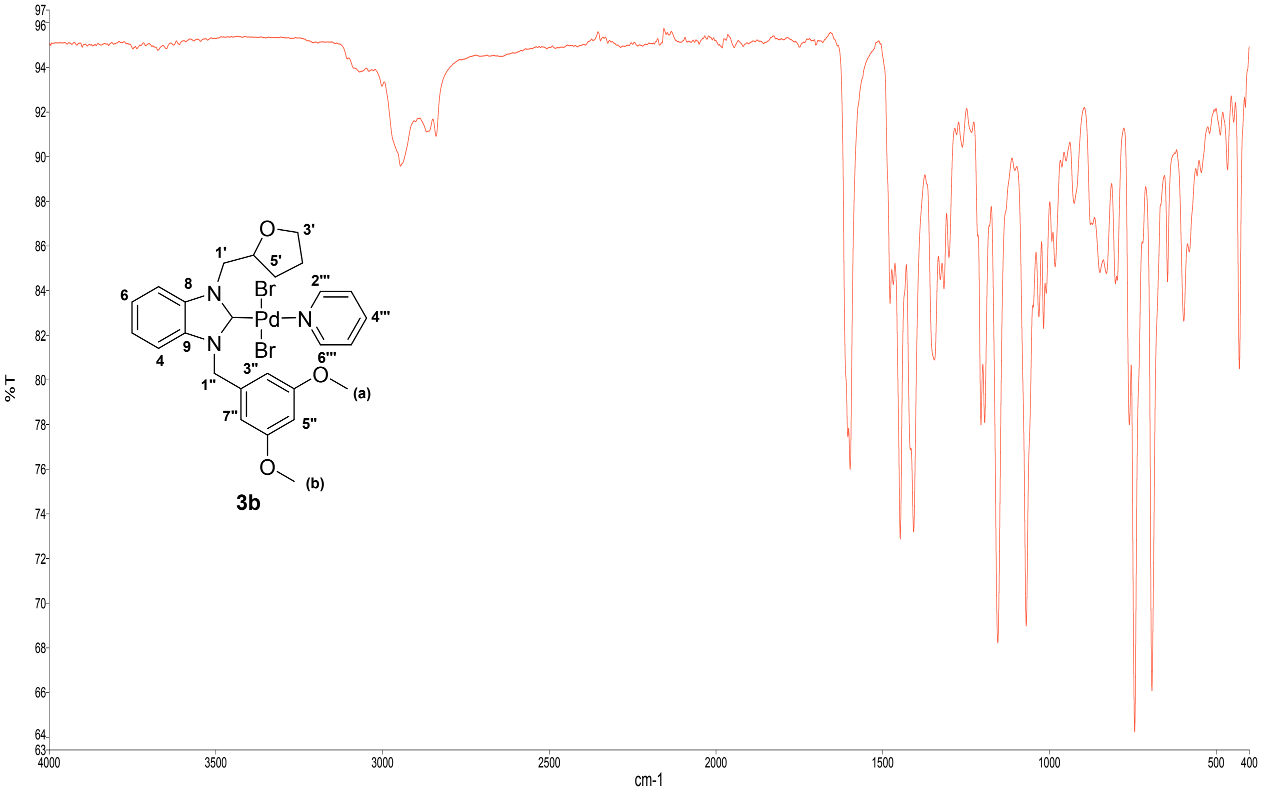

Figure S. 27 FT-IR spectrum of complex 3b

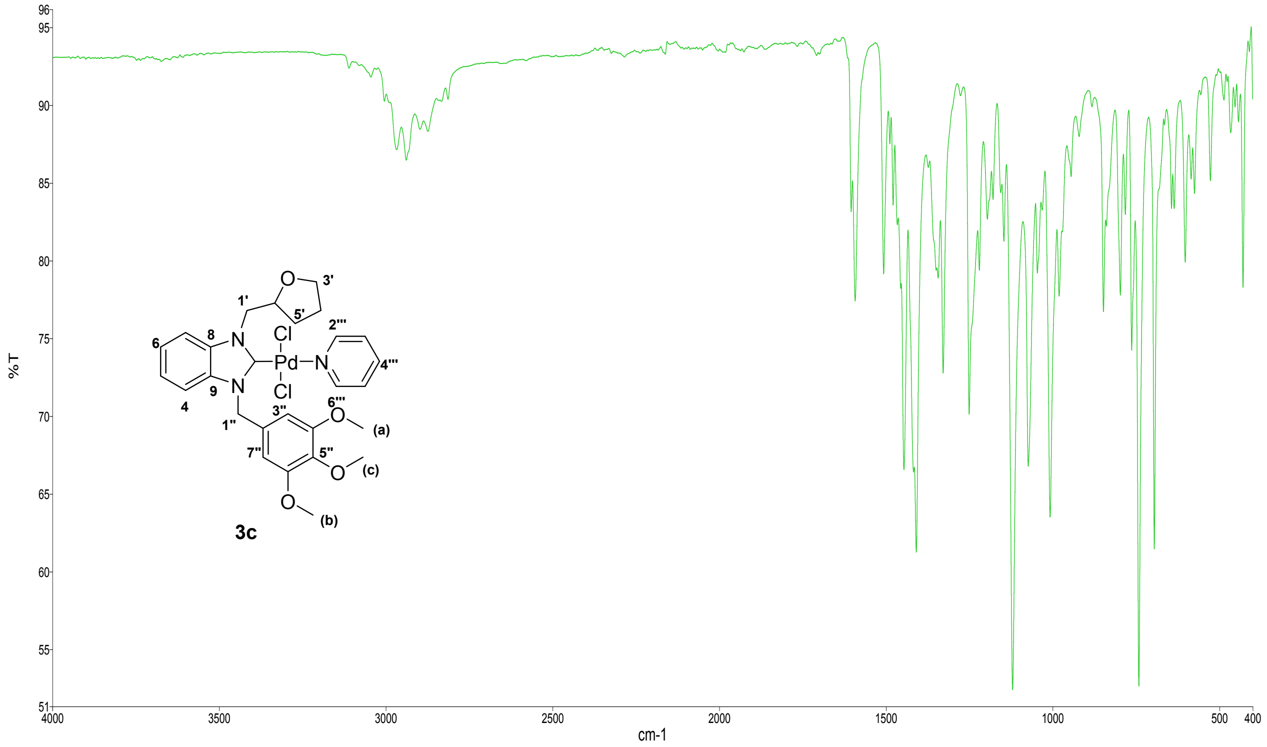

Figure S. 28 FT-IR spectrum of complex 3c

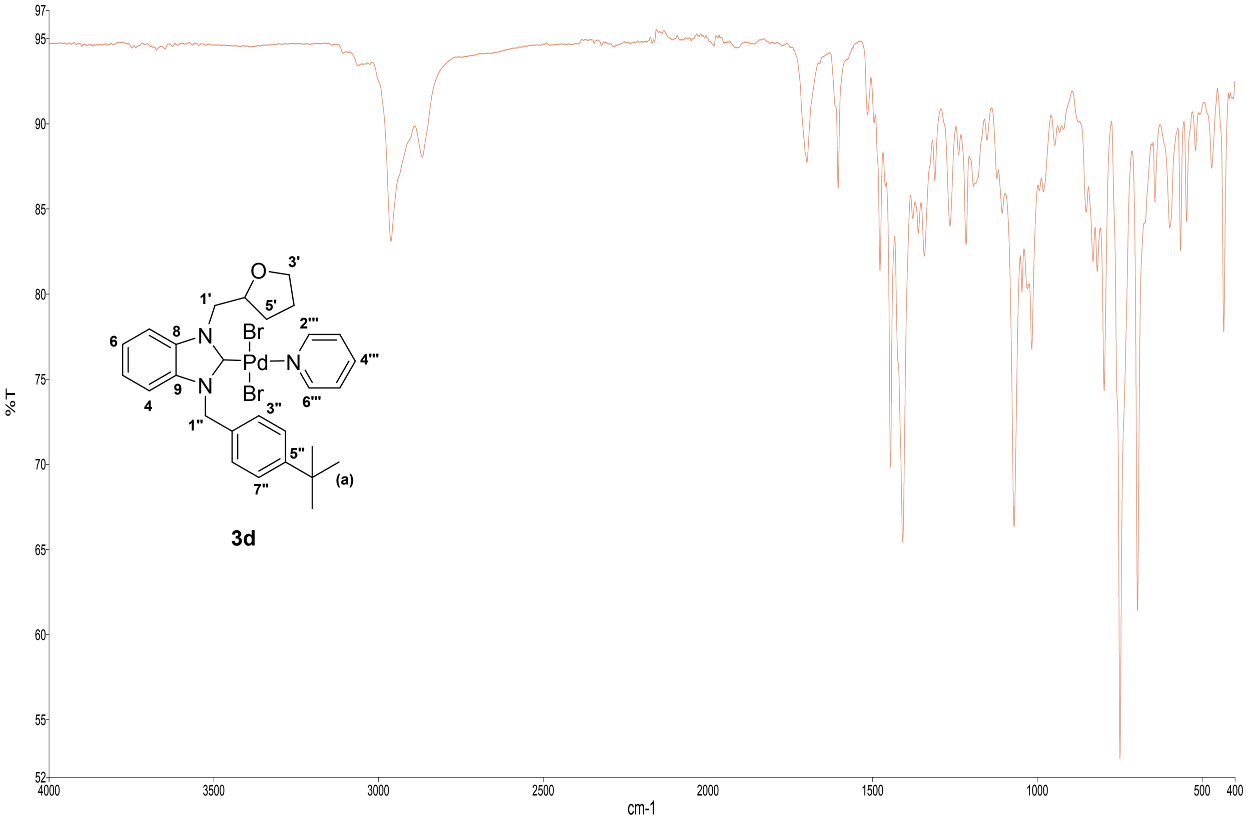

Figure S. 29 FT-IR spectrum of complex 3d

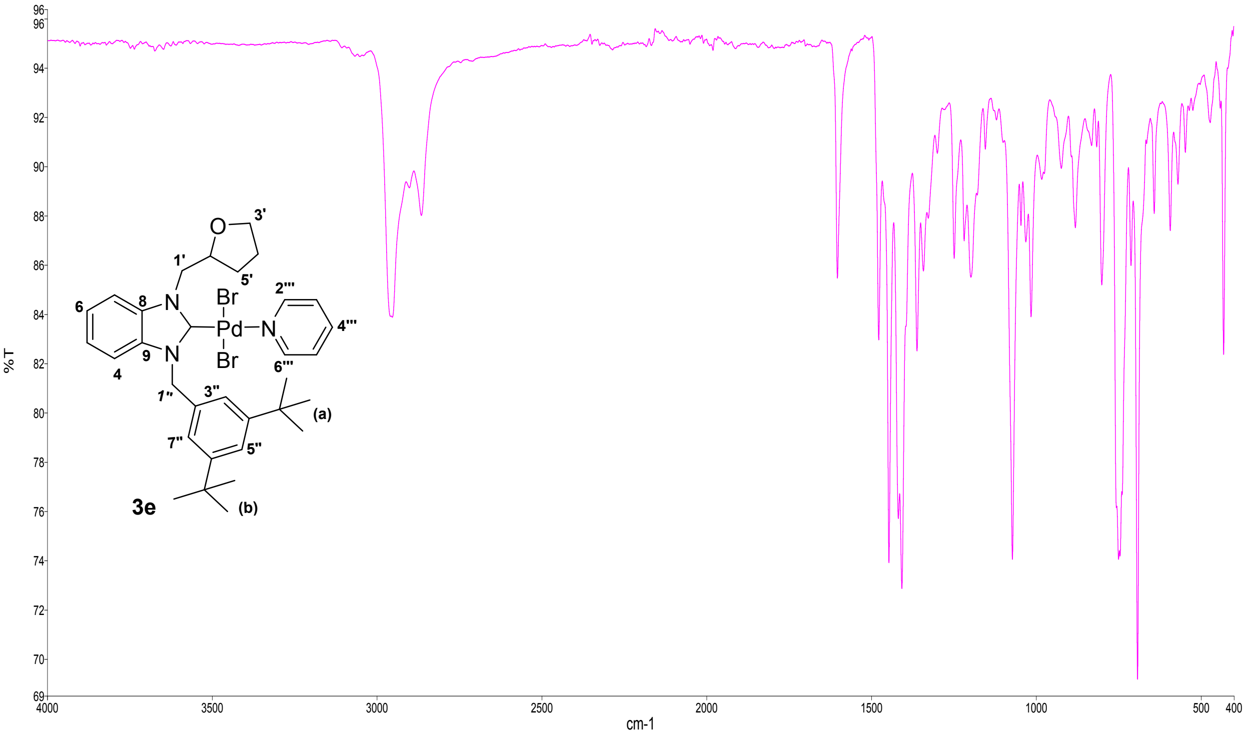

Figure S. 30 FT-IR spectrum of complex 3e

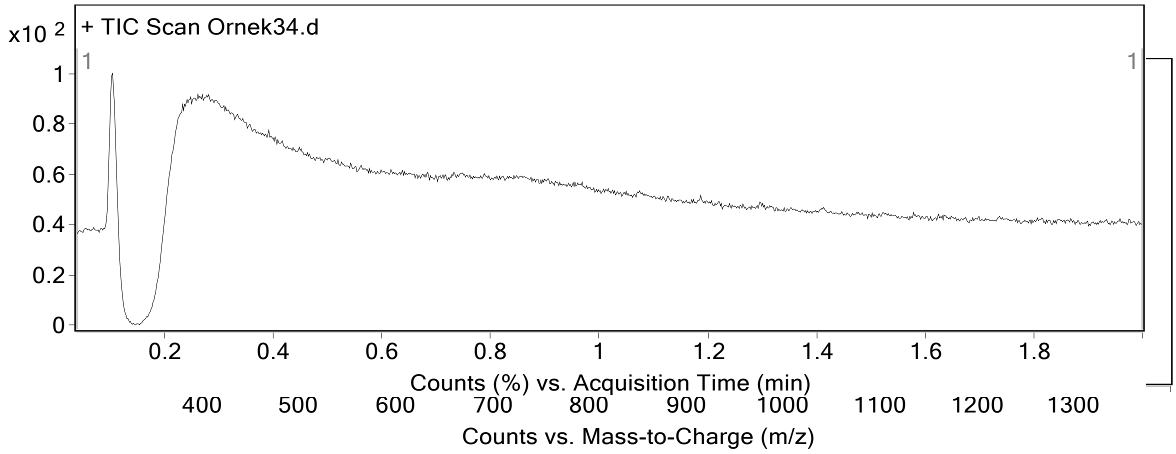

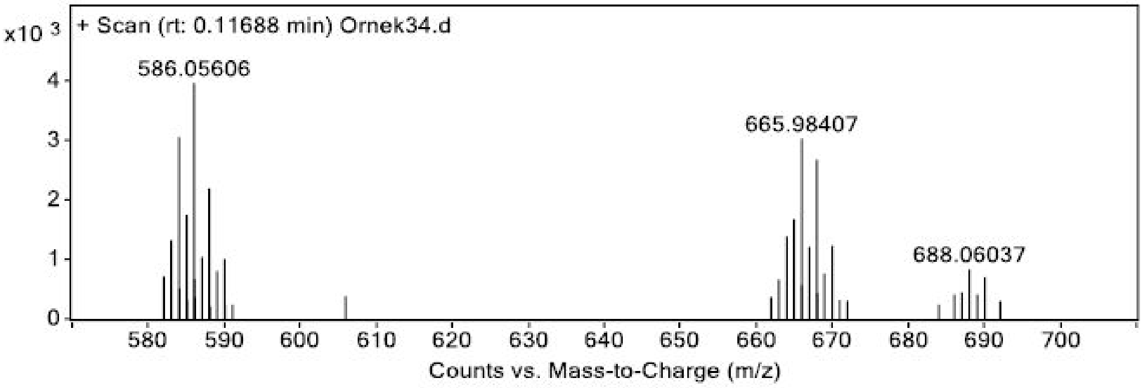

Figure S. 31 HRMS spectrum of complex 3a

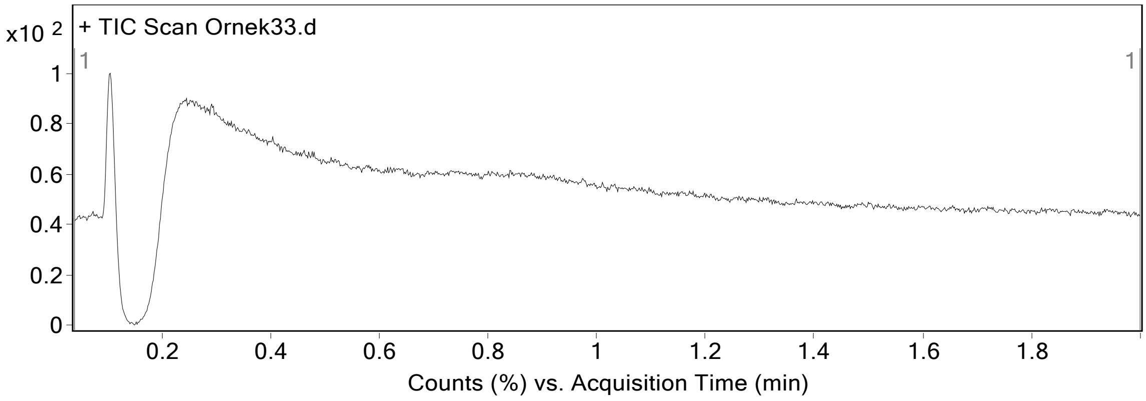

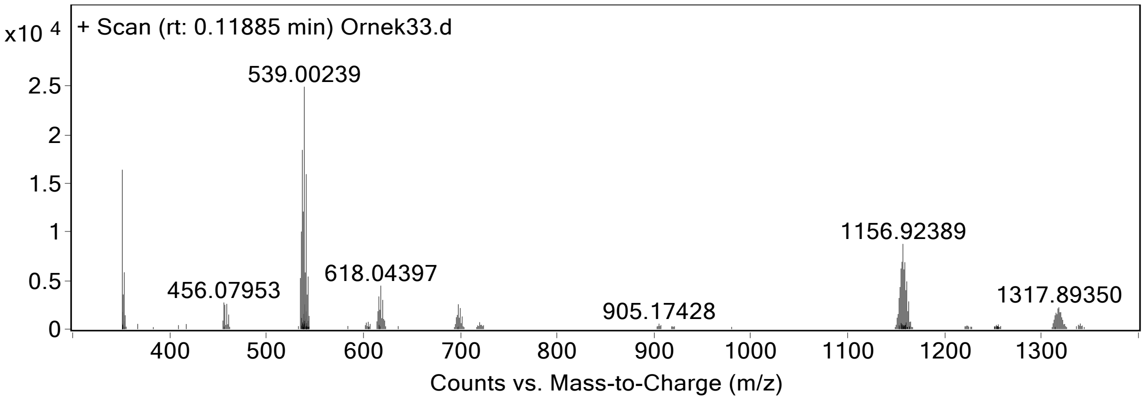

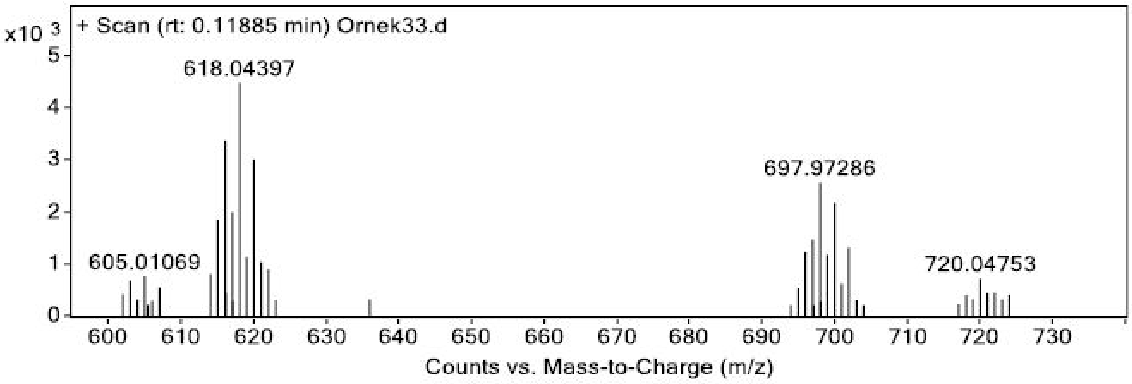

Figure S. 32 HRMS spectrum of complex 3b

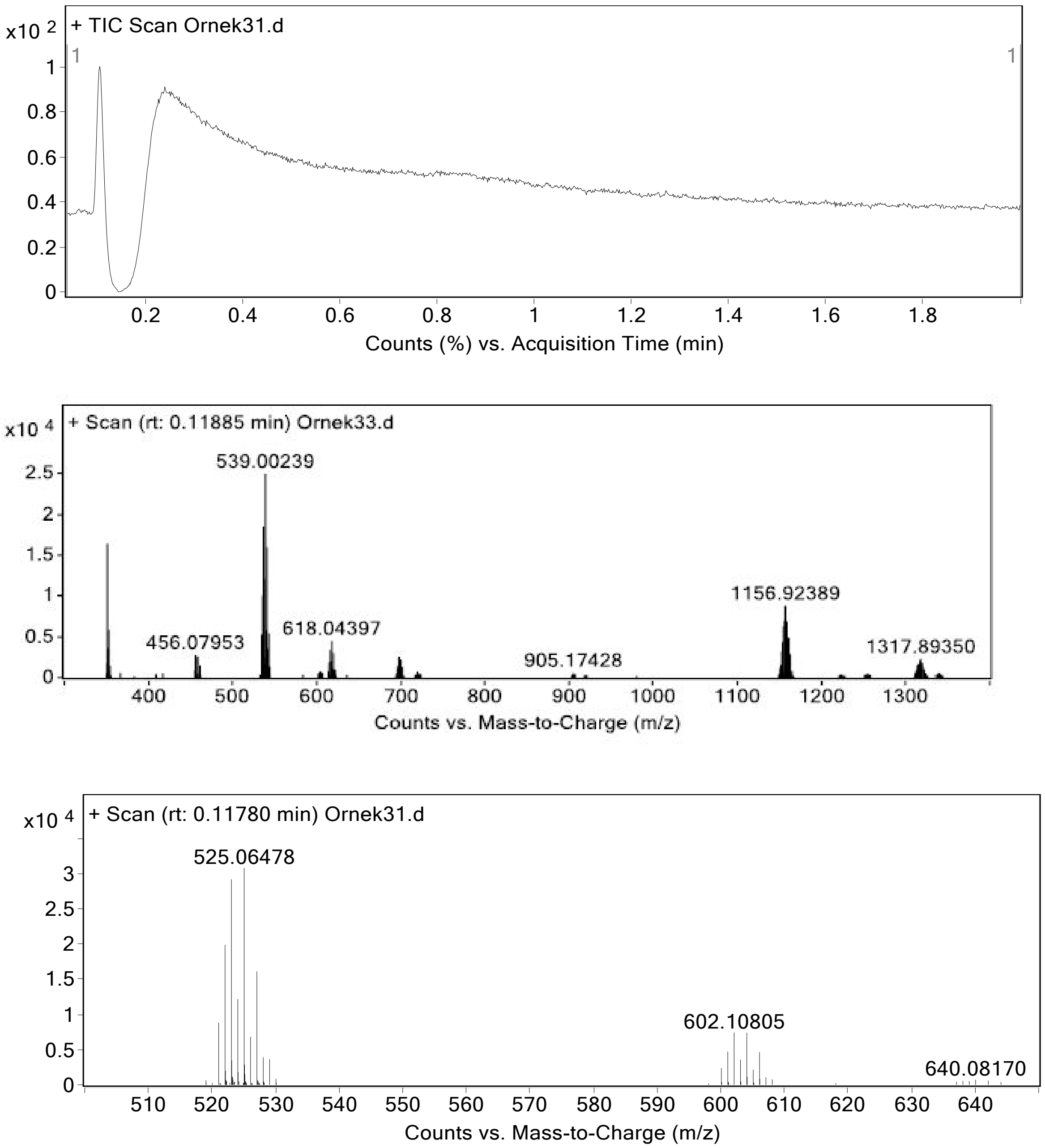

Figure S. 33 HRMS spectrum of complex 3c

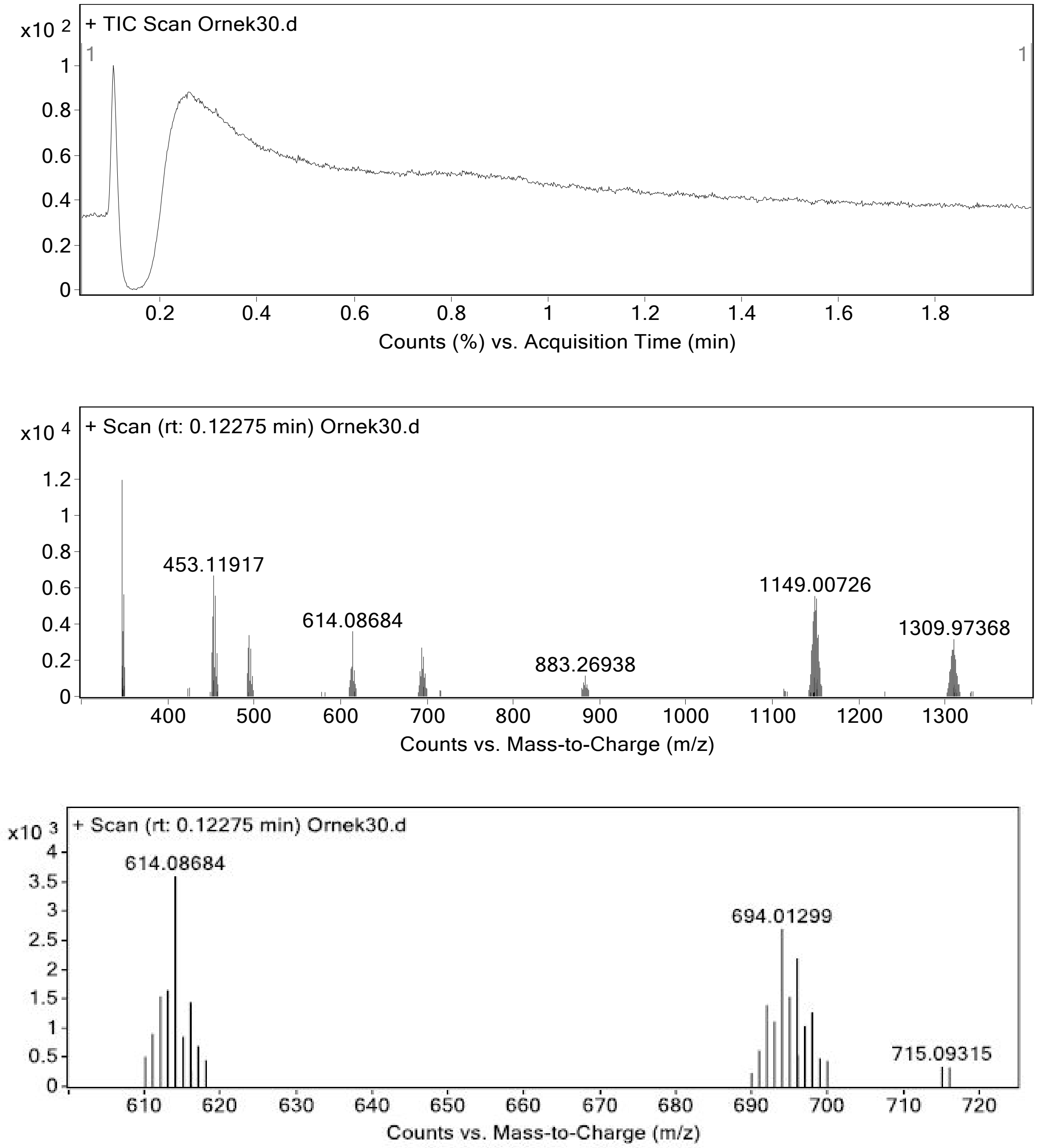

Figure S. 34 HRMS spectrum of complex 3d

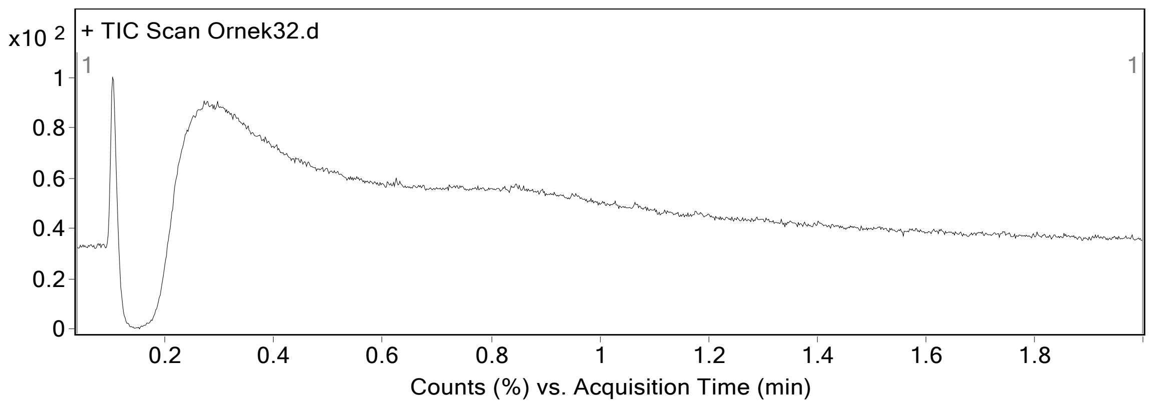

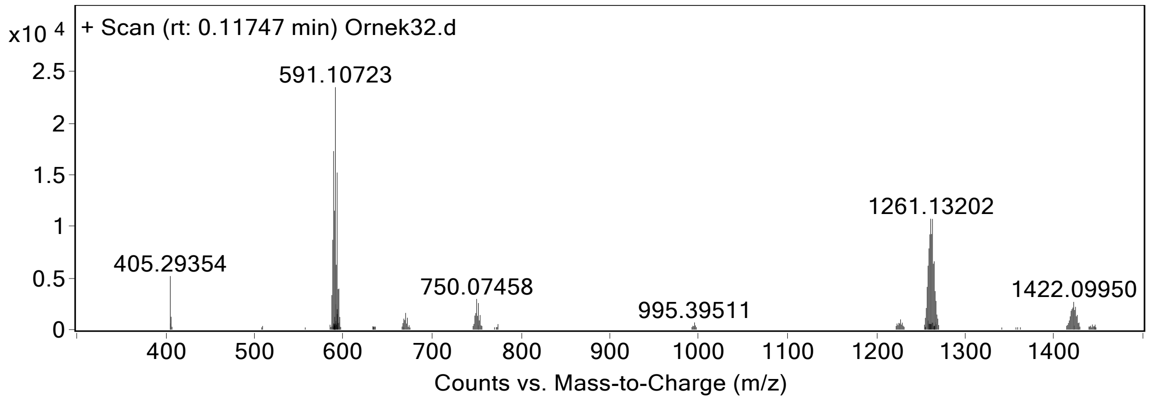

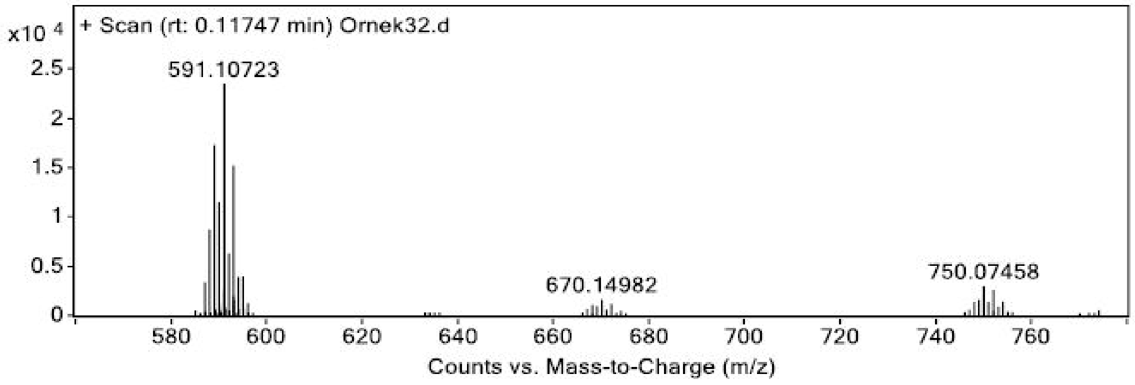

Figure S. 35 HRMS spectrum of complex 3e

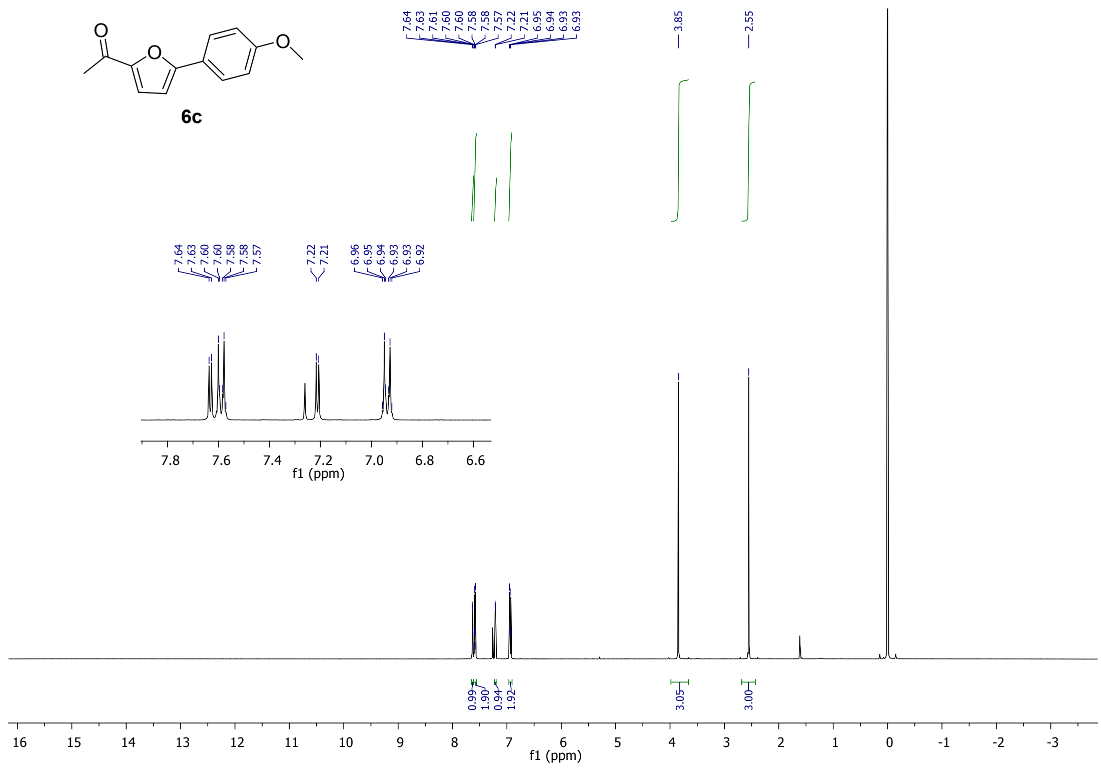

Figure S. 36 ^{1}H NMR spectrum of pruduct 6c ($CDCl_3$, 400 MHz)

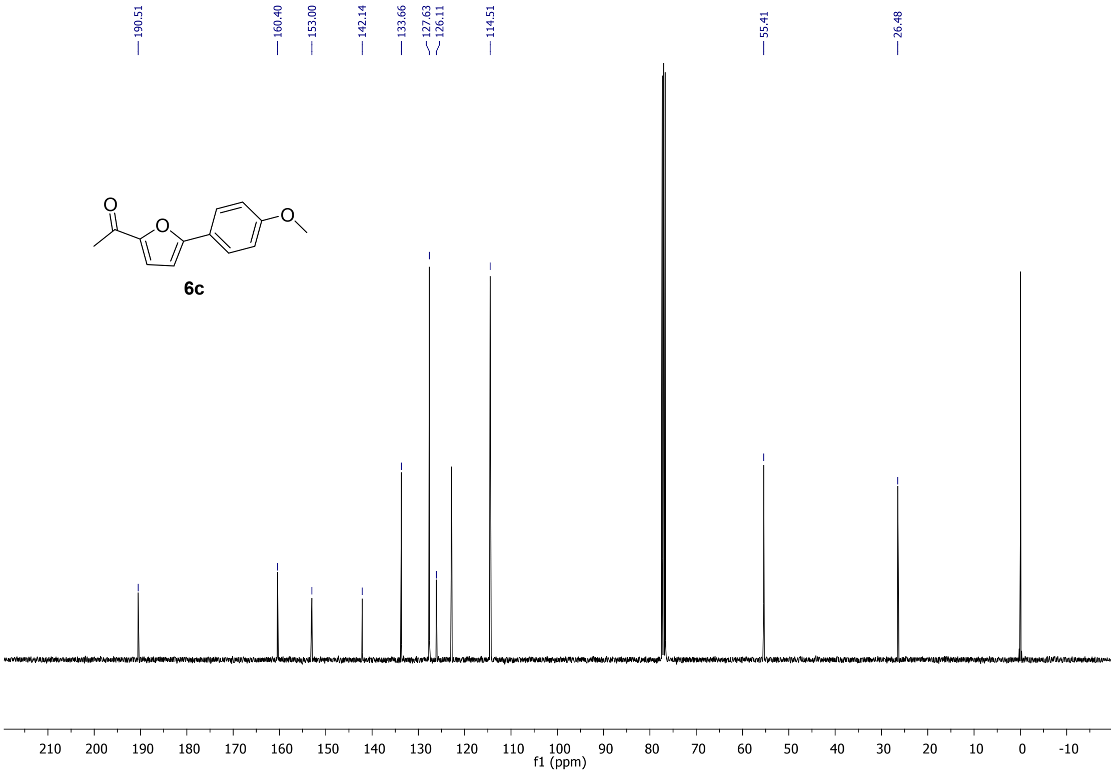

Figure S. 37 ^{13}C NMR spectrum of product 6c ($CDCl_3$, 100 MHz)

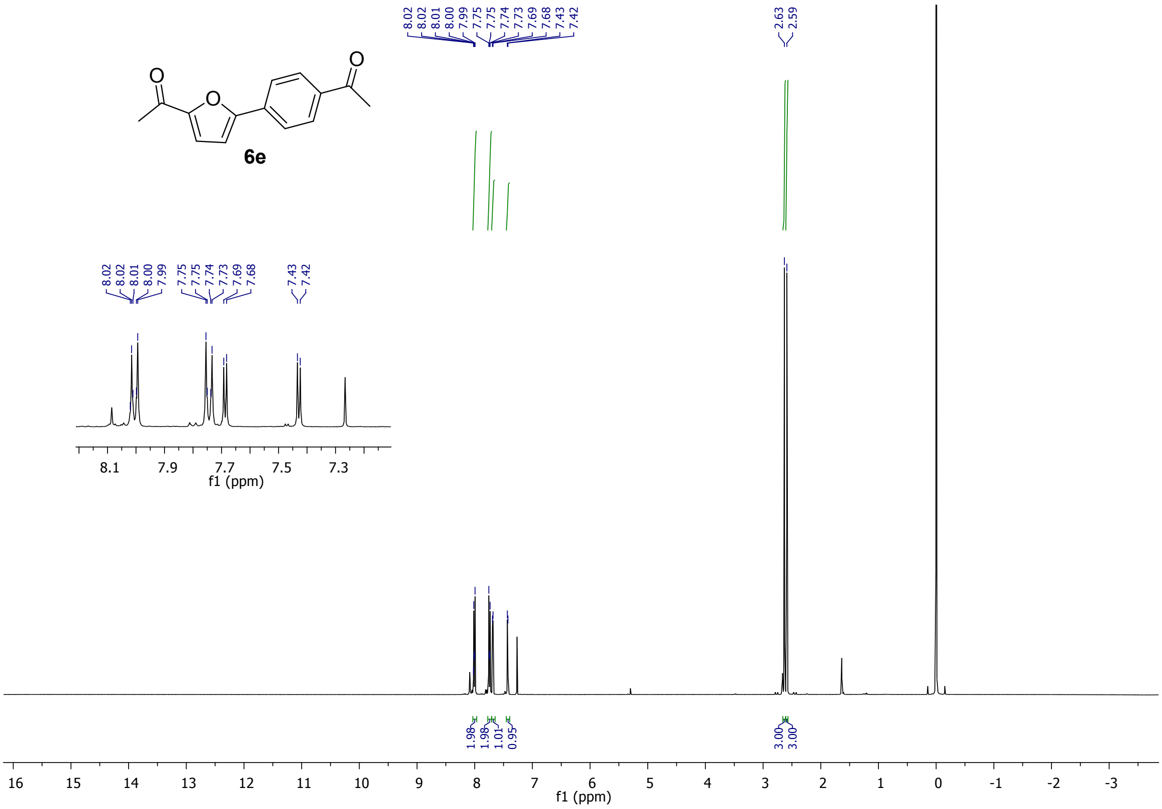

Figure S. 38 ^{1}H NMR spectrum of product 6e ($CDCl_3$, 400 MHz)

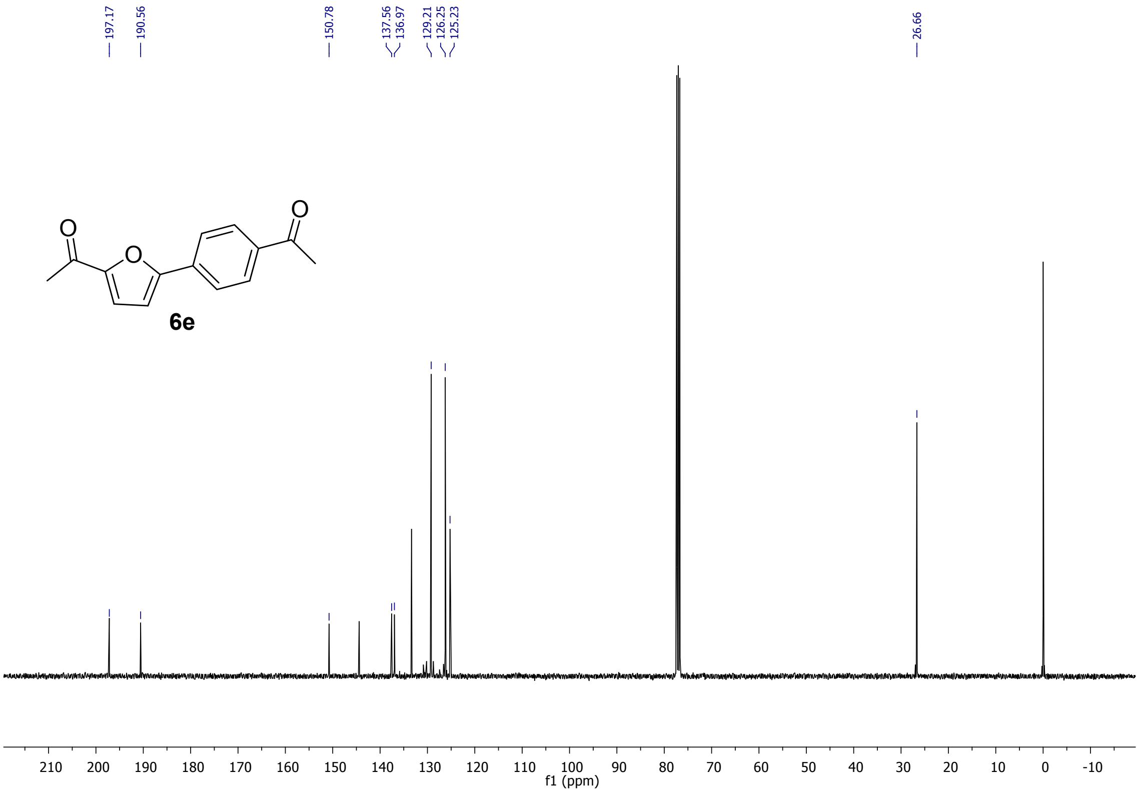

Figure S. 39 ^{13}C NMR spectrum of product 6e ($CDCl_3$, 100 MHz)

NMR Data of arylation's products:

1-(5-Phenyl-furan-2-yl)ethanone (6a) [1]: ^{1}H NMR (300 MHz, $CDCl_3$): δ = 7.85-7.77 (m, 2H, arom.), 7.53-7.37 (m, 3H, arom.), 7.29 (d, 1H, arom. CH, J = 2.9 Hz), 6.82 (d, 1H, arom. CH, J = 2.9 Hz), 2.56 (s, 3H, C(=O)CH3) ppm.

1-(5-*p*-Tolyl-furan-2-yl)ethanone (6b) [2]: ^{1}H NMR (300 MHz, $CDCl_3$): δ = 7.71 (d, 2H,arom. CH, J = 8.3 Hz), 7.27 (d, 2H, arom. CH, J = 8.3 Hz), 7.25 (d, 1H, arom. CH, J = 2.9 Hz), 6.83 (d, 1H, arom. CH, J= 2.9 Hz), 2.52 (s, 3H, C(=O)CH3), 2.38 (s,C6H4C*H*3) ppm.

1-(5-(4-Methoxyphenyl)-furan-2-yl)ethanone (6c) [3]: ^{1}H NMR (400 MHz, $CDCl_3$): δ = 7.63 (d, 1H, arom. CH, J = 4.0 Hz), 7.57 (d, 2H, arom. CH, J = 8.0 Hz), 7.21 (d, 1H, arom. CH, J = 4.0 Hz), 6.92 (d, 2H, arom. CH, J = 3.1 Hz), 3.85 (s, 3H, OCH3), 2.55 (s, 3H, C(=O)CH3) ppm.

4-(5-Acetylfuran-2-yl)benzaldehyde (6d) [4]: ^{1}H NMR(400 MHz, $CDCl_3$): 10.02 (1H, s), 7.81 (2H, d, J = 8.9 Hz), 7.66 (2H, d, J = 7.0 Hz), 7.34 (1H, d, J = 3.5 Hz), 7.28 (1H, d, J = 4.0 Hz), δ 2.53 (3H, s,C(=O)CH3).

1-(4-(5-Acetylfuran-2-yl)phenyl)ethanone (6e) [3]: ^{1}H NMR (400 MHz, $CDCl_3$): δ = 7.99 (d, 2H, arom. CH, J = 8.0 Hz), 7.73 (d, 2H, arom. CH, J = 4.0 Hz), 7.68 (d, 1H, arom. CH, J = 4.0 Hz), 7.42 (d, 1H, arom. CH, J = 4.0 Hz), 2.63 (s, 3H, C6H4-C(=O)C*H*3), 2.59 (s, 3H, C4H2O-C(=O)C*H*3) ppm.

1-(5-(4-fluorophenyl)furan-2-yl)ethanone (6f) [5]: ^{1}H NMR (400 MHz, $CDCl_3$): δ= 7.80 – 7.74 (m, 2H), 7.25 (d, J = 3.7 Hz, 1H), 7.15 – 7.09 (m, 2H), 6.71 (d, J = 3.7 Hz, 1H), 2.52 (s, 3H).

1-(5-(Naphthalen-1-yl)-furan-2-yl)ethanone (6g) [2]: ^{1}H NMR (300 MHz, $CDCl_3$): δ = 8.12 (d, 1H, arom. CH, J = 8.0 Hz), 7.88 (d, 1H, arom. CH, J = 8.1 Hz), 7.82 (d, 1H, arom. CH, J = 8.1 Hz), 7.68 (d, 1H, arom. CH, J = 8.0 Hz), 7.52-7.44 (m, 3H, arom. CH), 7.32 (d, 1H, arom. CH, J = 3.2 Hz), 6.90 (d, 1H, arom. CH, J = 3.2 Hz), 2.53 (s, 3H, C(=O)CH3) ppm.

4-(5-acetylfuran-2-yl)benzonitrile (6h) [6]: ^{1}H NMR (200 MHz, $CDCl_3$): d 7.90 (d, J = 8.6 Hz, 2 H), 7.68 (d, J = 8.6 Hz, 2 H), 7.23 (d, J = 3.2 Hz, 1 H), 6.87 (d, J = 3.2 Hz, 1 H), 2.54 (s, 3 H).

1-(5-Phenyl-thiophen-2-yl)ethanone (7a) [7]: ^{1}H NMR (300 MHz, $CDCl_3$): δ = 7.81-7.70 (m, 3H, arom.), 7.53-7.31 (m, 2H, arom.), 7.27 (d, 1H, arom. CH, J = 2.8 Hz), 7.20 (d, 1H, arom. CH, J = 2.8 Hz), 2.54 (s, 3H, C(=O)CH3) ppm.

1-(5-*p*-Tolyl-thiophen-2-yl)ethanone (7b) [8]: ^{1}H NMR (300 MHz, $CDCl_3$): δ = 7.65 (d, 1H, arom. CH, J = 3.4 Hz), 7.54 (d, 2H, arom. CH, J = 8.0 Hz), 7.27 (d, 1H, arom. CH, 3*J*HH = 3.4 Hz), 7.22 (d, 2H, arom. CH, J = 8.0 Hz), 2.55 (s, 3H, C(=O)CH3), 2.38 (s, C6H4C*H*3) ppm.

1-(5-(4-Methoxyphenyl)-thiophen-2-yl)ethanone (7c) [9]: ^{1}H NMR (300 MHz, $CDCl_3$): δ = 7.67 (d, 1H, arom. CH, J= 3.0 Hz), 7.62 (d, 2H, arom. CH, J = 7.7 Hz), 7.23 (d, 1H, arom. CH, J = 3.0 Hz), 6.98 (d, 2H, arom. CH, J = 7.7 Hz), 3.81 (s, 3H, OCH3), 2.56 (s, 3H, C(=O)CH3) ppm.

4-(5-acetylthiophen-2-yl)benzaldehyde (7d) [10]: ^{1}H NMR (500 MHz, $CDCl_3$) δ 9.99 (s, 1H), 7.90 (d, J = 7.7 Hz, 2H), 7.76 (d, J = 7.8 Hz, 2H), 7.66 (d, J = 3.9 Hz, 1H), 7.42 (d, J = 3.8 Hz, 1H), 2.55 (s, 3H).

1-(4-(5-Acetylthiophen-2-yl)phenyl)ethanone (7e): [11] ^{1}H NMR (300 MHz, $CDCl_3$): δ = 8.02 (d, 2H, arom. CH, J = 8.1 Hz), 7.69 (d, 2H, arom. CH, J = 8.1 Hz), 7.61 (d, 1H, arom. CH, J = 2.8 Hz), 7.54 (d, 1H, arom. CH, J = 2.8 Hz), 2.61 (s, 3H, C6H4-C(=O)C*H*3), 2.48 (s, 3H, C4H2S-C(=O)C*H*3) ppm.

1-(5-(4-fluorophenyl)thiophen-2-yl)ethan-1-one (7f) [12]: ^{1}H NMR (300 MHz, $CDCl_3$): d = 2.55 (s, 3 H), 7.09 (t, J = 8.5 Hz, 2 H), 7.23 (d, J = 3.9 Hz, 1 H), 7.60 (dd, J = 8.5, 5.0 Hz, 2 H), 7.63 (d, J = 3.9 Hz, 1 H).

1-(5-(Naphthalen-1-yl)-thiophen-2-yl)ethanone (7g):[12] ^{1}H NMR (300 MHz, $CDCl_3$): δ = 8.17 (d, 1H, arom. CH, *J* = 8.0 Hz), 7.93-7.87 (m, 2H, arom. CH), 7.75 (d, 1H, arom. CH, *J* = 3.4 Hz), 7.44-7.62 (m, 4H, arom. CH), 7.29 (d, 1H, arom. CH, *J* = 3.4 Hz), 2.56 (s, 3H, C(=O)CH3) ppm.

4-(5-acetylthiophen-2-yl)benzonitrile (7h) [12]: ^{1}H NMR (300 MHz, $CDCl_3$): d = 2.58 (s, 3 H),7.40 (d, *J* = 3.9 Hz, 1 H), 7.67 (d, *J* = 3.9 Hz, 1 H), 7.69 (d, *J* = 8.5 Hz, 2 H), 7.73 (d, *J* = 8.5Hz, 2 H).

References

[1] Juwaini, N. A. B.; Ng, J. K. P.; Seayad, J. Catalytic regioselective oxidative coupling of furan-2-carbonyls with simple arenes. *ACS Catalysis*. **2012**, *2*, 1787.

[2] Obushak, N. D.; Gorak, Y. I.; Matiichuk, V. S.; Lytvyn, R. Z. Synthesis of heterocycles based on arylation products of unsaturated compounds: XVII. Arylation of 2-acetylfuran and synthesis of 3-R-6-(5-aryl-2-furyl)-7 H-[1, 2, 4] triazolo [3, 4-b][1, 3, 4] thiadiazines. *Russ. J. Org. Chem.* **2008**, *44*, 1689.

[3] Ghosh, D.; Lee, H. M. Efficient Pd-catalyzed direct arylations of heterocycles with unreactive and hindered aryl chlorides. *Org. Lett*. **2012**, *14*, 5534.

[4] Nawaz, Z.; Gürbüz, N.; Zafar, M. N.; Tahir, M. N.; Ashfaq, M.; Karci, H.; & Özdemir, İ. Direct arylation (hetero-coupling) of heteroarenes via unsymmetrical palladium-PEPPSI-NHC type complexes. *Polyhedron*. **2021**,*208*, 115412.

[5] Obushak, N. D.; Gorak, Y. I.; Matiichuk, V. S.; Lytvyn, R. Z. Synthesis of heterocycles based on arylation products of unsaturated compounds: XVII. Arylation of 2-acetylfuran and synthesis of 3-R-6-(5-aryl-2-furyl)-7H-[1,2,4]triazolo[3,4-b][1,3,4]thiadiazines. Russ J Org Chem. **2008**, 44*,* 1689.

[6] Dong, J. J.; Roger, J.; Požgan, F.; & Doucet, H. Low catalyst loading ligand-free palladium-catalyzed direct arylation of furans: an economically and environmentally attractive access to 5-arylfurans. *Green chem*, **2009**, *11*, 1832.

[7] Martina, K.; Baricco, F.; Caporaso, M.; Berlier, G.; Cravotto, G. Cyclodextrin-Grafted Silica-Supported Pd Nanoparticles: An Efficient and Versatile Catalyst for Ligand-Free C− C Coupling and Hydrogenation. *ChemCatChem*, **2016**, *8*, 1176.

[8] Rao, M. L.; Banerjee, D.; Dhanorkar, R. J. Synthesis of functionalized 2-arylthiophenes with triarylbismuths as atom-efficient multicoupling organometallic nucleophiles under palladium catalysis. *Synlett*, **2011**, *2011*, 1324.

[9] Meslin, J. C. ; N'guessan, Y. T. ; Quiniou, H. ; Tonnard, F. Enchainements heteroatomiques et leurs produits de cyclisation—I: Vinylogues de thioamides comme intermediaires de synthese d'acyl-2 thiophenes, thio-1 pyrannones-2 (thiones), dihydro-5, 6 dithiinnes-1, 2 dioxydes-1, 1 et dithiinnes-1, 2 dioxydes-1, 1 substitues. *Tetrahedron*, **1975**, *31*, 2679.

[10] Robbins, D. W.; Hartwig, J. F. AC–H borylation approach to Suzuki–Miyaura coupling of typically unstable 2–heteroaryl and polyfluorophenyl boronates. *Org. Lett.* **2012**,*14*, 4266.

[11] Anderson, E. L.; Casey Jr, J. E.; Emas, M.; Force, E. E.; Jensen, E. M.; Matz, R. S.; & Rivard, D. E. Antiviral activity of glyoxals and derivatives. *J. Med. Chem.***1963**, *6*, 787.

[12] Battace, A.; Lemhadri, M.; Zair, T., Doucet, H., & Santelli, M. Direct Arylation of Thiophenes via Palladium-Catalysed C-H Functionalisation at Low Catalyst Loadings. *Adv. Synth. Catal*. **2007**, *349*, 2507.
